# Recyclable Base-Triggered “Debond-on-Demand” Aliphatic Polyurethane Adhesives: Engineering Adhesion for Use in Inkjet Formulations

**DOI:** 10.1021/acsaenm.5c00390

**Published:** 2025-07-17

**Authors:** Matthew J. Hyder, Jessica Godleman, Andrew Kyriacou, Stuart W. Reynolds, James E. Hallett, Thomas Zinn, Josephine L. Harries, Wayne Hayes

**Affiliations:** † Department of Chemistry, 6816 University of Reading, Whiteknights, Reading RG6 6AD, U.K.; ‡ 260519 Domino UK Ltd, Trafalgar Way, Bar Hill, Cambridge CB23 8TU, U.K.; § Diamond Light Source, 120796 Diamond Light Source Ltd, Harwell Science & Innovation Campus, Didcot OX11 0DE, U.K.

**Keywords:** chain-extended polyurethane, adhesive, debond-on-demand, depolymerization, recycling, inkjet printing

## Abstract

While strong polymeric adhesives are widely valued, their removal can present a significant challenge where substrate recycling is concerned. Recent advancements in “debond-on-demand” adhesives have shown promising enhancements in adhesive strength and debondability. However, they often face a choice between increased adhesive strength or the rate and degree of debonding. Here we report using a rapidly base degradable chain-extender within a series of polyurethanes which possess tailorable adhesive characteristics. These chain-extended polyurethanes (CEPUs) possess high shear strength (8.20 MPa) which upon exposure to base solutions depolymerise (up to 88% loss in *M*_n_) facilitating up to 92% loss in shear strength after only 30 min. Formulation of the CEPUs into inks suitable for continuous inkjet (CIJ) printing produced defined images which upon treatment with base solutions could be removed from the substrate. Having been engineered for circularity, the parent CEPUs can be recycled postdegradation into daughter CEPUs, maintaining their depolymerizable and “debond-on-demand” properties. This work highlights how commercially available starting materials can be utilized to generate highly tailorable polymeric adhesives and inkjet binders capable of rapid depolymerization, ultimately providing an industrially attractive system to increase the recyclability and sustainability of waste materials.

## Graphical abstract

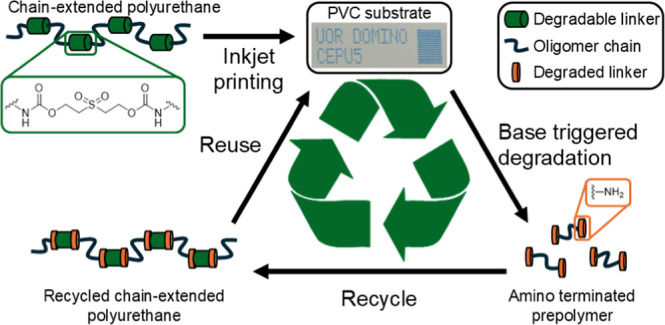

## 1 Introduction

With an ever increasing societal need for sustainability, manufacturers and consumers are driven toward the use of recyclable and reusable materials in everyday commodities such as packaging and plastic bags.^1,2^ Multicomponent packaging can comprise wood products, glass, metal plus varying polymeric materials, such as adhesives, films and containers, all of which serve to complicate the recycling process. The need to readily isolate and recover individual components often adhered to one another therefore requires the use of adhesives which are readily soluble in commercially available solvents or can undergo debond-on-demand processes. Debond-on-demand adhesives are a class of stimuli responsive polymers (SRPs) which can debond from adhered surfaces upon exposure to a specific external physical, biological, or chemical stimuli.^3–5^ This phenomenon occurs through stimuli induced changes in their chemical and physical properties at specific locations within their molecular architecture. Debond-on-demand adhesives have been realized through the use of supramolecular,^6–8^ dynamic covalent bonding,^9,10^ and self-immolative units.^11–14^

Polyurethanes are a versatile group of polymers whose physical and mechanical properties can be tailored for their intended application though appropriate selection of their constituent components.^15^ A diverse range of polyols and polyisocyanates can be used,^16,17^ with further property modifications aided through the introduction of suitable chain-extenders and end-groups. Polyol backbone length and functionality can be used to tailor phase separation or introduce crystallinity into the resulting polyurethane.^18–20^ Supramolecular chain-extenders and cross-linking units^21–23^ and end-groups,^7,24–26^ which utilize hydrogen bonding, π–π stacking, metal–ligand coordination, and host–guest interactions, have been used extensively to make SRPs for use in coatings and adhesives capable of self-healing or participating in debond-on-demand processes.

The recycling of polymers into reusable low molecular weight monomeric units has become a key area of research in recent years,^27–31^ with an ever-increasing shift toward new materials which can go through several recycling cycles without detriment to physical and mechanical properties. Chen and co-workers have reported^32^ a fused five-six bicyclic lactone monomer that can undergo three consecutive polymerization–depolymerization cycles achieving 96–97% isolated yield each cycle, with depolymerization mediated by high temperature thermolysis or via chemolysis using a ZnCl_2_ catalyst at lower temperatures. A racemic mixture of the lactone monomers yielded stereocomplex crystalline polymers which retain their ability to depolymerise efficiently upon treatment with ZnCl_2_.^33^ Dove, Sardon, and co-workers have developed^34^ a method to successfully and selectively depolymerise poly­(ethylene terephthalate) (PET) and bisphenol A-based polycarbonate (BPA-PC) yielding starting monomers and also upcycled cyclic carbonates, ultimately paving the way for a more circular polymeric industry.

Self-immolative polymers are a unique class of SRPs which upon exposure to structure specific stimuli undergo depolymerization to oligomeric^35^ and monomeric^36,37^ units in either a stepwise or concerted manner upon the removal of covalently liable groups.^5^ Self-immolative chemistries frequently originate from atom efficient protecting group methodologies,^38,39^ allowing for the tailoring of depolymerization to specific chemical or physical stimuli.^5^ The use of self-immolative spacers permits the amplification of the reporter release, a common spacer used to this end in polymeric^11,35,40,41^ and dendritic^42,43^ self-immolative systems is 2,6-bis­(hydroxymethyl)-*p*-cresol.

Inkjet printing has a wide range of applications including nanotechnology,^44,45^ electronics,^46^ graphics,^47^ additive manufacturing,^48^ pharmaceuticals,^49^ and tissue engineering,^50,51^ as a mode of precise deposition of materials in a reproducible and highly controlled manner. The use of high-throughput inkjet printing techniques, such as continuous inkjet (CIJ) and drop-on-demand (DOD), for coding and marking objects with information is used in a wide range of industrial settings. The printing of information such as text and barcodes allows for the traceability of lifetime limited products, such as foodstuffs that are contained within sealed packaging, components of which can be recycled. A typical inkjet formulation contains a dye or pigment plus a polymer binder to modify viscosity of the ink and impart adhesion to produce a robust image on the substrate surface. Design of the polymer binder can allow for enhancement of solubility and postdeposition modification; the latter often encompasses cross-linking of low molecular weight polymer chains either by the formation of covalent cross-links^52^ or supramolecular arrays.^53^

Base triggered depolymerisable poly­(olefin sulfone)­s (POSs) have been widely studied,^54–56^ with a notable body of work reported by Sasaki and co-workers^57,58^ who developed photoinduced depolymerisable POSs featuring pendant photobase generating groups. We have previously described a self-immolative chain-extended polyurethane (CEPU) which features a sulfonyl ethyl urethane (SEU) chain-extender which upon exposure to base undergoes rapid and efficient cleavage via a β-elimination process.^59^ Herein, we report the tailoring of physical and mechanical properties of debond-on-demand CPEU adhesives which feature the SEU chain-extender. Property modification was mediated by variations within the polyol backbone functionality to realize recyclable polymers for use in inkjet printing.

## 2 Results and Discussion

The everyday application of debond-on-demand adhesives is a significant goal, even more so when coupled with the need for such materials to be realized from commercially available materials, to possess high shear strength on a range of substrates, and rapidly debond from adhered surfaces.^1^ The use of 2,2′-sulfonyldiethanol (**1**) as a chain-extender in CEPUs has afforded rapid debond-on-demand adhesives upon the exposure of both NaOH and *tetra*-butylammonium fluoride (TBAF).^59^ To this end, the incorporation of this chain-extender into a series of CEPUs with a variety of functionalized polyol backbones has realized recyclable polymeric adhesives with tailorable adhesive capabilities for use in inkjet formulations.

### 2.1 Synthesis and Characterization of Model Small Molecule Analogues

We have previously shown^59^ that the SEU unit degrades via β-elimination upon exposure to base (NaOH and TBAF^60^) in both solution and solid state. To further explore the base degradation of the SEU unit, solution degradation of model small molecules (**2** and **3**) has been conducted with 1,8-diazabicyclo(5.4.0)­undec-7-ene (DBU), *N*,*N*-diisopropylethylamine (DIPEA), pyridine, and piperidine. The full synthesis and characterization of the small molecules can be found in the Supporting Information (Figures S1–S4), for degradation studies conducted via ^1^H NMR spectroscopy see Figures 1 and S5–S11.

**Figure 1:**
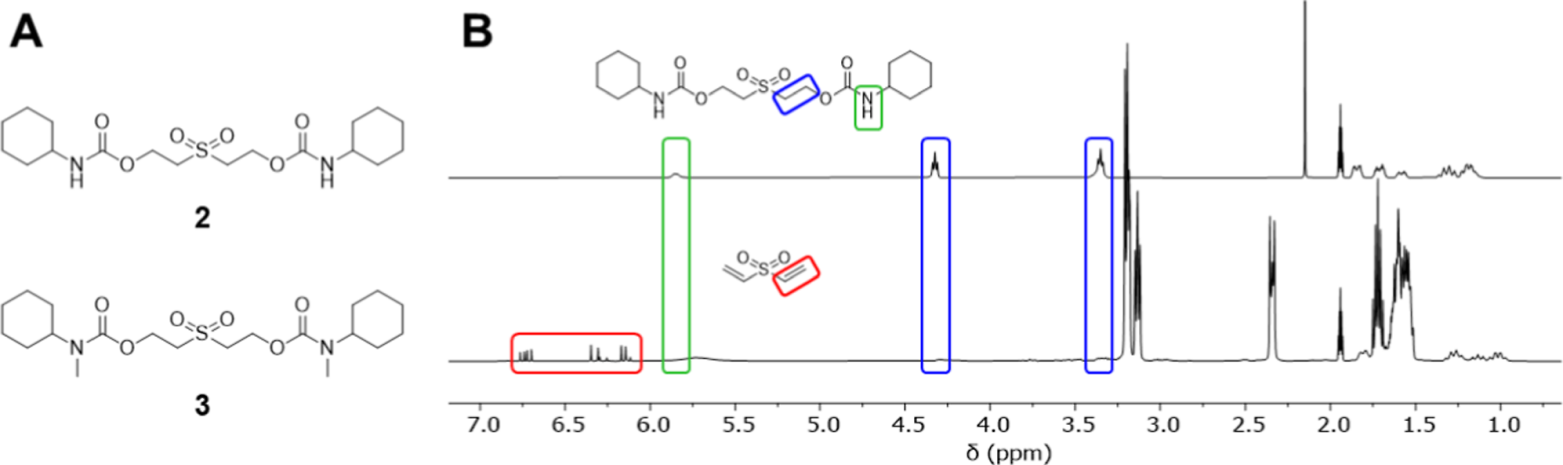
(A) The model urethanes **2** and **3** studied. (B) ^1^H NMR spectra of model urethane **2** in MeCN-*d*_3_ at 298 K, before exposure to DBU (top) and 5 min after exposure to DBU (bottom).

Upon exposure to DBU, ^1^H NMR spectroscopic analysis of model urethanes **2** and **3** revealed that degradation occurs via the proposed β-elimination/decarboxylation/amine release pathway and further confirmed the suitability of the bisurethane sulfone system as a degradable unit in CEPU backbones. Within 5 min of exposure complete degradation of the model urethanes was observed upon exposure to DBU, faster than the rate previously observed with NaOH_(aq)_ and TBAF.^59^ Unlike POS systems reported by Possanza Casey and Moore,^56^ exposure of **2** and **3** to pyridine did not result in degradation over a 24 h period at 25 °C, likely a result of the higher p*K*_b_ of pyridine when compared to the other bases tested. Exposing the model urethanes, **2** and **3**, to DIPEA and piperidine also did not result in the self-immolative degradation, most likely a result of the high p*K*_b_ of the bases. We have also previously shown that the exposure of urethane **2** to NaOD revealed that degradation also occurs via hydrolysis of the urethane linkage.^59^

### 2.2 Synthesis and Characterization of CEPUs

Having established the susceptibility of the SEU unit to degradation via treatment with a range of bases, a library of potential adhesives were synthesized via a one-pot two-step synthesis that has previously been used to generate analogous degradable CEPU adhesives,^11,40,59^ see Scheme 1. Variations within the polyol backbone functionality will provide tailoring of physical and mechanical properties and enable a rational improvement to **CEPU1** of which we have previously reported.^59^ The backbone polyol functionalities include alkyl (**CEPU1**, Krasol HLBH-P 2000), ether (**CEPU2**, poly­(ethylene glycol), and **CEPU3** poly­(tetrahydrofuran) (PTHF)), and ester functionalities (**CEPU4**, poly­(caprolactone) (PCL), and **CEPU5**, Stepanpol PC-205P-30)), and the synthetic protocols and corresponding characterization of these materials are included in the Supporting Information (see Figures S12–S34). These CEPUs synthesized maintained their structural and molecular weight characteristics in solution over the duration of this study (>1 year). Composition, molecular weights, and key thermal transitions of the CEPUs have been summarized in Table 1.

**Scheme 1:**
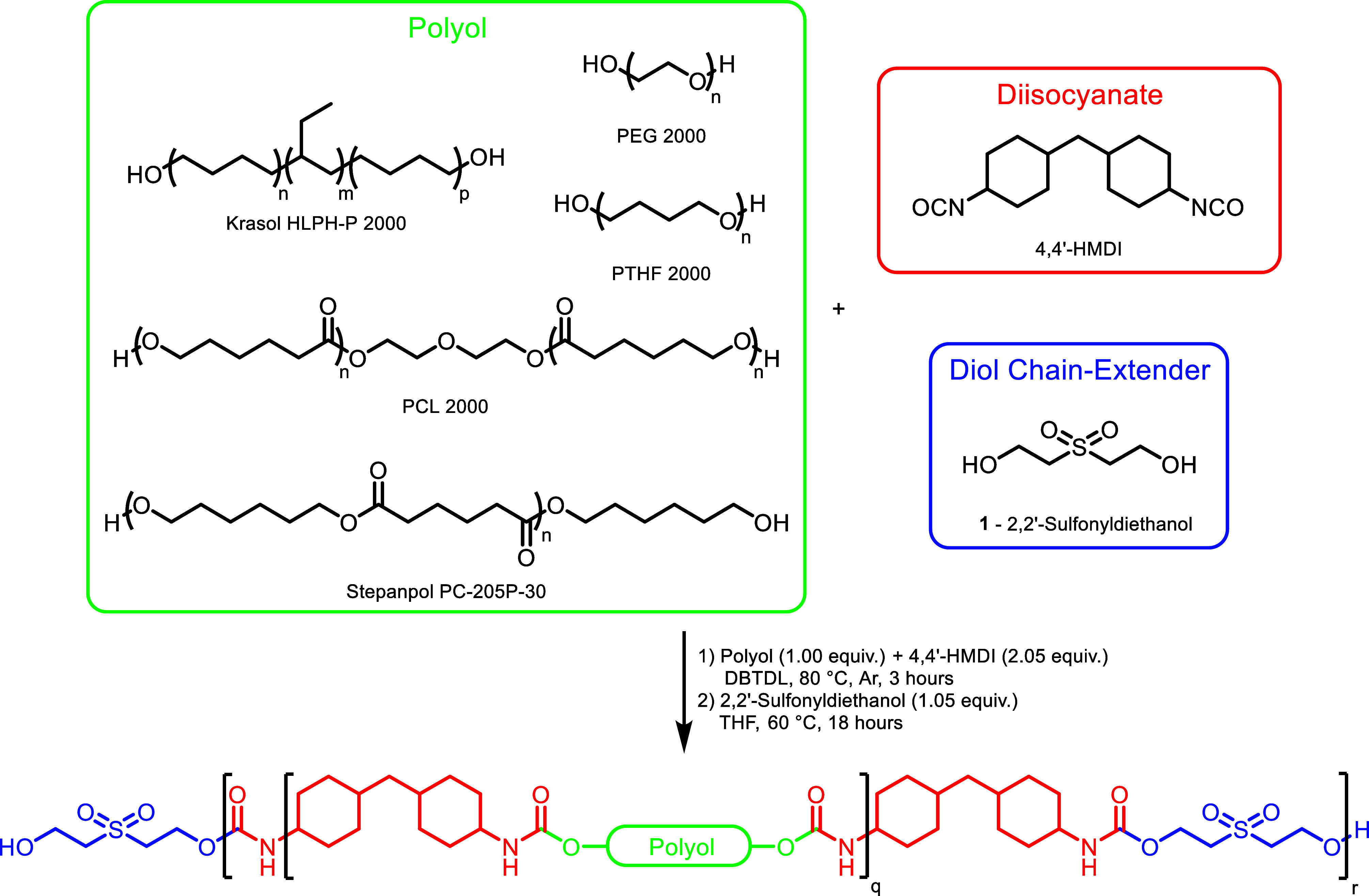
General Synthetic Protocol to Afford **CEPU1-CEPU5** Comprised of Different Polyols

**Table 1:** Chemical Composition of **CEPU1**-**CEPU5** Containing Different Polyols (Yields are Shown in Brackets), GPC Molecular Weight and Dispersity Data for **CEPU1**-**CEPU5** (the Error Shown is the Standard Deviation between the Three Repeats of Each Sample), and Thermal Properties of **CEPU1**-**CEPU5**

CEPU	polyol	polyol functionality	*M*_n_ (g mol^–1^)	*M*_w_ (g mol^–1^)	*D̵*	*T*_g_ (°C)^b^	*T*_m_ (°C)^a^	*T*_m_ (°C)^b^	*T*cc/*T*c (°C)
**CEPU1** (92%)	Krasol HLBH-P 2000	alkyl	44,700 ± 200	140,400 ± 700	3.14	–47.1	50.1		
**CEPU2** (70%)	PEG 2000	ether	22,200 ± 100	62,600 ± 1300	2.82	–51.8		25.1	–34.3^c^; −4.9^e^
**CEPU3** (74%)	PTHF 2000	ether	68,400 ± 900	167,800 ± 300	2.45	–74.0			
**CEPU4** (82%)	PCL 2000	ester	28,100 ± 200	124,300 ± 1600	4.42	–47.4	25.4; 39.6		
**CEPU5** (88%)	Stepanpol PC-205P-30	ester	23,600 ± 100	45,700 ± 300	1.94		43.5	48.1	32.7^d^; 32.8^e^

a First heating run 10 °C min^–1^.

b Second heating run 10 °C min^–1^.

c *T*_cc_ from second heating run 10 °C min^–1^.

d *T*_c_ from first cooling run 10 °C min^–1^.

e *T*_c_ from second cooling run 10 °C min^–1^.

^1^H NMR spectroscopic analysis of the CEPUs revealed a 1:1 ratio between the resonances associated with the prepolymer urethanes and the chain-extender urethanes, which is consistent with the feed ratios (see the NMR spectroscopic data in the Supporting Information, Figures S12–S19). ^13^C NMR spectroscopy also confirmed the formation of the urethane linkages with prepolymer urethane linkages observed at ca. 156 ppm. Characteristic absorbance bands from urethane units were observed in the FTIR spectra corresponding to the N–H and C=O stretches, ca. 3300 cm^–1^ and ca. 1700 cm^–1^, respectively. GPC analysis of the CEPUs was employed to determine their molecular weights (see Table 1 and Figure S20–S24), all CEPUs exhibited broad monomodal distributions in molecular weight with values for *M*_n_ > 22,000 g mol^–1^. Thermogravimetric analysis (TGA) was employed to determine the maximum processing temperature of the CEPUs, **CEPU2** exhibited the lowest temperature for the onset of degradation at 215.8 °C and all CEPUs degraded fully once the environment reached 475 °C (see Figure S25–S29). The thermal transitions of the CEPUs were investigated through differential scanning calorimetry (DSC), see Table 1. **CEPU1**-**CEPU4** all exhibited characteristic glass transitions (*T*_g_) corresponding to their polyol backbones.^11,23,61,62^ In the first heating and cooling cycles of **CEPU2**, defined thermal transitions were not observed, however, in the second heating cycle a well-defined *T*_g_, cold crystallization (*T*_cc_), and melt transition (*T*_m_) were evident, with a broad crystallization (*T*_c_) also present in the second cooling cycle. Further heating and cooling cycles only reveal the presence of *T*_m_ and *T*_c_ transitions, respectively, indicating an initial amorphous structure which rearranges to produce a highly crystalline structure. **CEPU4** and **CEPU5** exhibit well-defined *T*_m_’s in the heating cycles corresponding to the melt of the polyester backbones. The recrystallization of the PCL backbone of **CEPU4** was slower than the time scale of the experiment and therefore is not observed in the cooling cycle, whereas the *T*_c_ of the Stepanpol backbone of **CEPU5** was evident.

Variable temperature (VT) small-angle X-ray scattering (SAXS) and wide-angle X-ray scattering (WAXS) were carried out on thin film samples of the CEPUs to investigate the effect that the polyol composition has on the morphology of the system and the resultant temperature susceptibility, see Figure 2, Supporting Information Figures S35–S44 and Table S1. The variations in the polyol functionality of **CEPU1**-**CEPU5** results in changes within the room temperature SAXS diffraction data, **CEPU2** featuring the PEG polyol was observed to be amorphous without defined Bragg peaks in the SAXS, **CEPU3** featuring the PTHF polyol exhibited a weakly diffracting Bragg peak with a *q*_max_ and *d*-spacing of 0.44 nm^–1^ and 14.3 nm, respectively. Whereas, in contrast, the ester polyols (**CEPU4** and **CEPU5**) exhibit defined Bragg peaks resulting from defined phase separation within the bulk of the polymer, with *q*_max_’s *ca*. 0.37 nm^–1^ corresponding to *d*-spacings of 17.0 nm. The above dimensions vary from **CEPU1** that features an alkyl polyol which we have previously reported to have a *q*_max_ and *d*-spacing of 0.7 nm^–1^ and 9.0 nm, respectively.^59^ Room temperature WAXS of the CEPUs (Figure 2B) provides insight into the ordering of the hard-segment domains. All CEPUs feature a broad diffraction peak with maxima between 13.4 and 14.5 nm^–1^ corresponding to spacings of 0.43–0.47 nm and can be attributed to the hydrogen bonding urethane residues.^63^ The crystalline ester polyol backbones of **CEPU4** and **CEPU5** provide further well-resolved peaks at 15.4 nm^–1^ and *ca*. 17.2 nm^–1^ relating to length scales of 0.41 nm and *ca*. 0.37 nm, respectively. The VT-SAXS and VT-WAXS data of **CEPU2** and **CEPU3** do not reveal any significant changes in the phase separation or hard domain ordering over the temperature ranges monitored. However, the melting of the crystalline domains of the crystalline ester polyol CEPUs was observed with loss of the diffraction peaks at 45 °C (**CEPU4**) and 60 °C (**CEPU5**), respectively, with the onset of the change in the diffraction patterns coinciding with the *T*_m_’s observed in the DSC plots. Notably, the recrystallization of the polyol backbone of **CEPU5** was observed upon cooling below 30 °C, consistent with its *T*_c_.

**Figure 2:**
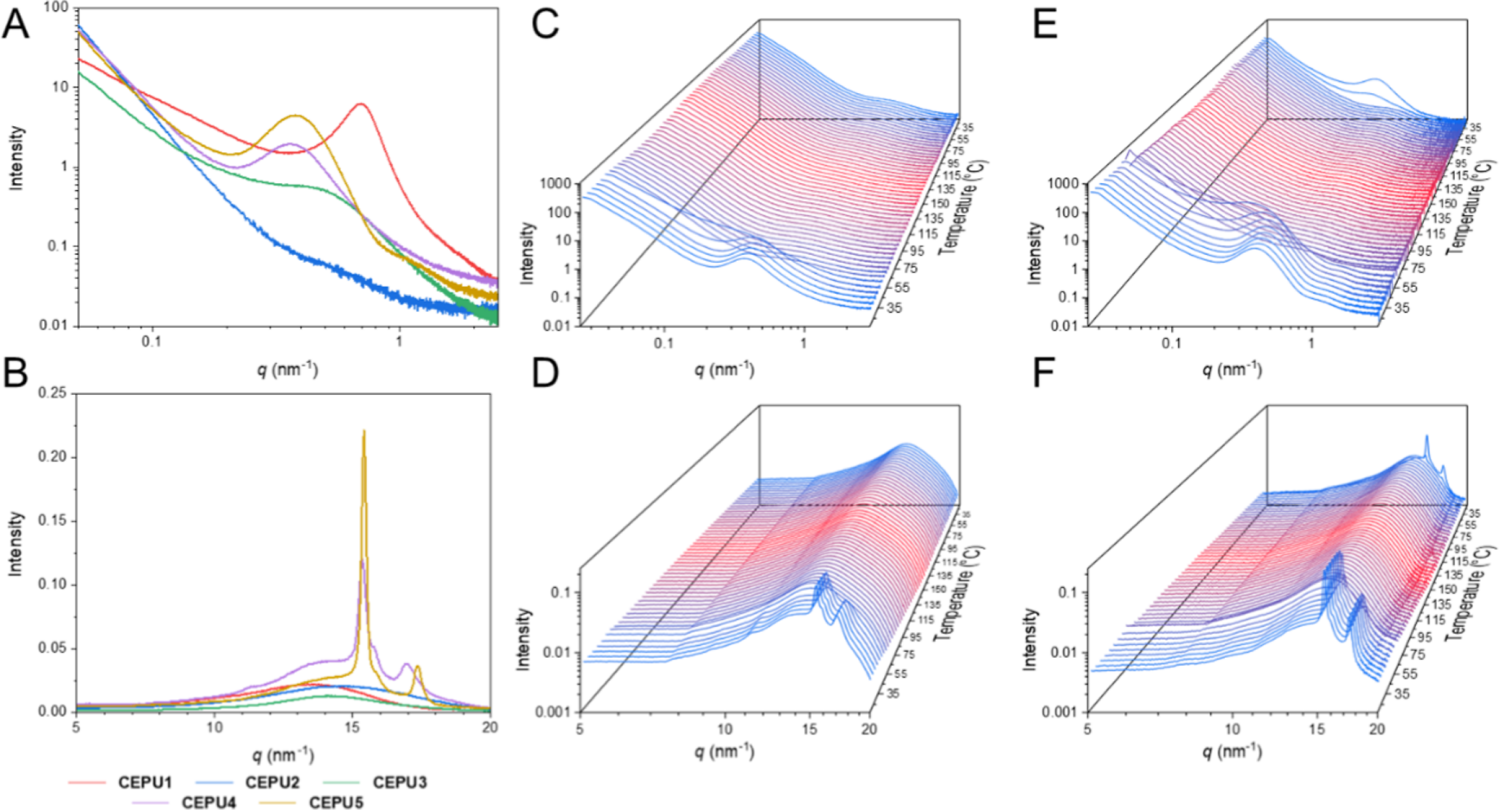
(A) SAXS and (B) WAXS intensity profiles of **CEPU1**-**CEPU5** at 20 °C. VT-SAXS and VT-WAXS of **CEPU4** ((C,D), respectively) and **CEPU5** ((E,F), respectively) recorded at 5 °C intervals from 20 to 200 °C at a heating and cooling rate of 10 °C min^–1^.

Rheological analysis of the CEPUs was carried out to determine the viscoelastic transition temperatures to inform the lowest possible temperature at which the CEPUs can be hot melt processed (see Figures 3 and S45–S49).^64^ **CEPU1** and **CEPU3** both exhibit high temperature viscoelastic transitions, at 145.8 and 143.2 °C, respectively. In comparison **CEPU2**, **CEPU4**, and **CEPU5** exhibit lower temperature viscoelastic transitions at 32.4, 56.2, and 52.8 °C, respectively. **CEPU2**, **CEPU4**, and **CEPU5** all exhibit viscoelastic transitions below 60 °C which also correlate with the *T*_m_ values determined by DSC analysis and the changes in phase separation and crystallinity observed in the VT-SAXS and VT-WAXS studies. **CEPU5** also exhibits a rapid loss in storage modulus (G′) of *ca*. 2 × 10^6^ Pa over only 2.6 °C, corresponding to the melt of the ordered crystalline polyester backbone. **CEPU2** also exhibited a rapid loss in G′ whereas the decrease in storage modulus for the other CEPUs occurred over a broader temperature range (*ca*. 1.5 × 10^6^ Pa over 6.5 °C).

**Figure 3:**
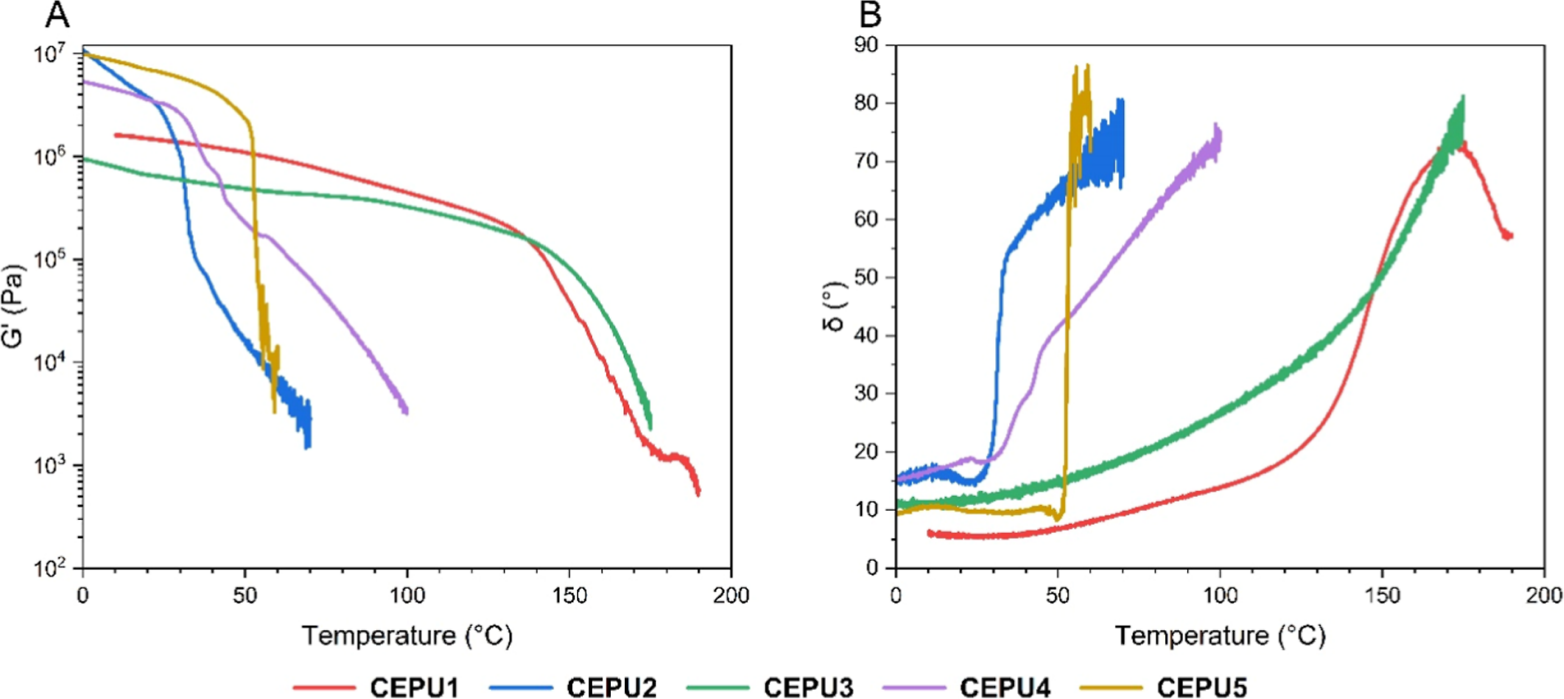
Temperature sweep analysis of **CEPU1**-**CEPU5** over a temperature regime of 0 to 190 °C, using a normal force of 1 N and a frequency of 1 Hz. (A) storage modulus (*G*′) versus temperature, (B) phase shift (δ) versus temperature.

The mechanical properties of the CEPUs were investigated via stress–strain tensile testing at 10 mm min^–1^, with each sample repeated in triplicate, see Figure 4 and Supporting Information Figures S50–S54. **CEPU3**, which features the PTHF polyol exhibits extraordinary values for ultimate tensile strength (UTS), elongation at break (EB), and modulus of toughness (MoT) of 21.52 ± 2.25 MPa, 19.50 ± 1.75 ε, and 234.75 ± 37.20 MJm^–3^, respectively. However, the recorded values for UTS, EB, and MoT for **CEPU3** are not a true representation of the polymer properties as material failure was not obtained before exceeding the limitations of the instrument used in this study and therefore can potentially be significantly higher. **CEPU5** exhibited the highest Young’s modulus (YM) of 160.15 ± 13.13 MPa, with the YM increasing through the CEPU series with increasing functionality of the polyol backbone used (i.e., alkyl < ether < ester).

**Figure 4:**
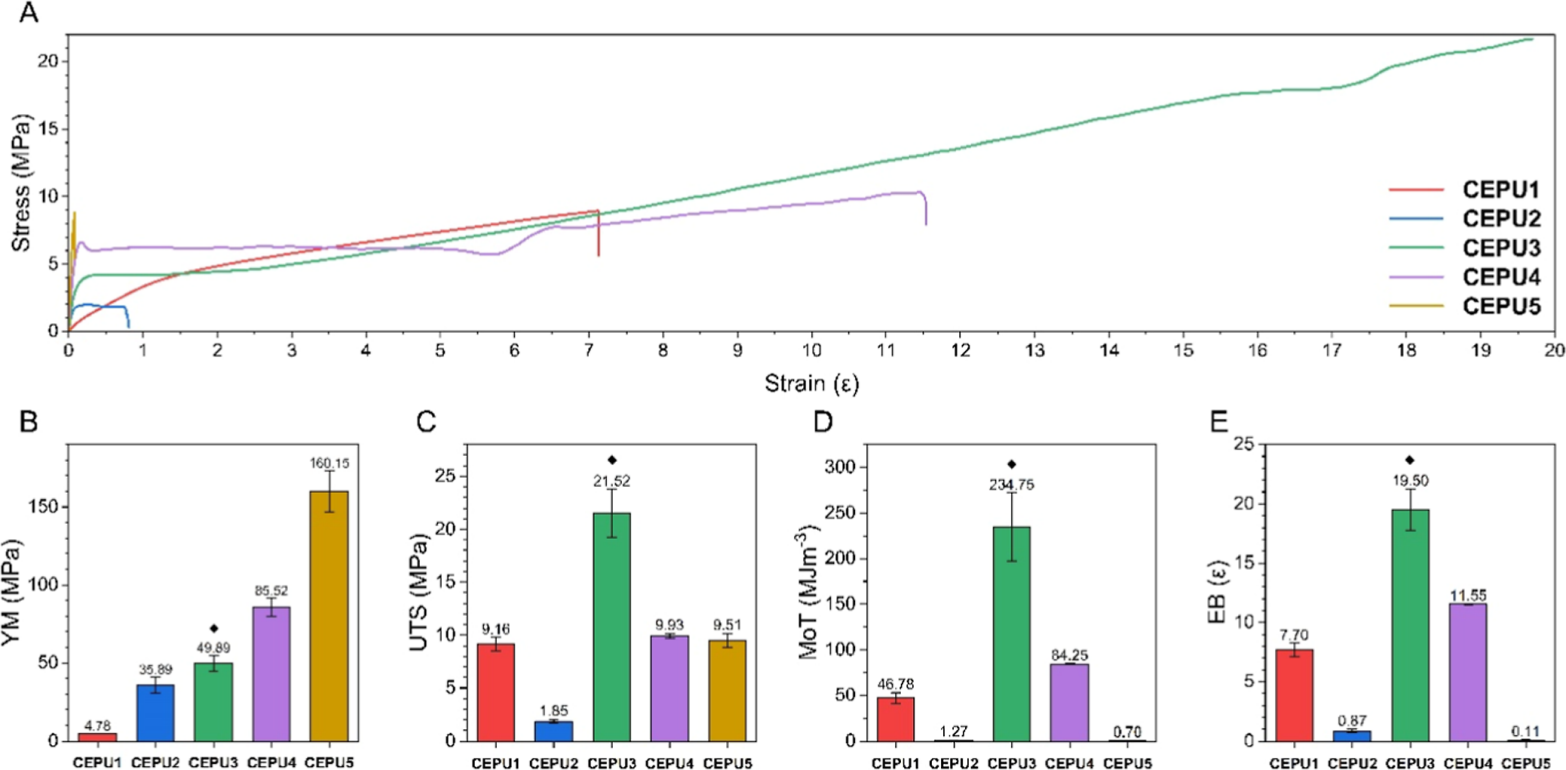
(A) Representative stress–strain curves for **CEPU1**-**CEPU5**. Comparison of (B) Young’s modulus (YM), (C) ultimate tensile strength (UTS), (D) modulus of toughness (MoT), and (E) elongation at break (EB). The error shown is the standard deviation between the three repeats for each sample. ◆ Value obtained at instrument limit.

### 2.3 CEPU Degradation Studies

The base initiated depolymerization of the CEPUs was first probed by solution-state NMR studies by the addition of excess base, either NaOD, TBAF, or DBU, to a sample of CEPU in THF-*d*_8_ (5:1 molar equiv of base to degradable unit). The reaction was monitored by ^1^H and ^13^C NMR spectroscopy, see Figures S55–S67. For all CEPUs, within 30 min of exposure to the base the chain-extender urethane and methylene resonances rapidly diminish and the vinylic protons of divinyl sulfone are evident. The urethane resonance, ca. 156.1 ppm, associated with the chain-extender was no longer evident in the ^13^C NMR spectra postdegradation. GPC solution state degradation studies of the CEPUS were conducted via the addition of TBAF (see Figure 5 and Supporting Information Figures S68–S71). All of the CEPUs observed rapid loss in *M*_n_ and *M*_w_ after only 30 min post exposure to TBAF (**CEPU3** *M*_w_ ca. 150,000 g mol^–1^ and 88%), extension of the exposure time to 24 and 48 h only provided minimal narrowing of the molecular weights exemplifying the rapid degradation of the SEU moiety when in solution (for molecular weight data see Table S2).

**Figure 5:**
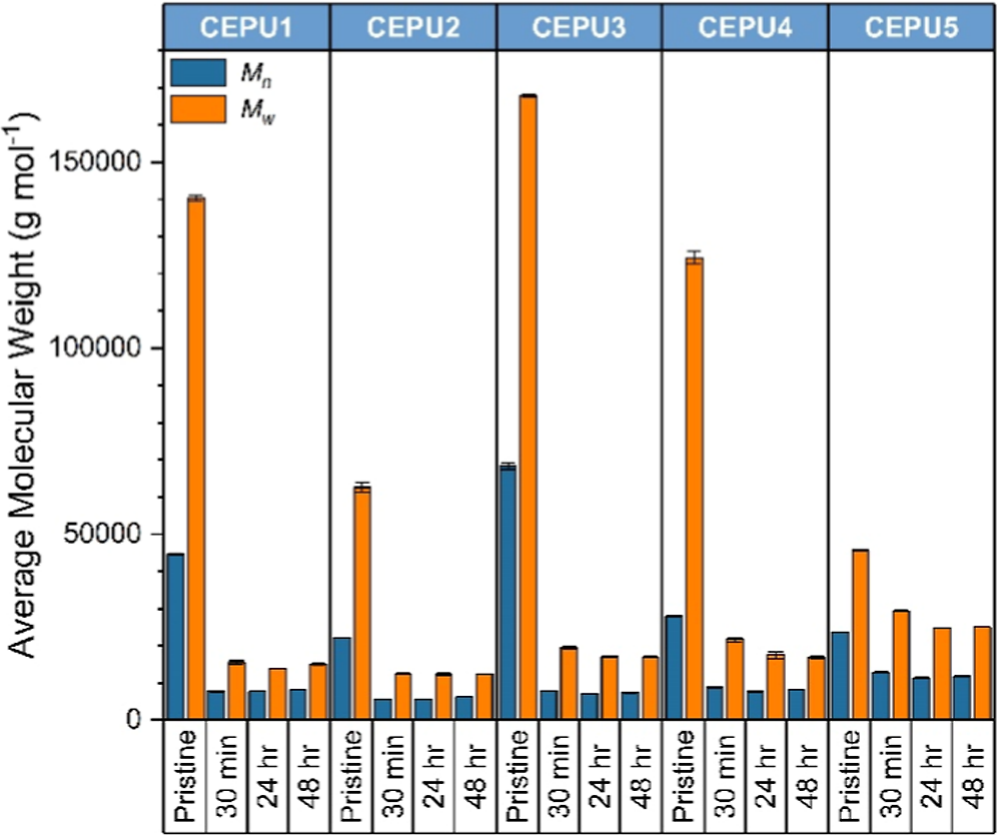
*M*_n_ (blue) and *M*_w_ (orange) of **CEPU1**-**CEPU5** as pristine samples and 30 min, 24 h, and 48 h post addition of TBAF acquired from a THF GPC; the recorded are averages of three separate samples of each CEPU. The error shown is the standard deviation between the three repeats of each sample.

### 2.4 Adhesion Studies

The hot melt adhesive capabilities of the CEPUs were investigated via lap-shear adhesion tests on several different surfaces to mimic different types of packaging, including aluminum, glass, wood, high density poly­(ethylene) (HDPE), poly­(propylene) (PP), Nylon, polyethylene terephthalate (PET), and polyvinyl chloride (PVC), see Figure 6 and Table 2. The samples were adhered at temperatures above their observed viscoelastic transition at 150 °C (**CEPU1** and **CEPU3**, except for HDPE and PVC substrates which were adhered at 120 °C), 60 °C (**CEPU4** and **CEPU5**), or 45 °C (**CEPU2**) for 30 min, with each sample being tested in triplicate. **CEPU1** and **CEPU3** were unsuitable for adhesion to PET because of the low melting temperature of substrates in relation to the high adhesion temperature required for the CEPUs. Adhesion of **CEPU1** and **CEPU3** to HDPE and PVC required a lower adhesion temperature of 120 °C to ensure substrate integrity.

**Figure 6:**
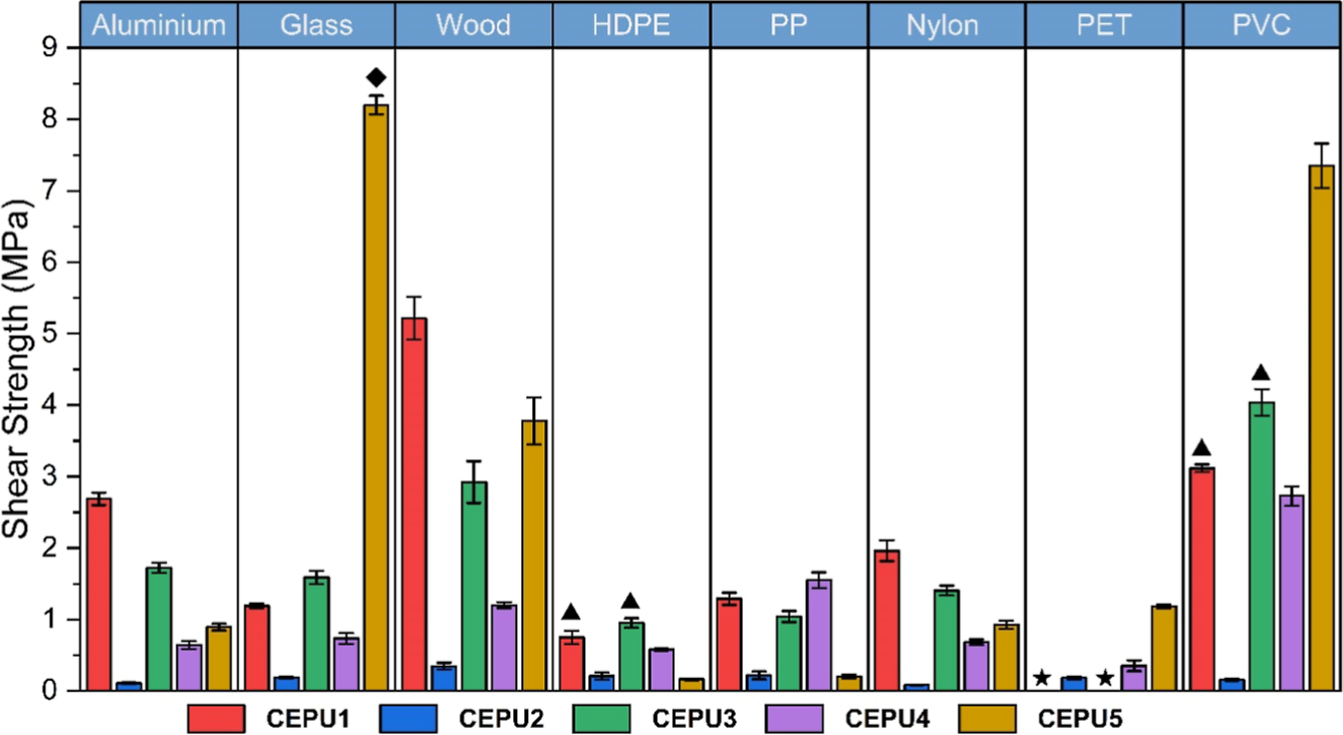
Shear strength of **CEPU1**-**CEPU5** on aluminum, glass, wood, high density poly­(ethylene) (HDPE), poly­(propylene) (PP), Nylon, polyethylene terephthalate (PET), and polyvinyl chloride (PVC). The error shown is the standard deviation between the three repeats of each sample. ◆ Substrate failure was achieved before adhesive failure. ▲ Adhesion occurred at 120 °C instead of at 150 °C used for other substrates. ★ Shear strength could not be obtained resulting from low substrate melting temperatures.

**Table 2:** Shear Strength of **CEPU1**-**CEPU5** on Aluminum, Glass, Wood, High Density poly­(ethylene) (HDPE), poly­(propylene) (PP), Nylon, Polyethylene Terephthalate (PET), and Polyvinyl Chloride (PVC) (the Error Shown is the Standard Deviation Between the Three Repeats of Each Sample)

CEPU adhesive	aluminum (MPa)	glass (MPa)	wood (MPa)	HDPE (MPa)	PP (MPa)	nylon (MPa)	PET (MPa)	PVC (MPa)
**CEPU1**	2.69 ± 0.19	1.19 ± 0.03	5.22 ± 0.30	0.75 ± 0.09^b^	1.29 ± 0.09	1.96 ± 0.15	N/A^c^	3.12 ± 0.06^b^
**CEPU2**	0.11 ± 0.01	0.19 ± 0.01	0.35 ± 0.05	0.21 ± 0.05	0.22 ± 0.06	0.08 ± 0.004	0.18 ± 0.02	0.15 ± 0.02
**CEPU3**	1.72 ± 0.07	1.59 ± 0.09	2.92 ± 0.29	0.95 ± 0.06^b^	1.04 ± 0.08	1.41 ± 0.07	N/A^c^	4.04 ± 0.19^b^
**CEPU4**	0.64 ± 0.05	0.73 ± 0.08	1.20 ± 0.04	0.58 ± 0.01	1.55 ± 0.01	0.68 ± 0.04	0.35 ± 0.07	2.73 ± 0.14
**CEPU5**	0.89 ± 0.05	8.20 ± 0.13^a^	3.78 ± 0.33	0.16 ± 0.01	0.20 ± 0.03	0.92 ± 0.06	1.18 ± 0.03	7.35 ± 0.31

a Substrate failure was achieved before adhesive failure.

b Adhesion occurred at 120 °C instead of at 150 °C used for other substrates.

c Shear strength could not be obtained resulting from low substrate melting temperatures.

Substrate fracture of glass was observed prior to reaching adhesive failure with the use of **CEPU5** at 8.20 MPa. **CEPU1** and **CEPU2** both achieved their highest shear strength when adhered to wood at 5.22 ± 0.30 MPa and 0.35 ± 0.05 MPa, respectively. **CEPU3** and **CEPU4** exhibited their highest shear strength when adhered to PVC (4.04 ± 0.19 MPa and 2.73 ± 0.14 MPa, respectively). The CEPUs are shown to adhere to both high energy surfaces, such as aluminum and glass, and low energy surfaces, such as HDPE and PP. The shear strength of **CEPU5** when adhered to glass (8.20 MPa) was higher than several published debond-on-demand adhesives, as illustrated in Supporting Information Figure S72.^7,12–14,58,65–67^

The debond-on-demand properties of the CEPUs were tested upon exposure to 40 wt % NaOH_(aq)_, 1 M TBAF_(aq)_, and 1 M DBU_(aq)_ solutions at room temperature, with the best performing adhesive for each substrate, the porous nature of wood excluded it from testing, see Figure 7 and Table S3. We have previously shown in the case of **CEPU1** ^59^ that minimal changes in shear strength are achieved with extending the degradation time from 30 min to 24 h, therefore, all samples were exposed to base for 30 min; the debond-on-demand procedure for the adhered samples is outlined in the Supporting Information. Control samples were submerged in deionized water for 30 min, with all of the adhered surfaces presenting shear strengths within the error of the pristine sample. **CEPU5** adhered to glass exhibited the greatest losses in shear strength of up to 92% when exposed to 40 wt % NaOH_(aq)_, from 8.20 to 0.62 MPa, followed by **CEPU4** adhered to PP which observed losses in shear strength of 77% upon exposure to 1 M TBAF_(aq)_. Debonding of **CEPU5** from PET by treatment with NaOH revealed the lowest loss in shear strength of only 33%, however, the use of TBAF or DBU increased the debonding with losses of 55% and 41%, respectively. To place these results into context, the fluoride responsive self-immolative thermosets by Kim and co-workers required extended exposure times, >3 h, to 1 M CsF to achieve similar losses in shear strength when adhered to glass,^14^ and our previous CEPUs featuring chain-extender **1** only observed up to 60% reduction in shear strength upon exposure to 1 M TBAF for 30 min^59^

**Figure 7:**
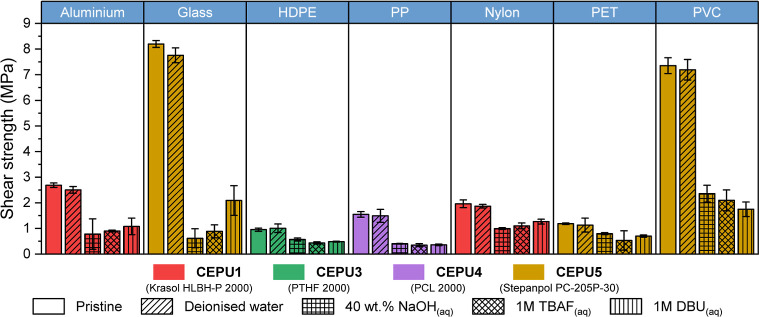
Shear strength of the best CEPU adhesive (and their key backbone composition) on aluminum, glass, high density poly­(ethylene) (HDPE), poly­(propylene) (PP), Nylon, polyethylene terephthalate (PET), and polyvinyl chloride (PVC) as the pristine sample and after exposure to deionized water, 40 wt % NaOH_(aq)_, 1 M TBAF_(aq)_, and 1 M DBU_(aq)_. The error shown is the standard deviation between the three repeats of each sample.

### 2.5 Solubility of CEPUs

The solubility of the CEPUs in solvents compatible with inkjet printing is critical to generate jettable formulations. The solubility of the CEPUs was therefore investigated by generating solvation spheres using Hansen Solubility Parameter (HSP) analysis. A range of 27 polar and apolar organic solvents were chosen to generate the solubility characteristics of the CEPUs at room temperature, see Supporting Information Table S4 and Figures S73–S77. Across the range of solvents tested, **CEPU2** proved to be the most soluble CEPU, generating the largest solubility sphere, whereas **CEPU1** was the least soluble CEPU. **CEPU3** was found to be soluble in more solvents than **CEPU4** and **CEPU5**, however, **CEPU3** exhibited a smaller solubility sphere based upon the solvents it is soluble in. Unlike the other CEPUs, the PEG backbone in **CEPU2** enhanced this polymers’ solubility in deionized water.

### 2.6 Contact Angles and Surface Free Energies

Films of the CEPUs were produced by casting, drawing down, and evaporating a 5 wt % solution of CEPU in THF (**CEPU1**) or butanone (**CEPU2**-**CEPU5**) using a 24 μm K-bar under ambient conditions. Films of CEPUs were produced on aluminum, glass, HDPE, PP, Nylon, PET, and PVC, all precleaned with an isopropanol soaked paper towel; wood was excluded because of its porous nature. The surface free energy (SFE) of each CEPU coated surface was determined via contact angle measurements, with the contact angles of both water and diiodomethane being measured on the coated and uncoated surfaces, see Supporting Information Figures S78–S80 and Table S5. The polyol functionality used within the CEPUs significantly influenced the wetting characteristics of the surfaces, for instance, the polar polyols of **CEPU2**-**CEPU5** increased the wetting capability of low SFE surfaces such as HDPE and PP by introducing functional units with the ability to form supramolecular interactions, corresponding to lower contact angles and increased SFE values. CEPU coating of Nylon, PET, and PVC varies the SFE, with both increases and decreases observed, this can be attributed to how the CEPUs interact differently with the functional units on the polymer surfaces (amide, ester, and chloride, respectively), for example through supramolecular interactions.

### 2.7 Inkjet Printing Studies

The selective depolymerization of these polymers make them interesting as potential candidates for debondable binders for inkjet inks. This would enable the removal of ink from substrates before they enter the recycling process, preventing ink contaminants decreasing the quality of the recycled material. High-throughput continuous inkjet (CIJ) printers require ink droplets to possess a charge so that they can be deflected toward the substrate via application of an electric field to effect selective deposition. Sometimes the conductivity of the dye is sufficient to allow this; if not conductivity salts can be added to the ink. To this end the SEU unit was probed for its stability in the presence of common salts used in inkjet formulations using model urethane **2**. Model urethane **2** was exposed to excess *tetra*-butylammonium hexafluorophosphate (TBAPF_6_) and *tetra*-butylammonium nitrate (TBAN) and monitored via ^1^H NMR spectroscopic analysis, see Figures S81 and S82. Over the course of 12 months, degradation of the model urethanes was not observed when exposed to either TBAPF_6_ or TBAN, thus indicating their potential use within the inkjet formulation.

The viscosity of inkjet formulations is important as it influences droplet formation and printing ability, therefore the wt % of CEPUs used in the prototype formulations were varied to fit the viscosity within the suitable range (3–7 cP) for the CIJ printer setup used in this study (see Table 3). **CEPU2**-**CEPU5** are soluble in butanone, which is commonly used in industrial inkjet formulations, **CEPU1** is insoluble in butanone and therefore was formulated in THF. Conductivity of the ink formulations was provided from the addition of TBAPF_6_ (0.25 wt %) and, with the exception of **CEPU1**, fall within the range required for CIJ printing (400–1500 μS cm^–1^). Oil blue 613 was used as the dye at 1 wt % for the formulations to help visualize the deposition onto the substrates tested.

**Table 3:** Composition, Viscosity, Conductivity, and Density of Inkjet Formulations

CEPU adhesive	content of CEPU (wt %)	content of TBAPF_6_ (wt %)	content of dye (wt %)	viscosity (cP)^a^	conductivity (μS cm^–1^)^a^	density (g cm^–3^)^a^
**CEPU1**	5	0.25	1.0	6.52	18.7	0.888
**CEPU2**	7.5	0.25	1.0	6.82	447.1	0.819
**CEPU3**	5	0.25	1.0	5.69	466.6	0.813
**CEPU4**	7.5	0.25	1.0	3.12	413.8	0.822
**CEPU5**	10	0.25	1.0	3.99	420.5	0.827

a Values were recorded at 25 °C.

The linear viscoelastic properties of the inkjet formulations were investigated using a Piezo Axial Vibrator (PAV) rheometer between 10 and 10,000 Hz at 25 °C, see Figure S83. **CEPU1** was not tested as the formulation is incompatible with CIJ printing. Formulations of **CEPU2** and **CEPU3**, which comprise of ether polyols, both exhibit significant elasticity at high frequency. Conversely, the lower viscosity formulations containing the more rigid and crystalline ester backbones of **CEPU4** and **CEPU5** do not exhibit elasticity within the frequency range tested. Droplet breakup is important to develop well-resolved print images and thus the formulations of **CEPU2**-**CEPU5** were studied for their breakup of the jet stream. Formulated **CEPU2** and **CEPU3** both reveal longer lasting thin filaments connecting the droplets to delay break-off during drop formation as the polymer chains unravel in this extensional fluid behavior within the elasto-capillary balance. This can be attributed to the high molecular weight of the polymers and the elasticity detected by the PAV rheometer in the ink samples that indicates longer relaxation times, see Figure S84A,B. The formulations featuring **CEPU4** and **CEPU5** both exhibit good breakup of the jet stream plus small tails on the droplets that recombine to the main droplet further down the stream avoiding the formation of satellite drops on account of their ridged backbone, see Figure S84C,D. No elasticity was measured by the PAV technique for these fluids. The CEPU formulations were subsequently deposited from a CIJ printhead onto a variety of substrates using a Domino Ax350i CIJ printer with an i-pulse print head. The poor droplet breakup of the **CEPU2** and **CEPU3** formulations produced poor quality prints on all substrates, see Figures S85 and S86. Further optimization of the formulations for **CEPU1**-**CEPU3** are thus required in order to achieve high resolution printed images of these materials via inkjet means. However, formulations of **CEPU4** and **CEPU5** produced clear well-defined images without any apparent satellite drops or misplaced drops, see Figures 8A and S87.

**Figure 8:**
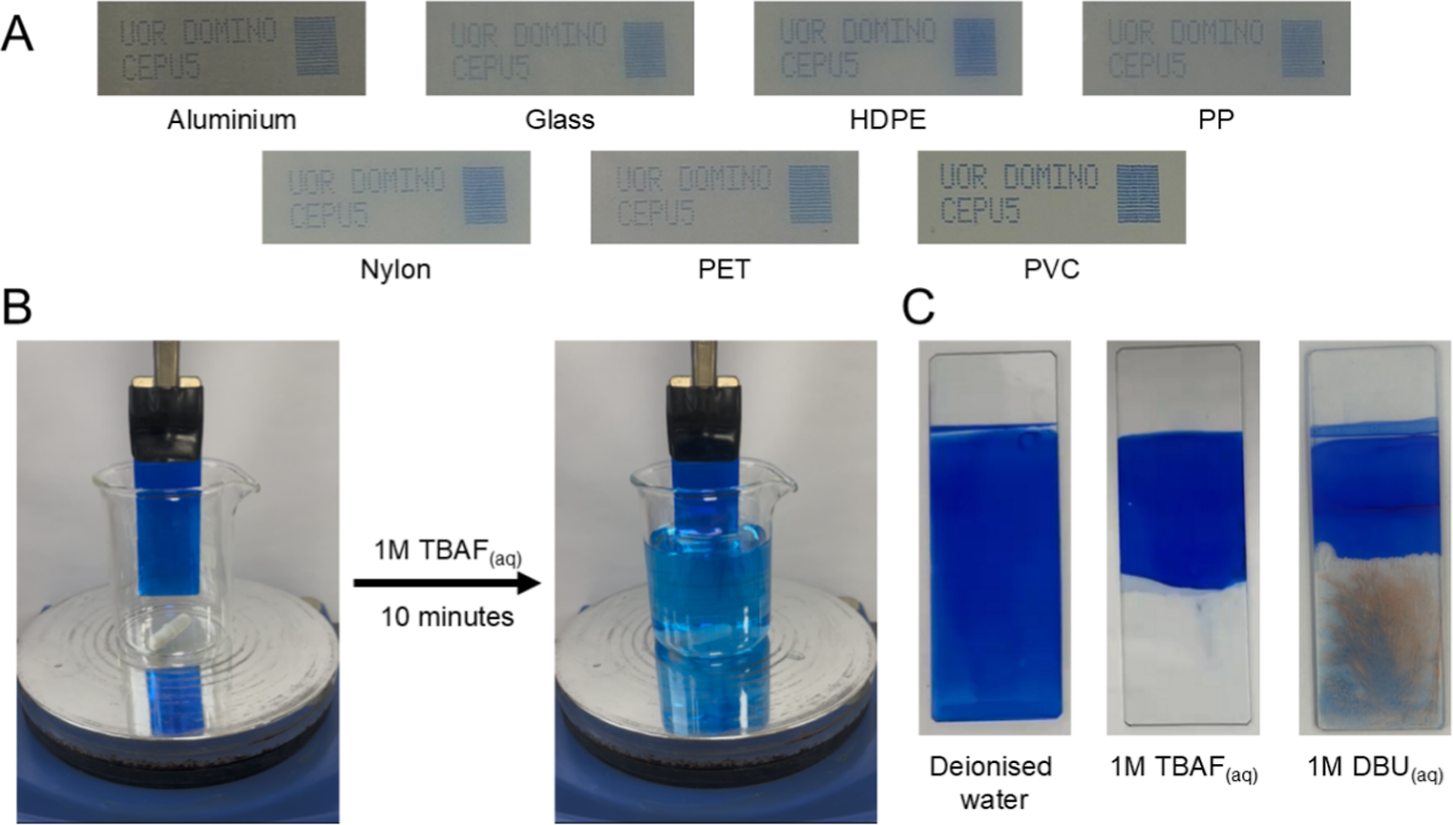
(A) CIJ deposition of **CEPU5** formulation onto aluminum, glass, high density poly­(ethylene) (HDPE), poly­(propylene) (PP), Nylon, polyethylene terephthalate (PET), and polyvinyl chloride (PVC). (B) Debonding of **CEPU3** inkjet formulation from a glass slide using 1 M TBAF_(aq)_ over 10 min. (C) Glass slides coated in **CEPU3** formulation after 10 min in deionized water, 1 M TBAF_(aq)_, and 1 M DBU_(aq)_.

The adhesion of the formulations on the substrates was subsequently evaluated using peel tests. Adhesive tape (810 grade) was utilized and the amount of material removed from the surface was graded by an arbitrary value between 1 and 5 (where 5 indicated no removal of the print (excellent adhesion) and 1 indicated the complete removal of the print (very poor adhesion)). All of the deposited CEPUs exhibited poor or very poor adhesion to aluminum, HDPE, and PP, attributed to the low surface energies of the surfaces and the presence of an oxide layer on the aluminum surface. However, all the CEPUs demonstrated excellent adhesion on all of the other surfaces tested: glass, Nylon, PET, and PVC, see Supporting Information Table S6.

To demonstrate the ability to remove the inkjet formulations from substrates, glass slides were coated with the formulations using a 24 μm K-bar and subsequently submerged into base solutions at room temperature for 10 min, see Figure 8B,C plus Supporting Information Figures S88–S94. Submerging the coated substrates in deionized water did not result in debonding of the formulation or leaching of the dye for **CEPU1** and **CEPU3**-**CEPU5**, the water-soluble nature of **CEPU2** allowed for the partial leaching of the dye and weakening of adhesion to the glass substrate. Debonding initiated with 1 M TBAF_(aq)_ resulted in the rapid removal of the formulation over 10 min, exemplified in Figure 8B with **CEPU3**. The exposure of the coated substrates to 40 wt % NaOH_(aq)_ did not result in the removal of the formulation, instead the exposure to the base weakened the formulations adhesion to the glass substrate allowing for its removal using a cotton swab. Treatment with 1 M DBU_(aq)_ resulted in the partial debonding of the formulation from the glass substrate, any residual polymer on the surface could be removed easily with a cotton swab and degradation of the dye was observed through the loss of color, see Figures 8C and S90 and S94.

### 2.8 Recycling

To aid with the generation of a circular plastic economy, ideally all components of packaging should be recyclable, therefore the previously synthesized adhesive **CEPU5** (possessing the Stepanpol PC-205P-30 backbone) was intentionally degraded and recycled into a new recycled CEPU (**rCEPU5**), as shown in Scheme 2. Briefly, the pristine **CEPU5** was dissolved in THF and exposed to DBU for 1 h, then the polymer byproduct of this process was purified by repeated precipitations into methanol to afford an amino terminated prepolymer. The prepolymer was subsequently dissolved in dry THF and reacted with 4,4′-HMDI and 2,2′-sulfonyldiethanol to afford the **rCEPU5**. The synthetic protocol and corresponding characterization for **rCEPU5** is included in the Supporting Information (see Figures S95–S100).

**Scheme 2:**
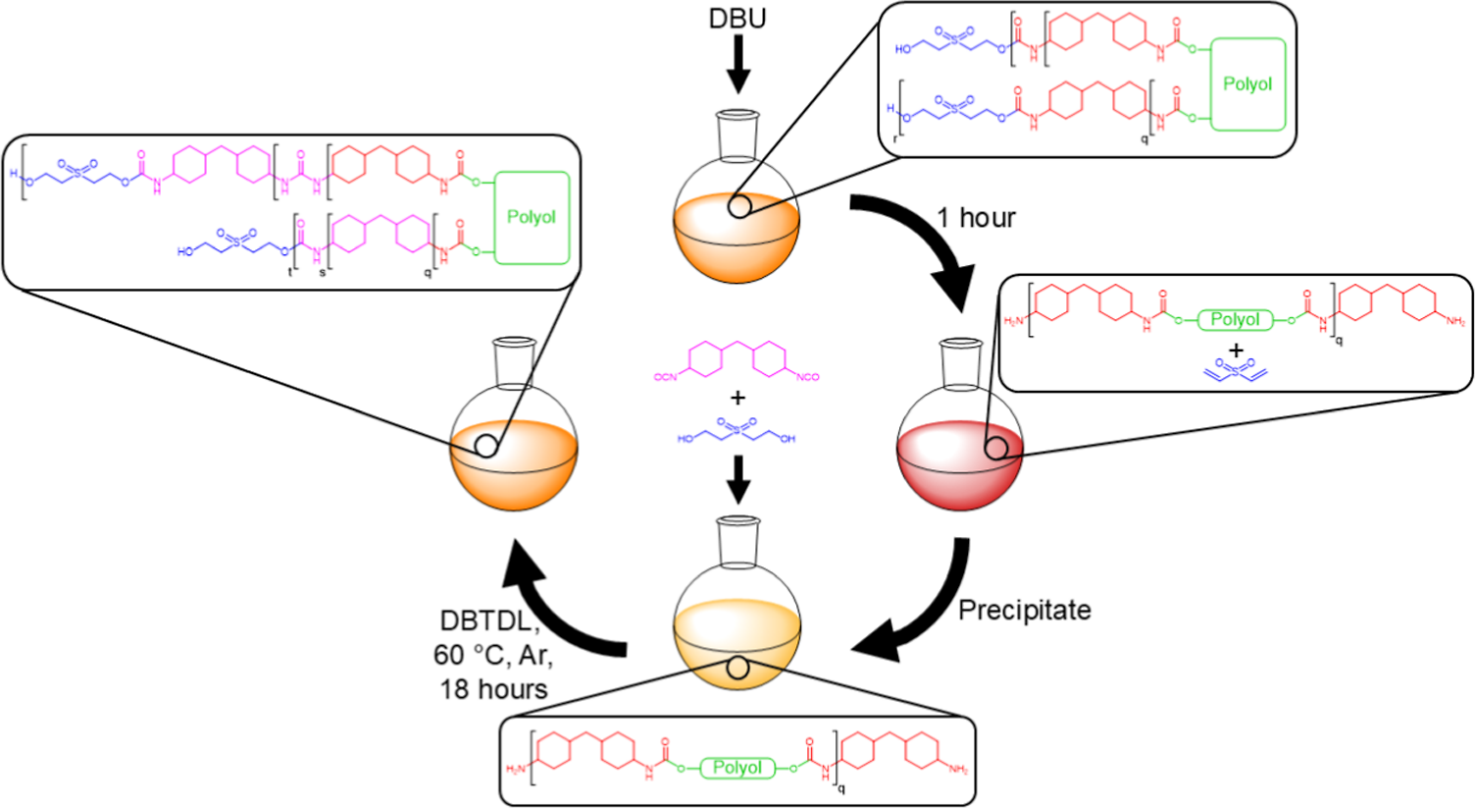
General Recycling Protocol to Afford a Recycled CEPU (rCEPU)

NMR spectroscopic analysis of **rCEPU5** confirmed the incorporation of the chain-extender into the recycled polymer, with GPC analysis also revealing chain extension with the material possessing a broad monomodal distribution (*M*_n_ = 18,400 ± 200 g mol^–1^ and *D̵* = 4.63, see Table 4 for all molecular weight information). The thermal stability and thermal transitions of **rCEPU5** are comparable with the pristine **CEPU5**, see Table 1 and Supporting Information Figures S101–S104. Solution state degradation of **rCEPU5** was studied via ^1^H NMR spectroscopy and GPC analysis, after exposure to base the sulfone urethane resonances were not evident in the ^1^H NMR spectra, and 30 min after exposure to TBAF an 81% loss in *M*_w_ was observed, from 85,200 ± 4100 g mol^–1^ to 16,600 ± 700 g mol^–1^, with minimal changes in the molecular weight observed via GPC analysis thereafter, see Table 4 and Supporting Information Figures S105–S108. The final *M*_n_, *M*_w_, and *D̵* values of the degraded **rCEPU5** infer that chain-extension of the prepolymer had not occurred during the synthesis.

**Table 4:** GPC Molecular Weight and Dispersity Data for Pristine **CEPU5** and **rCEPU5**, the Order of the Data is as Follows: Pristine **CEPU5**, Isolated **CEPU5** Prepolymer, Pristine **rCEPU5**, Degraded **rCEPU5** after 30 min, 24 h, and 48 h Exposure to TBAF (the Error Shown is the Standard Deviation between the Three Repeats of Each Sample)

polymer	*M*_n_ (g mol^–1^)	*M*_w_ (g mol^–1^)	*D̵*
**CEPU5**	23,600 ± 100	45,700 ± 300	1.94
prepolymer	7300 ± 100	15,500 ± 100	2.12
**rCEPU5**	18,400 ± 200	85,200 ± 4100	4.63
30 min	7800 ± 400	16,600 ± 700	2.13
24 h	7600 ± 300	16,200 ± 400	2.13
48 h	7800 ± 400	16,400 ± 600	2.10

The adhesive capability of **rCEPU5** was investigated and carried out at 60 °C (**rCEPU5**) for 30 min on the same substrates as the pristine CEPUs, see Figure 9 and Table S7. **rCEPU5** observed the highest shear strength when adhered to glass of 7.41 ± 0.41 MPa, which is 10% lower than the pristine **CEPU5**. Debonding of the adhered **rCEPU5** was conducted using 40 wt % NaOH_(aq)_, 1 M TBAF_(aq)_, and 1 M DBU_(aq)_ solutions, with losses in shear strength up to 64% after 30 min of exposure, see Supporting Information Figure S109 and Table S8. Overall, **rCEPU5** exhibits comparable adhesive behavior (i.e., cohesive failure and shear strength) to the pristine **CEPU5** and maintains the debond-on-demand properties of the pristine CEPU, exemplifying the recyclability of CEPUs synthesized utilizing the 2,2′-sulfonyldiethanol chain-extender.

**Figure 9:**
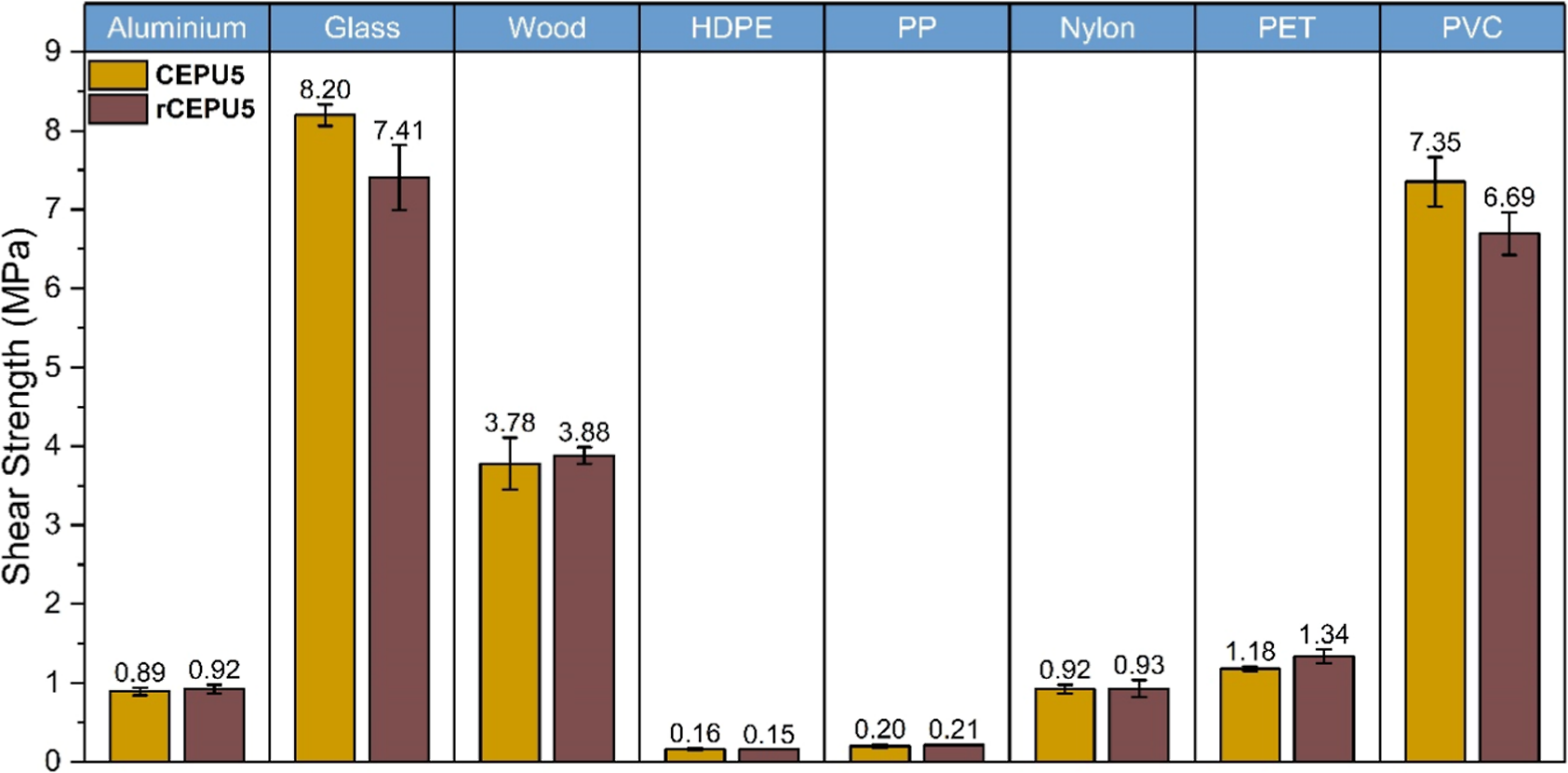
Shear strength of **CEPU5** and **rCEPU5** on aluminum, glass, wood, high density poly­(ethylene) (HDPE), poly­(propylene) (PP), Nylon, polyethylene terephthalate (PET), and polyvinyl chloride (PVC). The error shown is the standard deviation between the three repeats of each sample.

## 3 Conclusions

To address current industrial and commercial needs for rapidly degradable, recyclable, and strongly adhering polymers, a series of base-triggered, depolymerizable and recyclable CEPUs featuring the commercially available 2,2′-sulfonyldiethanol chain-extender have been generated for use as ‘debond-on-demand’ adhesives and as binders for CIJ printing. Varying the CEPU composition by changing polyol backbones facilitated the tailoring of thermal, mechanical, and adhesive properties as well as the solubility of the CEPUs. CEPU films possessed excellent mechanical properties, with ultimate tensile strengths of up to 21.52 MPa and elongation at breaks of up to 19.50 ε. **CEPU5** was shown to, in some cases, significantly outperform comparable ‘debond-on-demand’ adhesives described in the literature when adhered to glass substrates, achieving an adhered shear strength of 8.20 MPa. Upon exposure to TBAF, rapid depolymerization of the CEPUs occurs with losses in *M*_w_ of 88% after only 30 min, from 167,800 g mol^–1^ to 19,600 g mol^–1^. The base-triggered ‘debond-on-demand’ characteristics of the CEPUs were investigated, yielding losses in shear strength of 92%, from 8.20 to 0.62 MPa, after only 30 min exposure to 40 wt % NaOH_(aq)_. To facilitate a more circular polymer economy, the recyclability of the CEPUs was exemplified using **CEPU5** which yielded a polymer (**rCEPU5**) with comparable thermal, adhesive and depolymerizable properties to its pristine counterpart. Upon incorporation of the CEPUs into CIJ formulations well-resolved prints on both high and low energy substrates were realized, possessing the ability to be debonded from the surface upon submerging in base solutions for only 10 min.

## 4 Experimental Section

### 4.1 Materials

Krasol HLBH-P2000 was kindly provided by Total Cray Valley and Stepanpol PC-205P-30 was kindly provided by Alfa Chemicals for this study. 2,2′-Sulfonyldiethanol was purchased from Fluorochem and dried by azeotropic distillation in vacuo with ethanol and then dried over phosphorus pentoxide prior to use. Tetrahydrofuran (THF) and acetonitrile (MeCN) were dried prior to use using an MBRAUN SP7 system fitted with activated alumina columns. All other reagents and solvents were purchased from Sigma-Aldrich and Fisher Scientific and used as received.

### 4.2 Characterization

^1^H NMR and ^13^C­{H} NMR spectra were recorded on either a Bruker Nanobay 400 or a Bruker DPX 400 spectrometer operating at 400 MHz for ^1^H NMR or 100 MHz for ^13^C­{H} NMR, respectively. The data were processed using MestReNova Version 14.2.1-27684. Samples for NMR spectroscopic analysis were prepared in MeCN-*d*_3_ and THF-*d*_8_, and dissolution of the samples was aided with gentle heating. Chemical shifts (δ) are reported in ppm relative to the residual solvent resonance (δ 1.94 ppm) for MeCN-*d*_3_ and (δ 3.58 ppm) for THF-*d*_8_ in ^1^H NMR, *J* values are given in Hz. Infrared (IR) spectroscopic analysis was carried out using a PerkinElmer 100 FT-IR (Fourier Transform Infrared) instrument with a diamond-ATR sampling accessory. Mass spectrometry (MS) was conducted using a Thermo Scientific LTQ-Orgitrap-XL Fourier Transform Mass Spectrometer (FT-MS). The sample was introduced by an Agilent 1100 HPLC, and sample ionization was achieved by electrospray ionization (ESI). Melting points were recorded using Stuart MP10 melting point apparatus and are uncorrected. Gel permeation chromatography (GPC) analysis was conducted on an Agilent Technologies 1260 Infinity system using HPLC -grade THF at a flow rate of 1.0 mL min^–1^, calibration was achieved using a series of near monodisperse polystyrene standards, and samples were prepared at a concentration of 1 mg mL^–1^. Thermogravimetric analysis (TGA) was carried out on a TA Instruments TGA Q50 instrument with aluminum Tzero pans. The sample was heated from 20 to 550 °C at 10 °C min^–1^ under nitrogen gas at a flow rate of 100 mL min^–1^. Differential scanning calorimetry (DSC) measurements were performed on a TA Instruments X3DSC adapted with a TA Refrigerated Cooling System 90 or a TA Instruments Discovery DSC 25 TA Instruments, using aluminum Tzero pans and lids from −80 to 200 °C (**CEPU1**, **CEPU2**, **CEPU5**, and **rCEPU5**) or from −90 to 200 °C (**CEPU3** and **CEPU4**) with a heating rate of 10 °C min^–1^. Data was processed using TA Instruments Trios software version v5.8.0.41. SAXS and WAXS experiments were conducted on beamline I22 at Diamond Light Source (Harwell, UK).^68^ Samples were mounted in modified DSC pans in a Linkam 600 DSC stage for temperature control. SAXS data was collected with a Pilatus P3-2 M detector and WAXS data was collected with a Pilatus 3-2M-DLS-L detector. VT-SAXS and VT-WAXS experiments were conducted from 20 to 200 °C with a heating and cooling rate of 10 °C min^–1^ with spectra collected at 5 °C intervals. SAXS data was reduced using the software DAWN^69^ and fitting was achieved using SasView Version 5.0.6 (www.sasview.org/) using a shape independent broad peak function to obtain *q*_max_.

The scattering intensity (*I*) in a shape independent broad peak model is calculated as

$$I=\frac{A}{q^{n}}+{\left(\frac{C}{1+{\left(\left|q-q_{0}\right|\xi \right)}^{m}}\right)}^{p}+B$$

Where: *A* = Porod law scale factor, *q* = scattering vector, *n* = Porod exponent, *C* = Lorentzian scale factor, *q*_0_ = peak position, *m* = exponent of *q*, ξ = Screening length, *B* = flat background, and *p* generalizes the model to allow interpolation between a Lorentzian and Debye Anderson Brumberger (DAB) peak.

*d*-spacing was calculated using the following equation

$$d=\frac{2\pi }{q_{0}}$$

Solid state rheological measurements were performed on a Malvern Panalytical Kinexus Lab+ instrument fitted with a Peltier plate cartridge and 8 mm parallel plate geometry and analyzed using rSpace Kinexus v1.76.2398 software. Tensile tests were carried out using a Thümler Z3-X1200 tensometer at a rate of 10 mm min^–1^ with a 1 KN load cell and THSSD-2021 software. The modulus of toughness was calculated by integrating the recorded plot to give the area under the curve. The trapezium rule was applied to calculate the area between zero strain to strain at break for each sample. The error reported is the standard deviation between the three repeats for each sample. HSP analysis was conducted using 10 wt % of polymer in 27 different solvents, with solvation allowed to occur at room temperature for 24 h with shaking. Samples were subsequently denoted as either being fully dissolved (1) or insoluble (0) to input into HSPiP sixth Edition software version 6.0.04.^70^ Contact angle measurements and corresponding surface free energies were measured using a KRUSS Mobile Surface Analyzer using a double sessile drop method with 2 μL droplets of HPLC grade water and diiodomethane at 20 °C, measurements were collected 3 s post droplet deposition. All data was collected using KRUSS ADVANCE software version 1.14.1.16701, with surface free energies calculated using the OWRK model within the software. All untreated surfaces were cleaned thoroughly with isopropanol directly prior to testing. The error reported is the standard deviation between the three repeats for each sample. Viscosity measurements were made using a Brookfield Ametek DVNext rheometer at 25 °C using a speed of 60 rpm (shear rate 73.38 s^–1^). Conductivity was measured using a Mettler Toledo Seven Compact Conductivity meter at 25 °C. Density measurements were conducted on an Anton Paar DSA 5000 M Density and Sound Velocity meter at 25 °C. Piezo Axial Vibrator (PAV) rheology was conducted using a TriPAV High frequency Rheometer connected to a Stanford Research Systems SR860 Lock-in amplifier at 25 °C from 10 to 10,000 Hz, utilizing a 50 μm steel spacer shim (real sample thickness 26.27 μm) and a drive amplitude of 2 V. TriPAV software version v1.1.1-2.08 was used with temperature control achieved using a TriPAV heating/cooling jacket and a Kruss PT80 Heating/Cooling circulator. Calibration was achieved using Silicone S6 Newtonian standard with a viscosity of 7.21 cP at 25 °C. Eighteen sets of values were recorded at each frequency with the average of the last 6 used for each data point. Continuous inkjet (CIJ) printing was conducted using a Domino Ax350i with an i-pulse print head, fitted with a 60 μm nozzle and modified to run from compressed air. Jetting pressure was set at 3 bar and the modulation voltage was set to auto modulate, whereby the printer utilized the maximum value on a modulation voltage vs break up time graph.

## Supplementary Material



## Data Availability

The data underlying this study are available in the published article and its Supporting Information.

## Notes

Experimental procedures; ^1^H and ^13^C NMR spectra, GPC data, TGA data, DSC data, SAXS and WAXS profiles, rheological data, tensile testing data, formulation analysis (PDF)
