# Supplementary material for: Recyclable Base-Triggered “Debond-on-Demand” Aliphatic Polyurethane Adhesives: Engineering Adhesion for Use in Inkjet Formulations
Source: ACS Appl Eng Mater. 2025 Jul 17;3(8):2550–63. doi: 10.1021/acsaenm.5c00390 (PMC12379157; doi:10.1021/acsaenm.5c00390)
Supplement: Supplementary file 1 [file em5c00390_si_001.pdf]

Supporting Information

Recyclable base-triggered 'debond-on-demand' aliphatic polyurethane adhesives: Engineering adhesion for use in inkjet formulations

Matthew J. Hyder,[a] Jessica Godleman,[b] Andrew Kyriacou,[b] Stuart W. Reynolds,[b] James E. Hallett,[a] Thomas Zinn,[c] Josephine L. Harries,[b] Wayne Hayes[a,*]

[a]Department of Chemistry, University of Reading, Whiteknights, Reading, RG6 6DX, UK

[b]Domino UK Ltd, Trafalgar Way, Bar Hill, Cambridge, CB23 8TU, U.K.

[c]Diamond Light Source, Diamond Light Source Ltd, Harwell Science & Innovation Campus, Didcot, OX11 0DE, UK

*Corresponding author: email address w.c.hayes@reading.ac.uk

Table of Contents

Synthesis of sulfonylbis(ethane-2,1-diyl) bis(cyclohexylcarbamate) (**2**)[1] S9

Synthesis of sulfonylbis(ethane-2,1-diyl) bis(cyclohexyl(methyl)carbamate) (**3**)[1] S9

General synthetic protocol for CEPUs (**CEPU1-CEPU5**) S10

Synthesis of polymer **CEPU1**[1] S10

Synthesis of polymer **CEPU2** S11

Synthesis of polymer **CEPU3** S11

Synthesis of polymer **CEPU4** S12

Synthesis of polymer **CEPU5** S12

Recycling protocol of CEPUs to synthesise **rCEPU5** S13

Protocol for casting CEPUs (**CEPU1-CEPU5** and **rCEPU5**) S14

Protocol for NMR solution state degradation S14

Protocol for GPC solution state degradation S14

Protocol for solid state degradation S14

Figure S 1 ^{1}H NMR spectrum of **2** (400 MHz, MeCN-d_3, 298 K)........................... S15

Figure S 2 ^{13}C {H} NMR spectrum of **2** (100 MHz, MeCN-d_3, 298 K). S15

Figure S 3 ^{1}H NMR spectrum of **3** (400 MHz, MeCN-d_3, 298 K)........................... S16

Figure S 4 ^{13}C {H} NMR spectrum of **3** (100 MHz, MeCN-d_3, 298 K). S16

Figure S 5 ^{1}H NMR spectra recorded overtime following the addition of 5 molar equiv. of DBU to a 10 mg mL^{-1} solution of model urethane **3** (400 MHz, MeCN-d_3). Descending from T=0 minutes to T=24 hours. S17

Figure S 6 ^{1}H NMR spectra recorded overtime following the addition of 5 molar equiv. of DIPEA to a 10 mg mL^{-1} solution of model urethane **2** (400 MHz, MeCN-d_3). Descending from T=0 minutes to T=24 hours. S18

Figure S 7 ^{1}H NMR spectra recorded overtime following the addition of 5 molar equiv. of DIPEA to a 10 mg mL^{-1} solution of model urethane **3** (400 MHz, MeCN-d_3). Descending from T=0 minutes to T=24 hours. S19

Figure S 8 ^{1}H NMR spectra recorded overtime following the addition of 5 molar equiv. of piperidine to a 10 mg mL^{-1} solution of model urethane **2** (400 MHz, MeCN-d_3). Descending from T=0 minutes to T=24 hours. S20

Figure S 9 ^{1}H NMR spectra recorded overtime following the addition of 5 molar equiv. of piperidine to a 10 mg mL^{-1} solution of model urethane **3** (400 MHz, MeCN-d_3). Descending from T=0 minutes to T=24 hours. S21

Figure S 10 ^{1}H NMR spectra recorded overtime following the addition of 5 molar equiv. of pyridine to a 10 mg mL^{-1} solution of model urethane **2** (400 MHz, MeCN-d_3). Descending from T=0 minutes to T=24 hours. S22

Figure S 11 ^{1}H NMR spectra recorded overtime following the addition of 5 molar equiv. of pyridine to a 10 mg mL^{-1} solution of model urethane **3** (400 MHz, MeCN-d_3). Descending from T=0 minutes to T=24 hours. S23

Figure S 12 ^{1}H NMR spectrum of **CEPU2** (400 MHz, THF-d_8, 298 K). S24

Figure S 13 ^{13}C {H} NMR spectrum of **CEPU2** (100 MHz, THF-d_8, 298 K). S24

Figure S 14 ^{1}H NMR spectrum of **CEPU3** (400 MHz, THF-d_8, 298 K). S25

Figure S 15 ^{13}C {H} NMR spectrum of **CEPU3** (100 MHz, THF-d_8, 298 K). S25

Figure S 16 ^{1}H NMR spectrum of **CEPU4** (400 MHz, THF-d_8, 298 K). S26

Figure S 17 ^{13}C {H} NMR spectrum of **CEPU4** (100 MHz, THF-d_8, 298 K). S26

Figure S 18 ^{1}H NMR spectrum of **CEPU5** (400 MHz, THF-d_8, 298 K). S27

Figure S 19 ^{13}C {H} NMR spectrum of **CEPU5** (100 MHz, THF-d_8, 298 K). S27

Figure S 20 GPC eluogram of **CEPU1** in THF. S28

Figure S 21 GPC eluogram of **CEPU2** in THF. S28

Figure S 22 GPC eluogram of **CEPU3** in THF. S28

Figure S 23 GPC eluogram of **CEPU4** in THF. S29

Figure S 24 GPC eluogram of **CEPU5** in THF. S29

Figure S 25 TGA of **CEPU1** at 10 °C min^{-1}. S30

Figure S 26 TGA of **CEPU2** at 10 °C min^{-1}. S30

Figure S 27 TGA of **CEPU3** at 10 °C min^{-1}. S31

Figure S 28 TGA of **CEPU4** at 10 °C min^{-1}. S31

Figure S 29 TGA of **CEPU5** at 10 °C min^{-1}. S32

Figure S 30 DSC of **CEPU2** at 10 °C min^{-1}, showing the 2nd heating and cooling cycle. S32

Figure S 31 DSC of **CEPU2** at 10 °C min^{-1}, showing the 1st, 2nd, and 3rd heating and cooling cycles. S33

Figure S 32 DSC of **CEPU3** at 10 °C min^{-1}, showing the 2ndheating and cooling cycle. S33

Figure S 33 DSC of **CEPU4** at 10 °C min^{-1}, showing the 1st and 2nd heating and cooling cycle. S34

Figure S 34 DSC of **CEPU5** at 10 °C min^{-1}, showing the 1st and 2nd heating and cooling cycles. S34

Figure S 35 SAXS profile of **CEPU3** and corresponding fitl ine at 20 °C. S35

Figure S 36 SAXS profile of **CEPU4** and corresponding fitl ine at 20 °C. S35

Figure S 37 SAXS profile of **CEPU5** and corresponding fitl ine at 20 °C. S35

Table S 1 q_{max} and corresponding *d*-spacing for **CEPU1-CEPU5** at 20 °C. S36

Figure S 38 SAXS fitting residuals for **CPEU3** at 20 °C..S36

Figure S 39 SAXS fitting residuals for **CPEU4** at 20 °C..S36

Figure S 40 SAXS fitting residuals for **CPEU5** at 20 °C..S37

Figure S 41 VT-SAXS profiles of **CEPU2** recorded at 5 °C intervals from 20 °C to 150 °C at a heating and cooling rate of 10 °C min^{-1}. S37

Figure S 42 VT-WAXS profiles of **CEPU2** recorded at 5 °C intervals from 20 °C to 150 °C at a heating and cooling rate of 10 °C min^{-1}. S38

Figure S 43 VT-SAXS profiles of **CEPU3** recorded at 5 °C intervals from 20 °C to 200 °C at a heating and cooling rate of 10 °C min^{-1}. S38

Figure S 44 VT-WAXS profiles of **CEPU3** recorded at 5 °C intervals from 20 °C to 200 °C at a heating and cooling rate of 10 °C min^{-1}. S39

Figure S 45 Temperature sweep analysis of **CEPU1** using a normal force of 1 N and a frequency of 1 Hz. S39

Figure S 46 Temperature sweep analysis of **CEPU2** using a normal force of 1 N and a frequency of 1 Hz. S40

Figure S 47 Temperature sweep analysis of **CEPU3** using a normal force of 1 N and a frequency of 1 Hz. S40

Figure S 48 Temperature sweep analysis of **CEPU4** using a normal force of 1 N and a frequency of 1 Hz. S41

Figure S 49 Temperature sweep analysis of **CEPU5** using a normal force of 1 N and a frequency of 1 Hz. S41

Figure S 50 Representative stress-strain curves for **CEPU1**. S42

Figure S 51 Representative stress-strain curves for **CEPU2**. S42

Figure S 52 Representative stress-strain curves for **CEPU3**. S42

Figure S 53 Representative stress-strain curves for **CEPU4**. S43

Figure S 54 Representative stress-strain curves for **CEPU5**. S43

Figure S 55 ^{1}H NMR spectra showing the solution degradation of **CEPU1** with DBU, (400 MHz, THF-d_8). Top spectrum shows the pristine CEPU, bottom spectrum shows the degraded CEPU. S44

Figure S 56 ^{1}H NMR spectra showing the solution degradation of **CEPU2** with 40 wt.% NaOD in D_2O, (400 MHz, THF-d_8). Top spectrum shows the pristine CEPU, bottom spectrum shows the degraded CEPU. S44

Figure S 57 ^{1}H NMR spectra showing the solution degradation of **CEPU2** with 1 M TBAF in THF, (400 MHz, THF-d_8). Top spectrum shows the pristine CEPU, bottom spectrum shows the degraded CEPU. S45

Figure S 58 ^{1}H NMR spectra showing the solution degradation of **CEPU2** with DBU, (400 MHz, THF-d_8). Top spectrum shows the pristine CEPU, bottom spectrum shows the degraded CEPU. S45

Figure S 59 ^{1}H NMR spectra showing the solution degradation of **CEPU3** with 40 wt.% NaOD in D_2O, (400 MHz, THF-d_8). Top spectrum shows the pristine CEPU, bottom spectrum shows the degraded CEPU. S46

Figure S 60 ^{1}H NMR spectra showing the solution degradation of **CEPU3** with 1 M TBAF in THF, (400 MHz, THF-d_8). Top spectrum shows the pristine CEPU, bottom spectrum shows the degraded CEPU. S46

Figure S 61 ^{1}H NMR spectra showing the solution degradation of **CEPU3** with DBU, (400 MHz, THF-d_8). Top spectrum shows the pristine CEPU, bottom spectrum shows the degraded CEPU. S47

Figure S 62 ^{1}H NMR spectra showing the solution degradation of **CEPU4** with 40 wt.% NaOD in D_2O, (400 MHz, THF-d_8). Top spectrum shows the pristine CEPU, bottom spectrum shows the degraded CEPU. S47

Figure S 63 ^{1}H NMR spectra showing the solution degradation of **CEPU4** with 1 M TBAF in THF, (400 MHz, THF-d_8). Top spectrum shows the pristine CEPU, bottom spectrum shows the degraded CEPU. S48

Figure S 64 ^{1}H NMR spectra showing the solution degradation of **CEPU4** with DBU, (400 MHz, THF-d_8). Top spectrum shows the pristine CEPU, bottom spectrum shows the degraded CEPU. S48

Figure S 65 ^{1}H NMR spectra showing the solution degradation of **CEPU5** with 40 wt.% NaOD in D_2O, (400 MHz, THF-d_8). Top spectrum shows the pristine CEPU, bottom spectrum shows the degraded CEPU. S49

Figure S 66 ^{1}H NMR spectra showing the solution degradation of **CEPU5** with 1 M TBAF in THF, (400 MHz, THF-d_8). Top spectrum shows the pristine CEPU, bottom spectrum shows the degraded CEPU. S49

Figure S 67 ^{1}H NMR spectra showing the solution degradation of **CEPU5** with DBU, (400 MHz, THF-d_8). Top spectrum shows the pristine CEPU, bottom spectrum shows the degraded CEPU. S50

Figure S 68 GPC eluogram of **CEPU2** in THF as a pristine sample and 30 min, 24 hr, and 48 hr post addition of TBAF. S50

Figure S 69 GPC eluogram of **CEPU3** in THF as a pristine sample and 30 min, 24 hr, and 48 hr post addition of TBAF. S51

Figure S 70 GPC eluogram of **CEPU4** in THF as a pristine sample and 30 min, 24 hr, and 48 hr post addition of TBAF. S51

Figure S 71 GPC eluogram of **CEPU5** in THF as a pristine sample and 30 min, 24 hr, and 48 hr post addition of TBAF. S52

Table S 2 M_n and M_w of **CEPU1-CEPU5** as pristine samples and 30 min, 24 hr, and 48 hr post addition of TBAF acquired from a THF GPC; the recorded are averages of three separate samples of each CEPU. The error shown is the standard deviation between the three repeats of each sample. S52

Figure S 72 Glass adhered shear strength of **CEPU5** compared to other debond-on-demand adhesives. [2–9] S53

Table S 3 Shear strength of the best CEPU adhesive on aluminium, glass, high density poly(ethylene) (HDPE), poly(propylene) (PP), Nylon, polyethylene terephthalate (PET), and polyvinyl chloride (PVC) as the pristine sample and after exposure to deionised water, 40 wt.% NaOH(aq), 1 M TBAF(aq), and 1 M DBU(aq). Percentages shown are the variation between the pristine and treated samples. The error shown is the standard deviation between the three repeats of each sample. S54

Table S 4 Solubility of **CEPU1-CEPU5** in a range of organic solvents after 24 hours. S55

Figure S 73 HSP solubility sphere of **CEPU1**. S56

Figure S 74 HSP solubility sphere of **CEPU2**. S57

Figure S 75 HSP solubility sphere of **CEPU3**. S58

Figure S 76 HSP solubility sphere of **CEPU4**. S59

Figure S 77 HSP solubility sphere of **CEPU5**. S60

Figure S 78 Water contact angles on CEPU coated surfaces. The error shown is the standard deviation between the three repeats of each sample. S61

Figure S 79 Diiodomethane contact angles on CEPU coated surfaces. The error shown is the standard deviation between the three repeats of each sample. S61

Figure S 80 Surface free energy of CEPU coated surfaces. The error shown is the standard deviation between the three repeats of each sample. S62

Table S 5 Water contact angles, diiodomethane contact angles, and surface free energies of uncoated and CEPU coated surfaces. The error shown is the standard deviation between the three repeats of each sample. S63

Figure S 81 ^{1}H NMR stability of model small molecule **1** with $TBAPF_6$ over 1 year. S64

Figure S 82 ^{1}H NMR stability of model small molecule **1** with TBAN over 1 year. . S65

Figure S 83 PAV rheology of inkjet formulations of **CEPU2-CEPU5** from 10-10000 Hz at 25 °C. **A** Storage modulus (G’), **B** Loss modulus (G’’), **C** Complex viscosity, and **D** Percentage viscosity. S66

Figure S 84 CIJ jet stream break-up of inkjet formulations; **A CEPU2**, **B CEPU3**, **C CEPU4**, **D CEPU5**. S67

Figure S 85 CIJ deposition of **CEPU2** formulations onto aluminium, glass, high density poly(ethylene) (HDPE), poly(propylene) (PP), Nylon, polyethylene terephthalate (PET), and polyvinyl chloride (PVC). S67

Figure S 86 CIJ deposition of **CEPU3** formulations onto aluminium, glass, high density poly(ethylene) (HDPE), poly(propylene) (PP), Nylon, polyethylene terephthalate (PET), and polyvinyl chloride (PVC). S67

Figure S 87 CIJ deposition of **CEPU4** formulations onto aluminium, glass, high density poly(ethylene) (HDPE), poly(propylene) (PP), Nylon, polyethylene terephthalate (PET), and polyvinyl chloride (PVC). S68

Table S 6 Peel test adhesion evaluation of CEPU formulations using adhesive tape (810 grade). The amount of material removed from the surface was graded by an arbitrary value between 1 and 5 (where 5 indicated no removal of the print (excellent adhesion) and 1 indicated the complete removal of the print (very poor adhesion)). S68

Figure S 88 Treatment of **CEPU3** inkjet formulation on a glass slide using deionised water before and after 10 minutes. S68

Figure S 89 Debonding of **CEPU3** inkjet formulation form a glass slide using 40 wt.% $NaOH_{(aq)}$ before and after 10 minutes. S69

Figure S 90 Debonding of **CEPU3** inkjet formulation form a glass slide using 1 M $DBU_{(aq)}$ before and after 10 minutes. S69

Figure S 91 Treatment of **CEPU5** inkjet formulation on a glass slide using deionised water before and after 10 minutes. S70

Figure S 92 Debonding of **CEPU5** inkjet formulation form a glass slide using 40 wt.% $NaOH_{(aq)}$ before and after 10 minutes. S70

Figure S 93 Debonding of **CEPU5** inkjet formulation form a glass slide using 1 M $TBAF_{(aq)}$ before and after 10 minutes. S71

Figure S 94 Debonding of **CEPU5** inkjet formulation form a glass slide using 1 M $DBU_{(aq)}$ before and after 10 minutes. S71

Figure S 95 ^{1}H NMR spectrum of **CEPU5** prepolymer (400 MHz, THF-d_8, 298 K). S72

Figure S 96 ^{13}C {H} NMR spectrum of **CEPU5** prepolymer (100 MHz, THF-d_8, 298 K). S72

Figure S 97 GPC eluogram of **CEPU5** prepolymer in THF. S73

Figure S 98 ^{1}H NMR spectrum of **rCEPU5** (400 MHz, THF-d_8, 298 K). S73

Figure S 99 ^{13}C {H} NMR spectrum of **rCEPU5** (100 MHz, THF-d_8, 298 K). S74

Figure S 100 GPC eluogram of **rCEPU5** in THF. S74

Figure S 101 TGA of **CEPU5** prepolymer at 10 °C min^{-1}. S75

Figure S 102 TGA of **rCEPU5** at 10 °C min^{-1}. S75

Figure S 103 DSC of **CEPU5** prepolymer at 10 °C min^{-1}, showing the 1st and 2nd heating and cooling cycles. S76

Figure S 104 DSC of **rCEPU5** at 10 °C min^{-1}, showing the 1st and 2nd heating and cooling cycles. S76

Figure S 105 ^{1}H NMR spectra showing the solution degradation of **rCEPU5** with 40 wt.% NaOD in D_2O, (400 MHz, THF-d_8). Top spectrum shows the pristine CEPU, bottom spectrum shows the degraded CEPU. S77

Figure S 106 ^{1}H NMR spectra showing the solution degradation of **rCEPU5** with 1 M TBAF in THF, (400 MHz, THF-d_8). Top spectrum shows the pristine CEPU, bottom spectrum shows the degraded CEPU. S77

Figure S 107 ^{1}H NMR spectra showing the solution degradation of **rCEPU5** with DBU, (400 MHz, THF-d_8). Top spectrum shows the pristine CEPU, bottom spectrum shows the degraded CEPU. S78

Figure S 108 GPC eluogram of **rCEPU5** in THF as a pristine sample and 30 min, 24 hr, and 48 hr post addition of TBAF. S78

Table S 7 Shear strength of **CEPU5** and **rCEPU5** on Aluminium, Glass, Wood, high density poly(ethylene) (HDPE), poly(propylene) (PP), Nylon, polyethylene terephthalate (PET), and polyvinyl chloride (PVC). The error shown is the standard deviation between the three repeats of each sample. S79

Figure S 109 Shear strength of **rCEPU5** on aluminium, polyethylene terephthalate (PET), and polyvinyl chloride (PVC) as the pristine sample and after exposure to deionised water, 40 wt.% NaOH(aq), 1 M TBAF(aq), and 1 M DBU(aq). The error shown is the standard deviation between the three repeats of each sample. S79

Table S 8 Shear strength of **rCEPU5** on aluminium, polyethylene terephthalate (PET), and polyvinyl chloride (PVC) as the pristine sample and after exposure to deionised water, 40 wt.% NaOH(aq), 1 M TBAF(aq), and 1 M DBU(aq). Percentages shown are the variation between the pristine and treated samples. The error shown is the standard deviation between the three repeats of each sample. S80

References S81

Synthesis of sulfonylbis(ethane-2,1-diyl) bis(cyclohexylcarbamate) (**2**)[1]

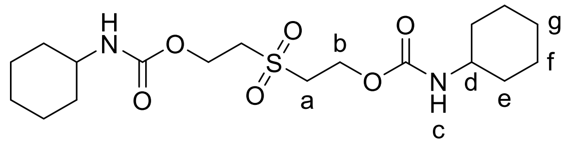

2,2'-Sulfonyldiethanol (0.53 g, 3.41 mmol, 1 equiv.) was dissolved in anhydrous THF (30 mL) to which cyclohexyl isocyanate (1.18 g, 6.82 mmol, 2 equiv.) was added dropwise at 0 °C under an argon atmosphere before being heated at 40 °C. The progress of the reaction was monitored via IR spectroscopy and once the isocyanate absorbance band at 2275-2250 cm^{-1} ($\nu N{=}C{=}O_{stretch}$) was not evident the reaction was deemed to have reached completion. The solvent was removed *in vacuo* and the crude product was recrystallised from a minimum amount of boiling ethanol. Sulfonylbis(ethane-2,1-diyl) bis(cyclohexylcarbamate) (1.18 g, 2.92 mmol, 86%) was isolated as a colourless crystalline solid. Mp 146-147 °C; FTIR ATR (cm^{-1}): 3316 ($\nu N\text{-}H_{urethane}$), 2933 ($\nu C\text{-}H_{alkyl}$), 2853 ($\nu C\text{-}H_{alkyl}$), 1687 ($\nu C{=}O_{urethane}$), 1317 ($\nu S{=}O_{stretch}$); ^{1}H NMR (400 MHz; CD_3CN) δ 5.87 (d, *J* = 8.0 Hz, 2H, H_c), 4.32 (t, *J* = 5.7 Hz, 4H, H_b), 3.35 (t, *J* = 5.7 Hz, 6H, H_a + H_d), 1.89 – 1.79 (m, 4H), 1.76 – 1.66 (m, 4H), 1.64 – 1.52 (m, 2H), 1.38 – 1.25 (m, 4H), 1.25 – 1.10 (m, 6H); ^{13}C NMR (100 MHz; CD_3CN) δ 155.9, 59.2, 54.8, 50.9, 33.8, 26.2, 25.6; FTMS (ESI) *m/z* [M + H^+] calculated for $C_{18}H_{33}N_2O_6S$ = 405.2054, found = 405.2020.

Synthesis of sulfonylbis(ethane-2,1-diyl) bis(cyclohexyl(methyl)carbamate) (**3**)[1]

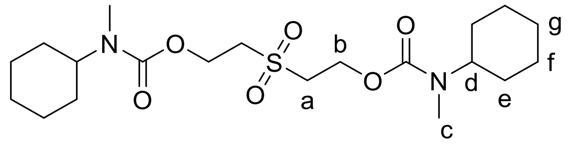

2,2'-Sulfonyldiethanol (1.50 g, 9.73 mmol, 1 equiv.) was dissolved in mixture of anhydrous THF (10 mL) and anhydrous acetonitrile (10 mL) and added dropwise to a solution of 15 wt.% phosgene in toluene (28 mL, 39.24 mmol, 4 equiv.) at -10 °C under an argon atmosphere. The solution was allowed to react at room temperature for 3 hours, the solvent was then removed *in vacuo*. The resulting chloroformate was re-dissolved in anhydrous toluene (15 mL) and added dropwise to a solution of *N*-methylcyclohexylamine (2.20 g, 19.43 mmol, 2 equiv.) in anhydrous toluene (15 mL) at 0 °C under an argon atmosphere before being refluxed at 120 °C for 16 hours. The solution was allowed to cool to room temperature, filtered then the solution was washed with water (3 x 30 mL), then 0.5 M citric acid (3 x 30 mL), then water (30 mL). The organic phase was dried with $MgSO_4$ and the solvent was removed *in vacuo*. The resulting oil was cooled in ice to induce crystallisation, the crude product was recrystallised from a minimum amount of boiling ethanol. Sulfonylbis(ethane-2,1-diyl) bis(cyclohexyl(methyl)carbamate) (1.25 g, 2.89 mmol, 30%) was isolated as a colourless crystalline solid. Mp 108-109 °C; FTIR ATR (cm^{-1}): 2937 ($\nu C\text{-}H_{alkyl}$), 2853 ($\nu C\text{-}H_{alkyl}$), 1688 ($\nu C{=}O_{urethane}$), 1475 ($\nu C\text{-}H_{alkyl}$), 1447 ($\nu C\text{-}H_{methyl}$), 1314 ($\nu S{=}O_{stretch}$);

^{1}H NMR (400 MHz; CD_3CN) δ 4.41 (t, *J* = 5.7 Hz, 4H, H_b), 3.95 – 3.74 (m, 2H, H_d), 3.39 (t, *J* = 5.7 Hz, 4H, H_a), 2.74 (s, 6H, H_c), 1.78 (d, *J* = 13.2 Hz, 4H), 1.61 (d, *J* = 13.2 Hz, 6H), 1.44 (qd, *J* = 12.1, 3.1 Hz, 4H), 1.37 – 1.21 (m, 4H), 1.09 (qt, *J* = 12.8, 3.6 Hz, 2H); ^{13}C NMR (100 MHz; CD_3CN) δ 155.9, 59.3, 56.1, 54.5, 30.6, 28.8, 26.5, 26.1; FTMS (ESI) *m/z* [M + H^+] calculated for $C_{20}H_{37}N_2O_6S$ = 433.2367, found = 433.2336.

General synthetic protocol for CEPUs (**CEPU1**-**CEPU5**)

The polyol was dried under vacuum in the oven at 80 °C for 2 hours directly prior to use. In the bulk, polyol (1.00 equiv.) was mixed with 4,4'-methylene-bis(cyclohexyl isocyanate) (4,4'-HMDI) (2.05 equiv.) and dibutyltin dilaurate (DBTDL) (10 drops) at 80 °C under an argon atmosphere for 3 hours with gentle stirring. The colourless pre-polymer obtained was dissolved in anhydrous THF (100 mL) and 2,2'-sulfonyldiethanol (1.05 equiv.) was then added to the solution which was then brought to and maintained under reflux for 18 hours under an argon atmosphere. The chain-extended polymer solution was precipitated into ice cold methanol (**CEPU1** and **CEPU5**) or diethyl ether (**CEPU2**-**CEPU4**) (3 x 1000 mL), the solid material filtered off and then dried *in vacuo*.

Synthesis of polymer **CEPU1**[1]

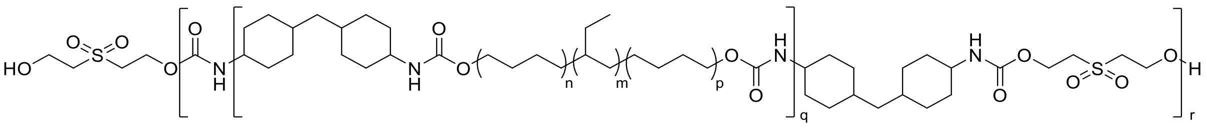

The synthesis was carried out according to the general synthetic protocol described above for CEPUs. Hydrogenated poly(butadiene) (Krasol HLBH-P 2000), molecular weight as supplied = 2100 g mol^{-1}, was dried under vacuum in the oven at 80 °C for 2 hours directly prior to use. The polymer was isolated as a transparent colourless elastomeric solid (18.57 g, 89%) from Krasol HLBH-P 2000 (15.65 g, 7.45 mmol, 1.00 equiv.), 4,4'-methylene-bis(cyclohexyl isocyanate) (4.01 g, 15.28 mmol, 2.05 equiv.), and 2,2-sulfonyldiethanol (1.21 g, 7.85 mmol, 1.05 equiv.). T_g = -47.1 °C, T_m = 50.1 °C; FTIR ATR (cm^{-1}): 3343 (vN-$H_{stretch}$), 2960 (vC-H_{alkyl}), 2920 (vC-H_{alkyl}), 2852 (vC-H_{alkyl}), 1727 (vC=$O_{urethane}$), 1708 (vC=$O_{urethane}$), 1461 (vC-H_{alkyl}), 1379 (vC-H_{alkyl}), 1322 (vS=$O_{stretch}$); ^{1}H NMR (400 MHz; THF-d_8) δ 6.64 – 6.50 (m, 1H), 6.07 – 5.95 (m, 1H), 4.33 (m, 2H), 4.09 – 3.88 (m, 2H), 3.76 – 3.62 (m, 1H), 3.41 – 3.26 (m, 2H), 2.13 – 1.84 (m, 5H), 1.68 – 0.63 (m, 182H); ^{13}C NMR (100 MHz; THF-d_8) δ 156.6, 156.1, 64.6, 63.0, 59.4, 54.9, 51.5, 51.3, 48.5, 40.1, 39.6, 39.5, 39.0, 37.4, 35.1, 34.6, 34.4, 34.2, 33.5, 33.4, 31.8, 31.3, 31.2, 30.9, 30.6, 30.5, 29.1, 27.9, 27.7, 27.5, 27.2, 27.1, 26.9, 23.7, 11.4, 11.3, 11.2, 11.1, 11.0, 10.8; GPC (THF) M_n = 44700 ± 200 g mol^{-1}, M_w = 140400 ± 700 g mol^{-1}, Đ = 3.14.

Synthesis of polymer **CEPU2**

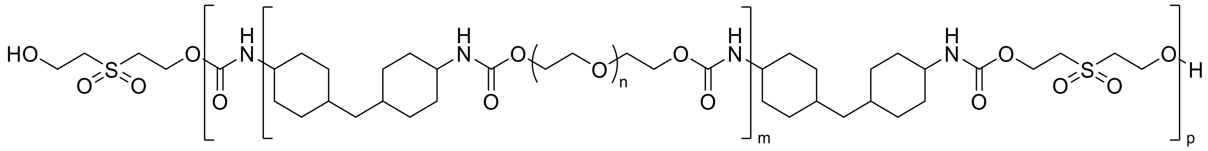

The synthesis was carried out according to the general synthetic protocol described above for CEPUs. Poly(ethylene glycol) (PEG), molecular weight as supplied = 2000 g mol^{-1}, was dried under vacuum in the oven at 80 °C for 2 hours directly prior to use. The polymer was isolated as a transparent colourless elastomeric solid (42.25 g, 70%) from PEG 2000 (45.01 g, 22.51 mmol, 1.00 equiv.), 4,4'-methylene-bis(cyclohexyl isocyanate) (12.11 g, 46.16 mmol, 2.05 equiv.), and 2,2-sulfonyldiethanol (3.64 g, 23.61 mmol, 1.05 equiv.). T_g = -51.8 °C, T_m = 25.1 °C, T_{cc} = -34.3 °C, T_c = -4.9 °C; FTIR ATR (cm^{-1}): 3326 (νN-$H_{stretch}$), 2874 (νC-H_{alkyl}), 1700 (νC=$O_{urethane}$), 1466 (νC-H_{alkyl}), 1344 (νC-H_{alkyl}), 1321 (νS=$O_{stretch}$), 1097 (νC-$O_{stretch}$); ^{1}H NMR (400 MHz; THF-d_8) δ 6.58 (d, *J* = 7.3 Hz, 1H), 6.43 – 6.34 (m, 1H), 6.30 – 6.15 (m, 15H), 5.41 – 5.21 (m, 4H), 5.16 – 4.68 (m, 7H), 4.44 – 4.14 (m, 5H), 4.06 (q, *J* = 5.6 Hz, 35H), 3.91 (t, *J* = 5.8 Hz, 26H), 3.85 – 3.75 (m, 2H), 3.69 – 3.61 (m, 5H), 3.55 (s, 1419H), 3.36 – 3.24 (m, 3H), 3.20 (t, *J* = 5.8 Hz, 23H), 1.92 (d, *J* = 11.6 Hz, 30H), 1.63 (s, 16H), 1.57 – 1.40 (m, 85H), 1.28 (t, *J* = 7.2 Hz, 37H), 1.20 – 1.04 (m, 39H), 1.04 – 0.86 (m, 45H); ^{13}C NMR (100 MHz; THF-d_8) δ 156.6, 156.5, 71.7, 71.5, 71.5, 70.8, 64.2, 58.0, 57.1, 51.3, 35.1, 34.2, 33.5, 33.4, 31.4, 30.6, 29.4, 29.1; GPC (THF) M_n = 22200 ± 100 g mol^{-1}, M_w = 62600 ± 1300 g mol^{-1}, Đ = 2.82.

Synthesis of polymer **CEPU3**

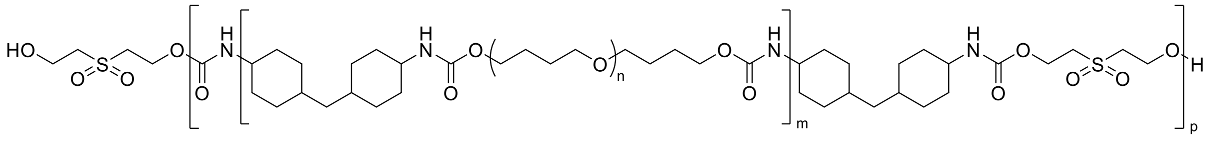

The synthesis was carried out according to the general synthetic protocol described above for CEPUs. Poly(tetrahydrofuran) (PTHF), molecular weight as supplied = 2000 g mol^{-1}, was dried under vacuum in the oven at 80 °C for 2 hours directly prior to use. The polymer was isolated as a transparent colourless elastomeric solid (45.86 g, 74%) from PTHF 2000 (45.90 g, 22.95 mmol, 1.00 equiv.), 4,4'-methylene-bis(cyclohexyl isocyanate) (12.34 g, 47.04 mmol, 2.05 equiv.), and 2,2-sulfonyldiethanol (3.72 g, 24.13 mmol, 1.05 equiv.). T_g = -74.0 °C; FTIR ATR (cm^{-1}): 3330 (νN-$H_{stretch}$), 2938 (νC-H_{alkyl}), 2854 (νC-H_{alkyl}), 2800 (νC-H_{alkyl}), 1705 (νC=$O_{urethane}$), 1448 (νC-H_{alkyl}), 1370 (νC-H_{alkyl}), 1320 (νS=$O_{stretch}$), 1100 (νC-$O_{stretch}$); ^{1}H NMR (400 MHz; THF-d_8) δ 6.50 – 6.40 (m, 1H), 6.00 – 5.90 (m, 1H), 4.33 – 4.17 (m, 3H), 3.93 – 3.77 (m, 3H), 3.63 – 3.51 (m, 1H), 3.38 – 3.12 (m, 75H), 1.81 (d, *J* = 12.3 Hz, 3H), 1.58 – 1.31 (m, 73H), 1.27 – 0.94 (m, 8H), 0.87 (s, 3H); ^{13}C NMR (100 MHz; THF-d_8) δ 156.7, 156.6, 71.5, 71.2, 64.5, 59.3, 54.9, 35.1, 34.3, 33.4, 30.6, 29.1, 29.1, 27.8, 27.5, 27.3; GPC (THF) M_n = 68400 ± 900 g mol^{-1}, M_w = 167800 ± 300 g mol^{-1}, Đ = 2.45.

Synthesis of polymer **CEPU4**

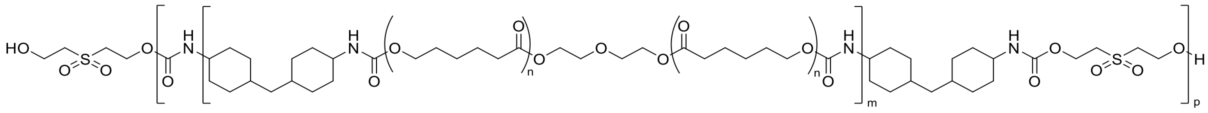

The synthesis was carried out according to the general synthetic protocol described above for CEPUs. Poly(caprolactone) (PCL), molecular weight as supplied = 2000 g mol^{-1}, was dried under vacuum in the oven at 80 °C for 2 hours directly prior to use. The polymer was isolated as an opaque colourless solid (50.03 g, 82%) from PCL 2000 (45.15 g, 22.58 mmol, 1.00 equiv.), 4,4'-methylene-bis(cyclohexyl isocyanate) (12.14 g, 46.27 mmol, 2.05 equiv.), and 2,2-sulfonyldiethanol (3.65 g, 23.67 mmol, 1.05 equiv.). T_g = -47.4 °C, T_m = 25.4 °C, T_m = 39.6 °C; FTIR ATR (cm^{-1}): 3372 (vN-$H_{stretch}$), 2932 (vC-H_{alkyl}), 2864 (vC-H_{alkyl}), 1722 (vC=O), 1463 (vC-H_{alkyl}), 1396 (vC-H_{alkyl}), 1365 (vC-H_{alkyl}), 1321 (vS=$O_{stretch}$), 1239 (vC-$O_{stretch}$), 1161 (vC-$O_{stretch}$); ^{1}H NMR (400 MHz; THF-d_8) δ 6.57 (d, J = 7.4 Hz, 4H), 6.37 (d, J = 7.3 Hz, 1H), 6.05 (t, J = 7.1 Hz, 8H), 5.29 – 4.77 (m, 3H), 4.72 (s, 1H), 4.48 – 4.29 (m, 11H), 4.02 (t, J = 6.7 Hz, 160H), 3.93 (p, J = 6.0 Hz, 19H), 3.86 (s, 17H), 3.41 – 3.13 (m, 27H), 2.35 – 2.22 (m, 170H), 1.96 – 1.87 (m, 22H), 1.61 (p, J = 6.7 Hz, 339H), 1.44 – 1.25 (m, 181H), 1.20 – 1.05 (m, 31H), 0.95 (s, 60H); ^{13}C NMR (100 MHz; THF-d_8) δ 173.4, 156.6, 156.5, 83.4, 80.8, 69.6, 64.6, 35.7, 35.1, 34.7, 34.7, 34.6, 34.3, 33.5, 30.1, 29.5, 29.1, 26.7, 26.6, 22.2; GPC (THF) M_n = 28100 ± 200 g mol^{-1}, M_w = 124300 ± 1600 g mol^{-1}, Đ = 4.42.

Synthesis of polymer **CEPU5**

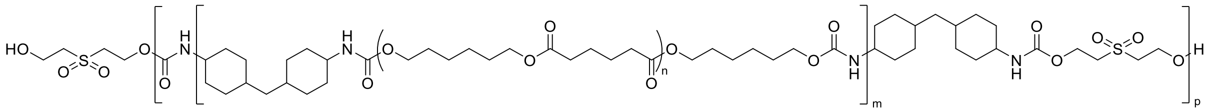

The synthesis was carried out according to the general synthetic protocol described above for CEPUs. Stepanpol PC-205P-30, molecular weight as supplied = 3740 g mol^{-1}, was dried under vacuum in the oven at 80 °C for 2 hours directly prior to use. The polymer was isolated as an opaque colourless solid (31.40 g, 87 %) from Stepanpol PC-205P-30 (30.19 g, 8.07 mmol, 1.00 equiv.), 4,4'-methylene-bis(cyclohexyl isocyanate) (4.34 g, 16.54 mmol, 2.05 equiv.), and 2,2-sulfonyldiethanol (1.31 g, 8.50 mmol, 1.05 equiv.). T_m = 43.5 °C, T_m = 48.1 °C, T_c = 32.7 °C, T_c = 32.8 °C; FTIR ATR (cm^{-1}): 3374 (vN-$H_{stretch}$), 2933 (vC-H_{alkyl}), 2865 (vC-H_{alkyl}), 1725 (vC=O), 1465 (vC-H_{alkyl}), 1370 (vC-H_{alkyl}), 1320 (vS=$O_{stretch}$), 1257 (vC-$O_{stretch}$), 1163 (vC-$O_{stretch}$); ^{1}H NMR (400 MHz; THF-d_8) δ 6.72 – 6.47 (m, 4H), 6.36 (d, J = 6.5 Hz, 1H), 6.03 (t, J = 6.8 Hz, 11H), 4.87 – 4.66 (m, 5H), 4.46 – 4.27 (m, 11H), 4.25 – 4.11 (m, 4H), 4.02 (t, J = 6.5 Hz, 392H), 3.98 – 3.88 (m, 20H), 3.72 – 3.61 (m, 9H), 3.45 – 3.19 (m, 22H), 3.16 (t, J = 5.7 Hz, 3H), 2.34 – 2.21 (m, 408H), 1.96 – 1.85 (m, 28H), 1.68 – 1.53 (m, 824H), 1.44 – 1.32 (m, 427H), 1.20 – 0.87 (m, 67H); ^{13}C NMR (100 MHz; THF-d_8) δ 173.3, 157.9, 156.6, 64.7, 51.3, 45.6, 34.4, 33.5, 30.6, 30.3, 29.8,

29.7, 29.1, 26.8, 26.7; GPC (THF) M_n = 23600 ± 100 g mol^{-1}, M_w = 45700 ± 300 g mol^{-1}, Đ = 1.94.

Recycling protocol of CEPUs to synthesise **rCEPU5**

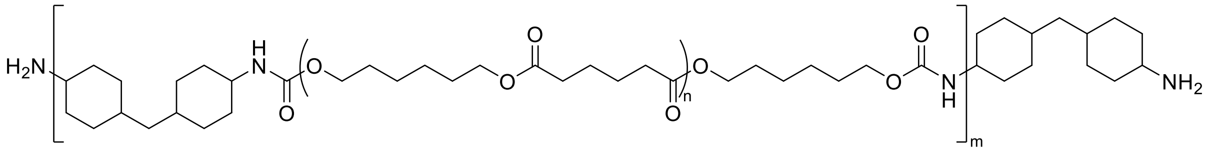

Pristine **CEPU5** (9.99 g, 0.42 mmol) was dissolved in anhydrous THF (30 mL) and DBU (5.6 mL, 36.06 mmol) was added at room temperature under an argon atmosphere, the solution was stirred for 1 hour. The resulting polymer solution was then precipitated into ice cold methanol (5 x 750 mL), the solid material filtered off and dried *in vacuo.* The amino terminated prepolymer was isolated as a colourless solid (8.09 g, 81%). T_m = 40.9 °C, T_m = 50.8 °C, T_m = 50.0 °C, T_c = 38.0 °C, T_c = 37.9 °C; FTIR ATR (cm^{-1}): 3448 (vNH$_{2\ stretch}$), 3378 (vN-H$_{stretch}$), 2935 (vC-H$_{alkyl}$), 2866 (vC-H$_{alkyl}$), 1725 (vC=O), 1465 (vC-H$_{alkyl}$), 1371 (vC-H$_{alkyl}$), 1258 (vC-O$_{stretch}$), 1163 (vC-O$_{stretch}$); ^{1}H NMR (400 MHz; THF-d_8) δ 6.41 – 6.33 (m, 1H), 6.14 – 5.96 (m, 8H), 4.86 – 4.69 (m, 2H), 4.44 – 4.29 (m, 4H), 4.20 (t, *J* = 6.5 Hz, 2H), 4.02 (t, *J* = 6.5 Hz, 287H), 3.95 (q, *J* = 6.5 Hz, 10 H), 3.68 – 3.62 (m, 6H), 3.52 (s, 2H), 3.42 – 3.23 (m, 10H), 2.34 – 2.21 (m, 292H), 1.98 – 1.85 (m, 18H), 1.68 – 1.46 (m, 605H), 1.45 – 1.32 (m, 301H), 1.19 – 0.85 (m, 40H); ^{13}C NMR (100 MHz; THF-d_8) δ 173.3, 157.8, 64.7, 35.1, 34.4, 33.5, 30.3, 29.7, 29.1, 26.8; GPC (THF) M_n = 7300 ± 100 g mol^{-1}, M_w = 15500 ± 100 g mol^{-1}, Đ = 2.12.

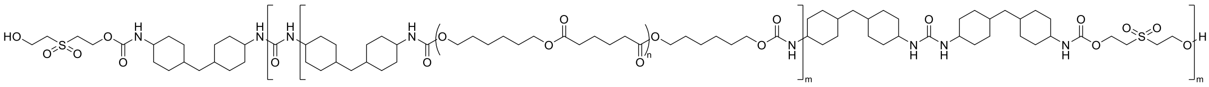

The amino terminated prepolymer (5.00 g, 0.40 mmol, 1.00 equiv.) was then redissolved in anhydrous THF (30 mL) and 4,4'-methylene-bis(cyclohexyl isocyanate) (0.65 g, 2.48 mmol, 2.05 equiv. w.r.t repeat unit), 2,2'-sulfonyldiethanol (0.20 g, 1.30 mmol, 1.05 equiv. w.r.t repeat unit), and DBTDL (3 drops) were then added under an argon atmosphere and the reaction was stirred at 60 °C for 18 hours. The recycled polymer was precipitated into ice cold methanol (3 x 750 mL) and the solid material was filtered off and dried *in vacuo*. The polymer was isolated as a colourless solid (5.33 g, 91%). T_m = 49.1 °C, T_m = 46.5 °C, T_c = 29.7 °C, T_c = 30.1 °C; FTIR ATR (cm^{-1}): 3368 (vN-H$_{stretch}$), 2932 (vC-H$_{alkyl}$), 2863 (vC-H$_{alkyl}$), 1726 (vC=O), 1398 (vC-H$_{alkyl}$), 1370 (vC-H$_{alkyl}$), 1320 (vS=O$_{stretch}$), 1256 (vC-O$_{stretch}$), 1164 (vC-O$_{stretch}$); ^{1}H NMR (400 MHz; THF-d_8) δ 6.58 (s, 3H), 6.15 – 5.98 (m, 2H), 4.86 – 4.66 (m, 1H), 4.44 – 4.27 (m, 6H), 4.02 (t, *J* = 6.5 Hz, 69H), 3.95 (q, *J* = 6.5 Hz, 3H), 3.73 – 3.60 (m, 1H), 3.33 (s, 10H), 2.36 – 2.16 (m, 70H), 2.01 – 1.82 (m, 7H), 1.66 – 1.46 (m, 150H), 1.44 – 1.32 (m, 72H), 1.22 – 0.86 (m, 20H); ^{13}C NMR (100 MHz; THF-d_8) δ 173.3, 157.8, 156.6, 64.7, 64.5, 59.3, 54.9, 51.3, 35.1, 35.1, 34.4, 34.3, 33.5, 33.4, 31.4, 30.6, 30.3, 29.8, 29.7, 29.1, 26.7; GPC (THF) M_n = 18400 ± 200 g mol^{-1}, M_w = 85200 ± 4100 g mol^{-1}, Đ = 4.63.

Protocol for casting CEPUs (**CEPU1**-**CEPU5** and **rCEPU5**)

The precipitated polymer was dissolved in a minimum volume of THF (approximately 3 mL per 1 g of polymer) at 40 °C whilst stirring. Once fully dissolved, the polymer solution was poured into a 15 cm x 15 cm mould with a PTFE base. The solvent was allowed to slowly evaporate over 24 hours at room temperature and pressure. The mould was placed into a vacuum oven at 60 °C for 24 hours, then under partial vacuum (approximately 800 mbar) at 60 °C for 24 hours, the polymer film was then allowed to reach room temperature before being removed from the mould.

Protocol for NMR solution state degradation

Model small molecules (**2** and **3**) were made to 10 mg mL^{-1} in MeCN-d_3 and CEPUs (**CEPU1**-**CEPU5** and **rCEPU5**) were made to 50 mg mL^{-1} in THF-d_8. The addition of 5 equiv. of either 40 wt.% NaOD in D_2O, 1 M *tert*-butylammonium fluoride (TBAF) in THF, 1,8-diazabicyclo(5.4.0)undec-7-ene (DBU), N,N-diisopropylethylamine (DIPEA), pyridine, or piperidine was added directly to the NMR tube. The ^1H NMR spectra were recorded at regular time intervals.

Protocol for GPC solution state degradation

Polymer solutions of CEPUs (**CEPU1**-**CEPU5** and **rCEPU5**) were made to 1 mg mL^{-1} in HLPC grade THF, 1 M TBAF in THF (5 equiv.) was added to the solutions and shaken prior to testing. All reported molecular weights are the averages of three separate samples of each CEPU, the error shown is the standard deviation between the three repeats of each sample.

Protocol for solid state degradation

Adhered lap shear samples were submerged in solutions of 40 wt.% $NaOH_{(aq)}$, 1 M $TBAF_{(aq)}$, or 1 M $DBU_{(aq)}$ for 30 minutes at room temperature. The polymer samples were then washed with deionised water then dried at room temperature for 12 hours under vacuum, the polymer samples were then allowed to rest at room temperature for 48 hours before being analysed.

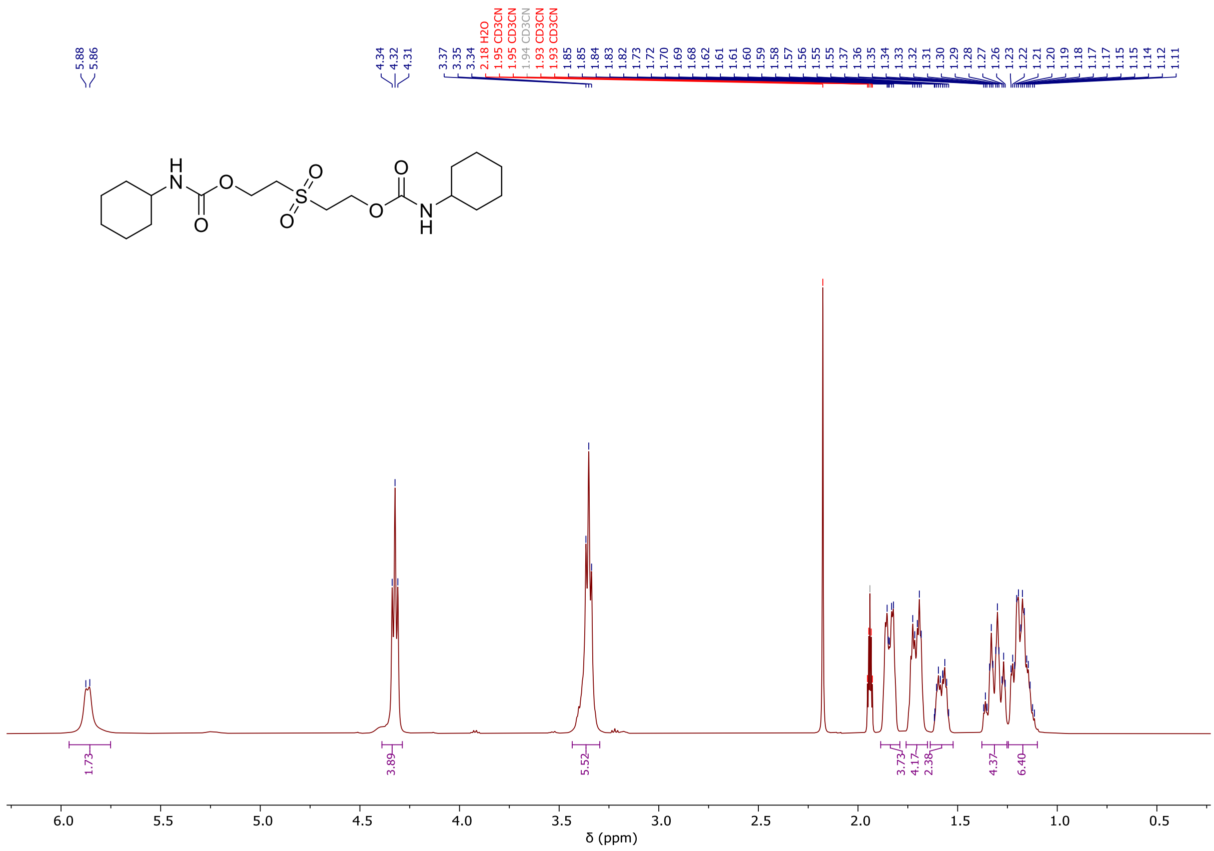

Figure S 1 ^{1}H NMR spectrum of **2** (400 MHz, MeCN-d_3, 298 K).

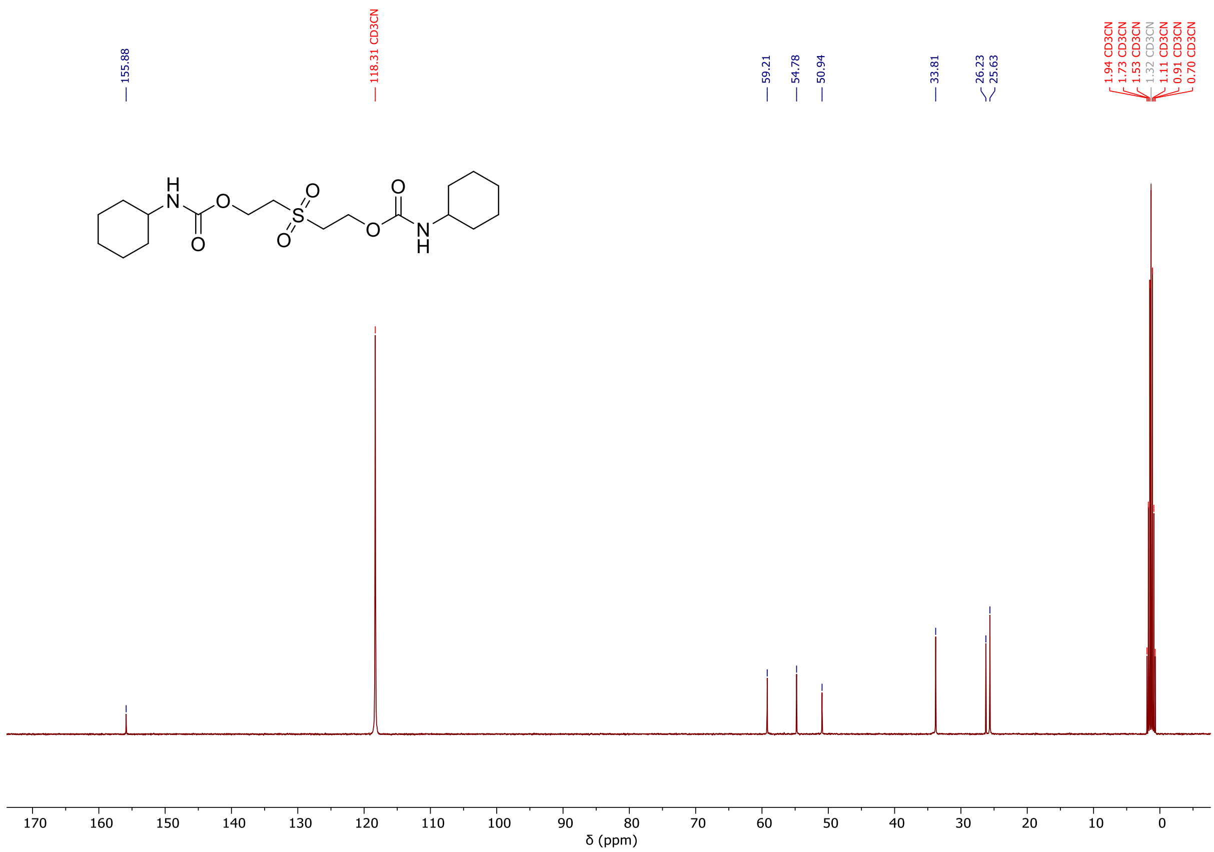

Figure S 2 ^{13}C {H} NMR spectrum of **2** (100 MHz, MeCN-d_3, 298 K).

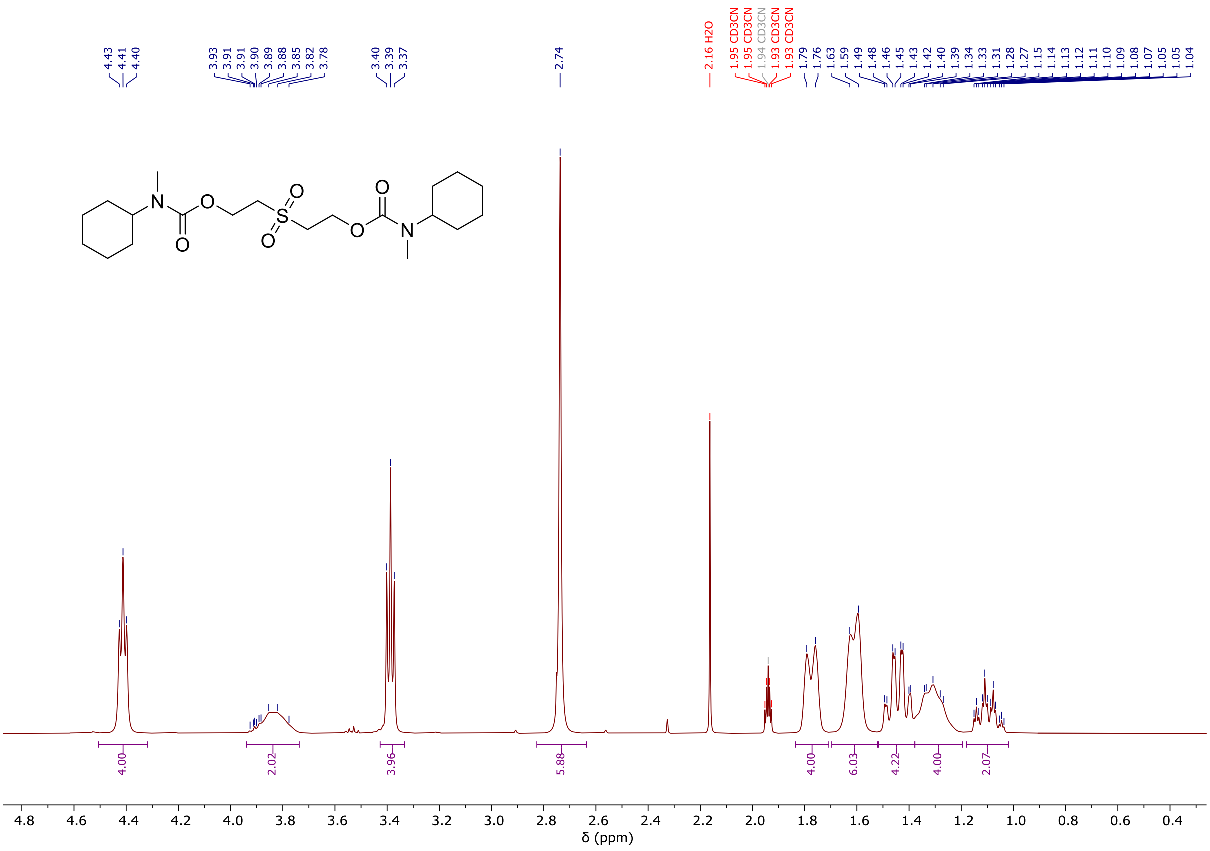

Figure S 3 ^{1}H NMR spectrum of **3** (400 MHz, MeCN-d_3, 298 K).

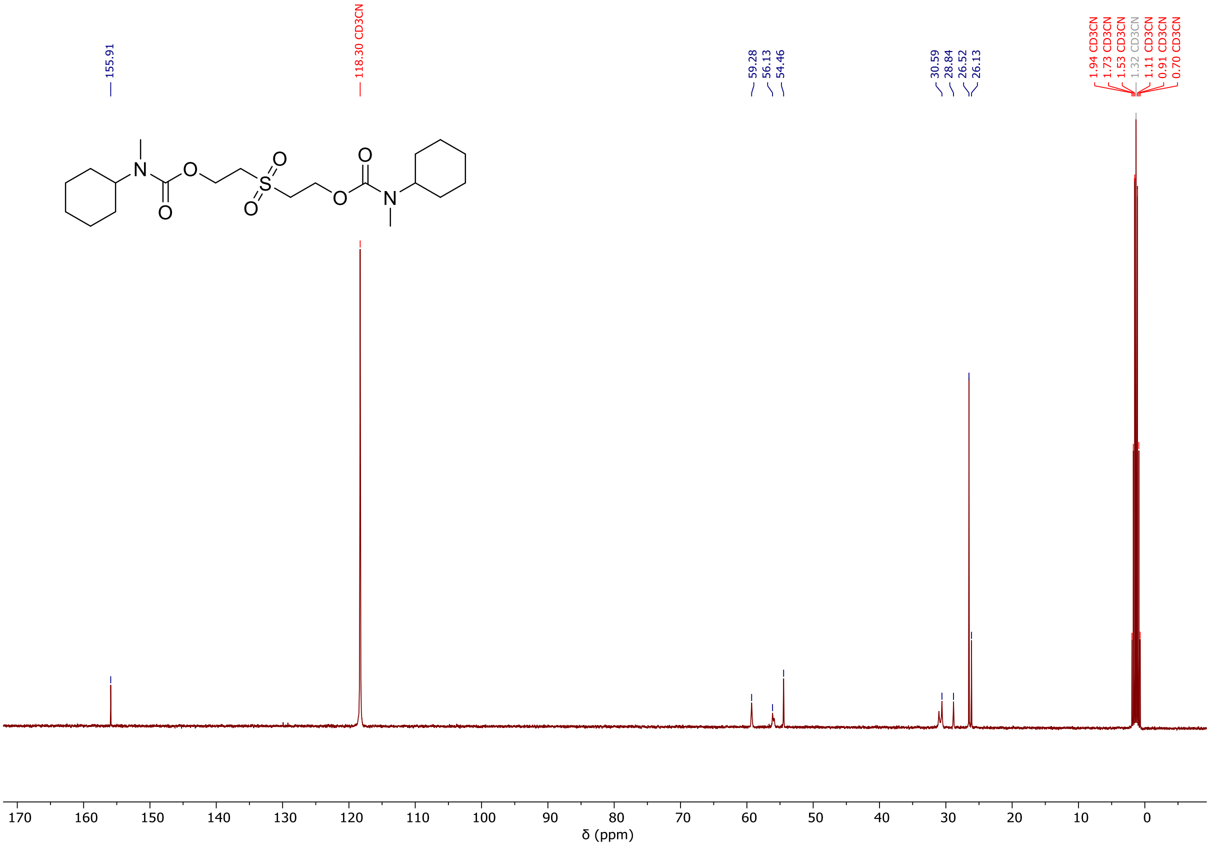

Figure S 4 ^{13}C {H} NMR spectrum of **3** (100 MHz, MeCN-d_3, 298 K).

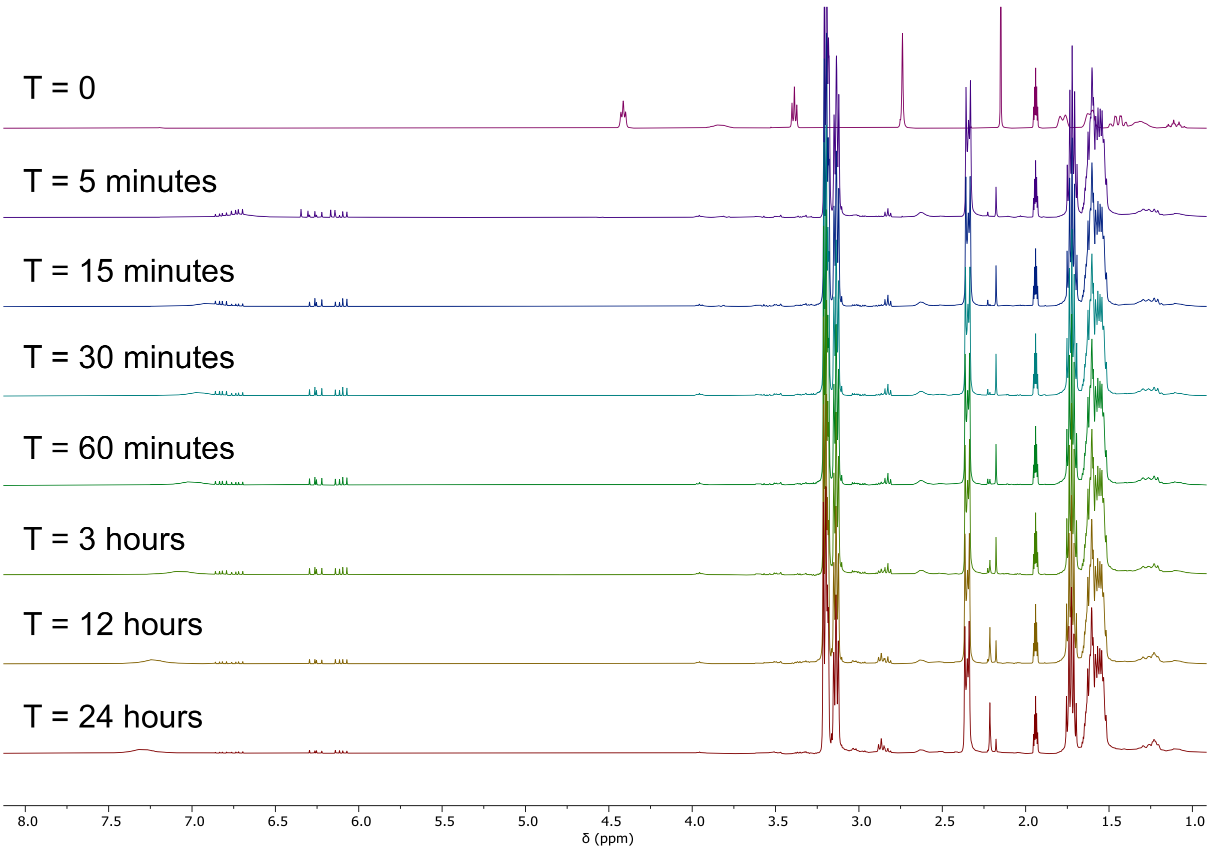

Figure S 5 ^{1}H NMR spectra recorded overtime following the addition of 5 molar equiv. of DBU to a 10 mg mL^{-1} solution of model urethane **3** (400 MHz, MeCN-d_3). Descending from T=0 minutes to T=24 hours.

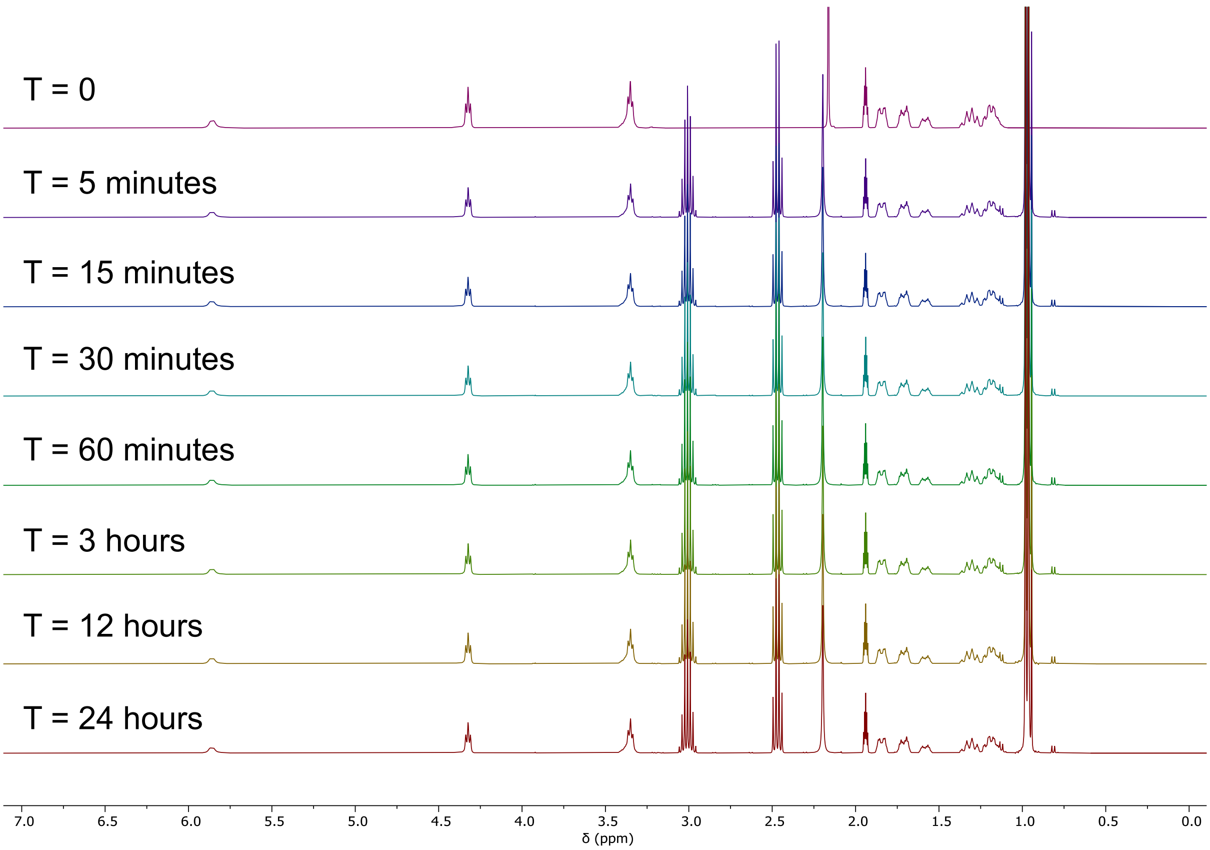

Figure S 6 ^{1}H NMR spectra recorded overtime following the addition of 5 molar equiv. of DIPEA to a 10 mg mL^{-1} solution of model urethane **2** (400 MHz, MeCN-*d*$_3$). Descending from T=0 minutes to T=24 hours.

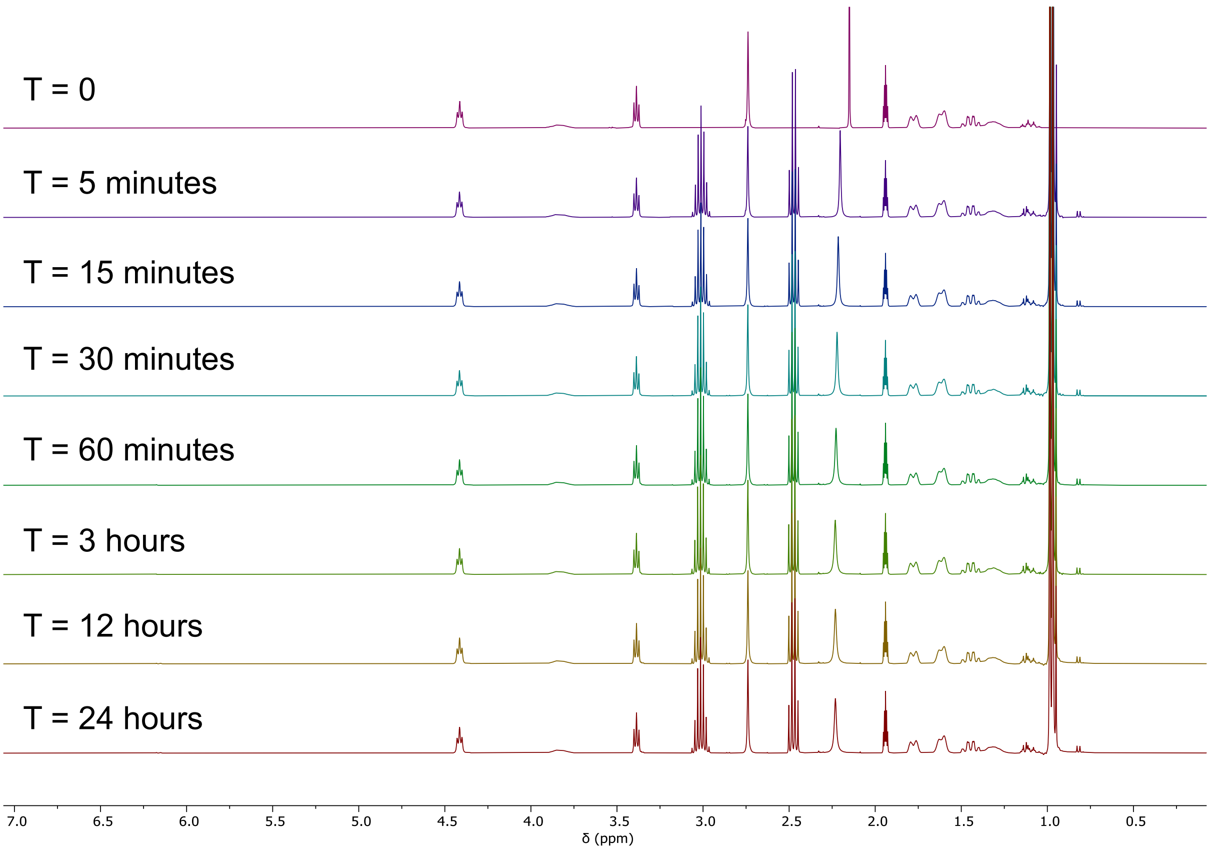

Figure S 7 ^{1}H NMR spectra recorded overtime following the addition of 5 molar equiv. of DIPEA to a 10 mg mL^{-1} solution of model urethane **3** (400 MHz, MeCN-d_3). Descending from T=0 minutes to T=24 hours.

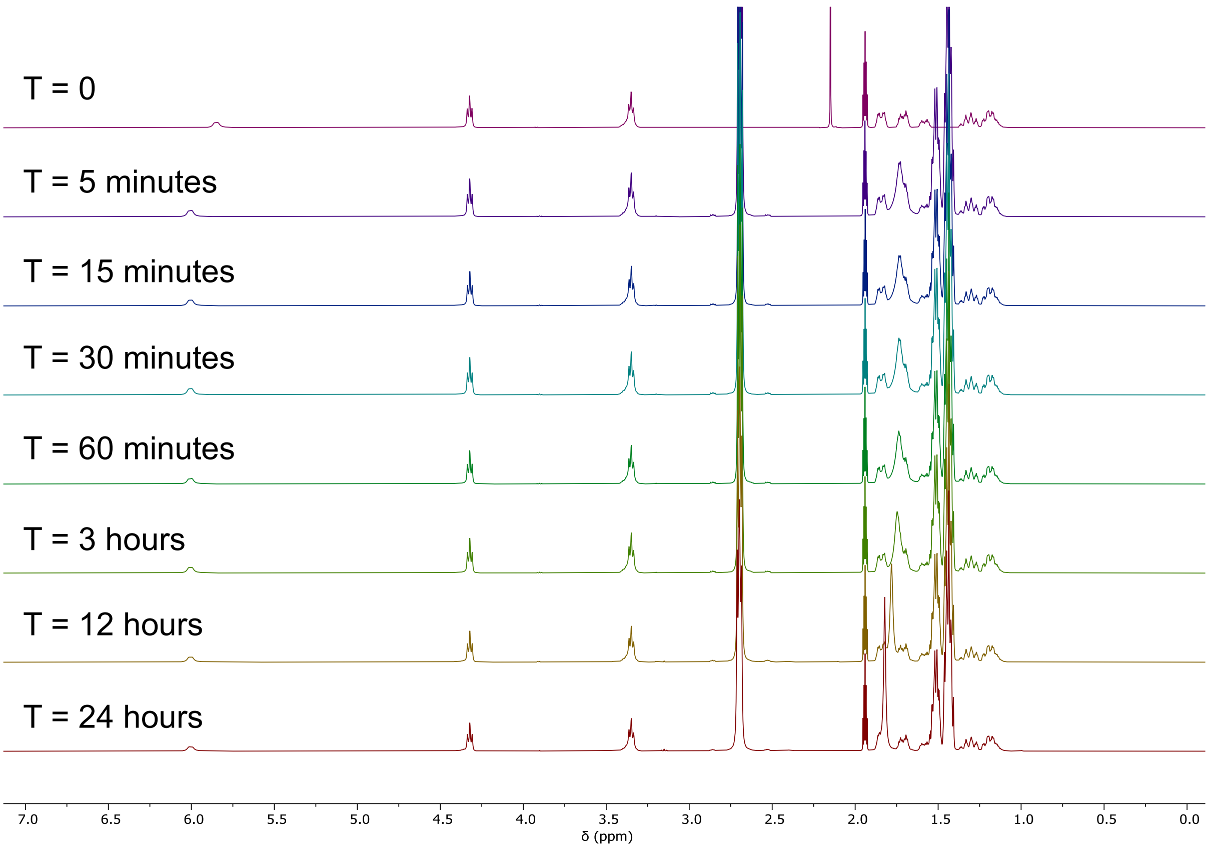

Figure S 8 ^{1}H NMR spectra recorded overtime following the addition of 5 molar equiv. of piperidine to a 10 mg mL^{-1} solution of model urethane **2** (400 MHz, MeCN-d_3). Descending from T=0 minutes to T=24 hours.

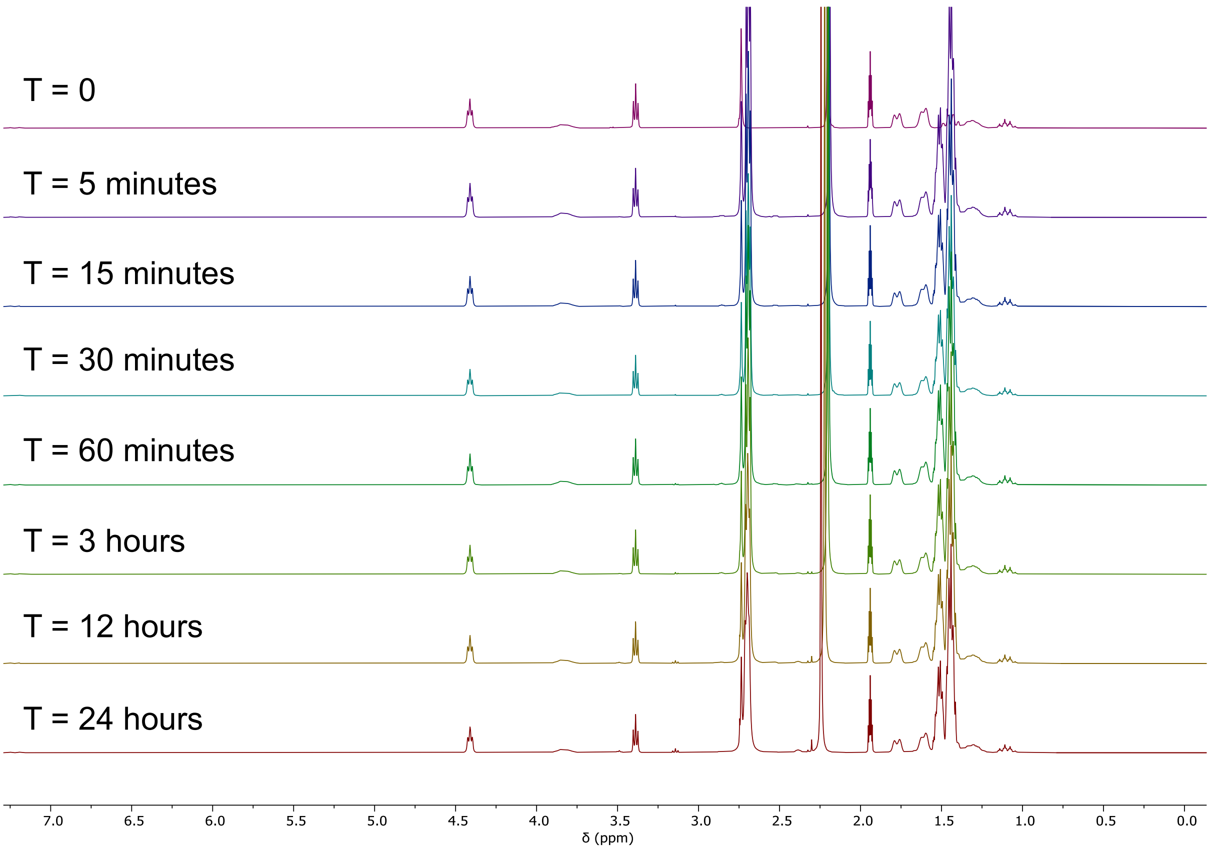

Figure S 9 ^{1}H NMR spectra recorded overtime following the addition of 5 molar equiv. of piperidine to a 10 mg mL^{-1} solution of model urethane **3** (400 MHz, MeCN-d_3). Descending from T=0 minutes to T=24 hours.

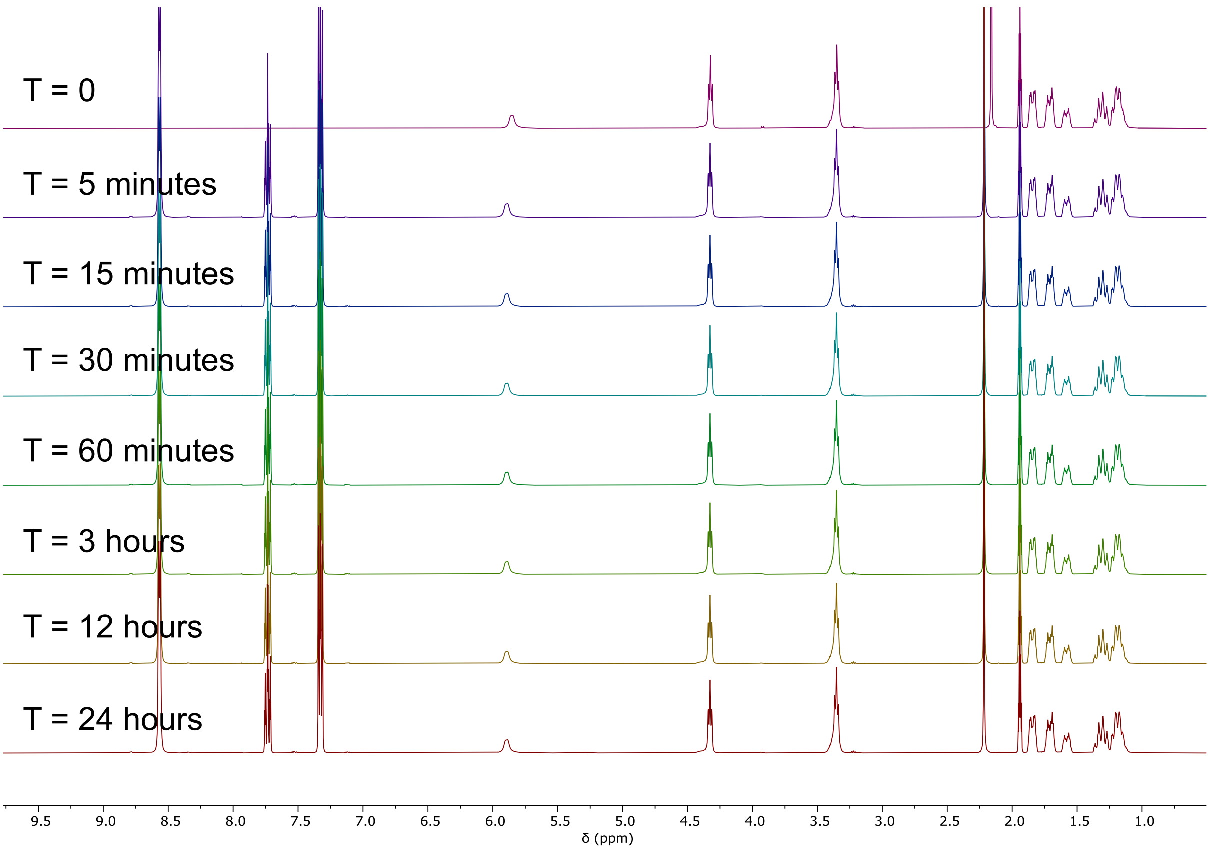

Figure S 10 ^1H NMR spectra recorded overtime following the addition of 5 molar equiv. of pyridine to a 10 mg mL^{-1} solution of model urethane **2** (400 MHz, MeCN-d_3). Descending from T=0 minutes to T=24 hours.

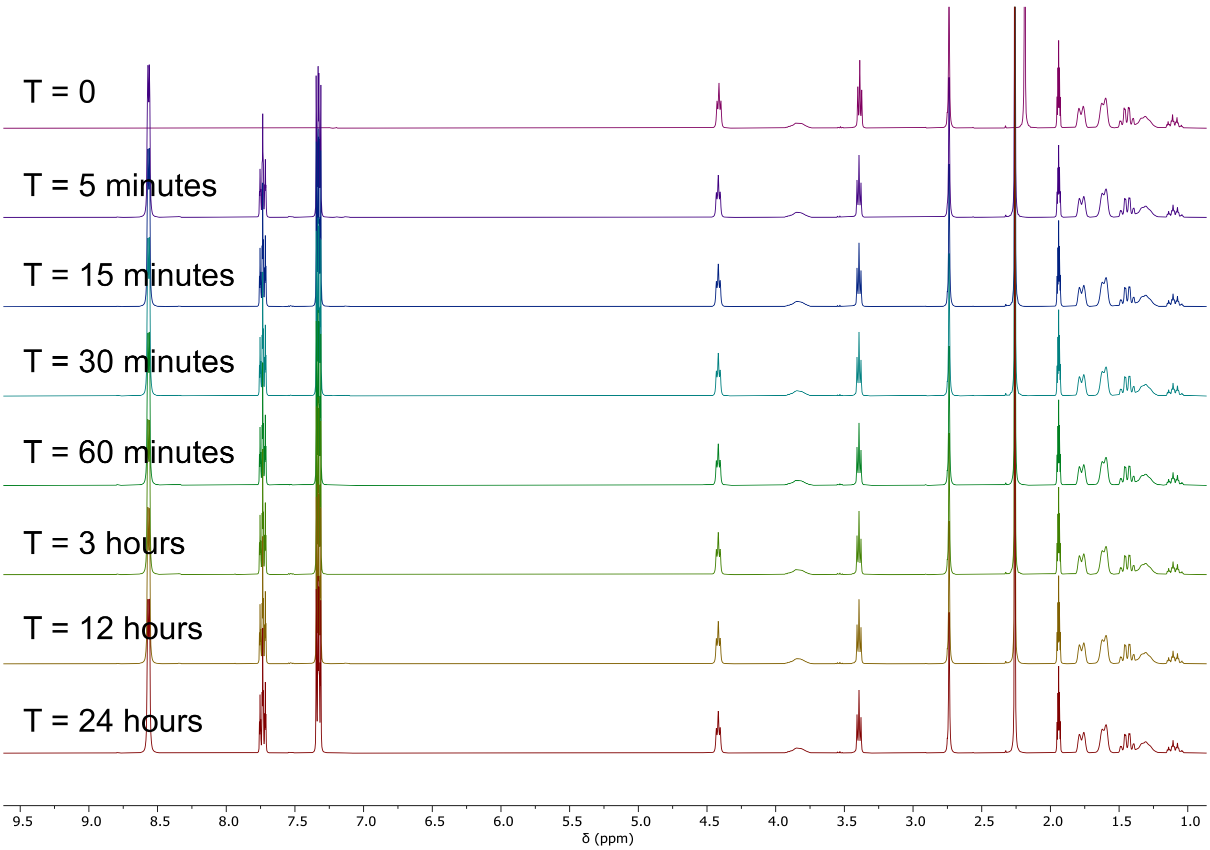

Figure S 11 ^{1}H NMR spectra recorded overtime following the addition of 5 molar equiv. of pyridine to a 10 mg mL^{-1} solution of model urethane **3** (400 MHz, MeCN-d_3). Descending from T=0 minutes to T=24 hours.

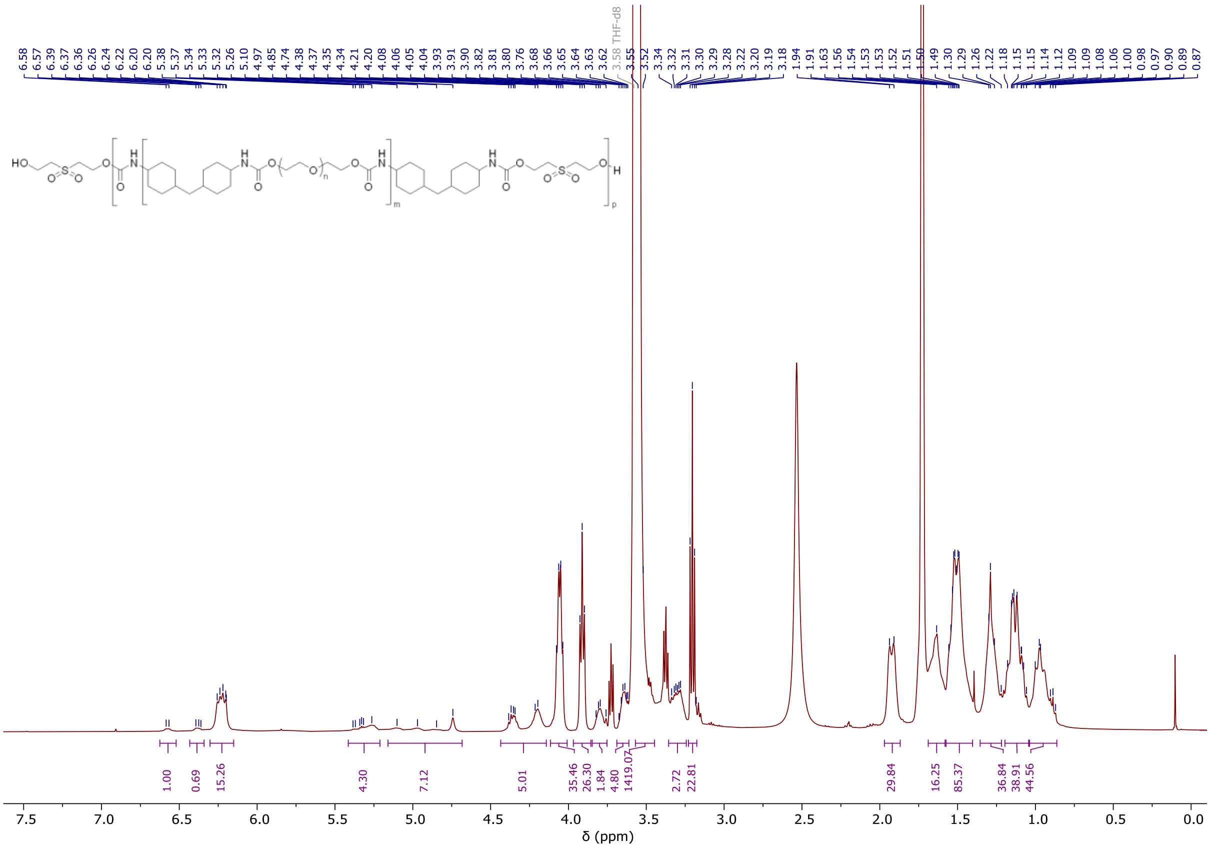

Figure S 12 ^{1}H NMR spectrum of **CEPU2** (400 MHz, THF-d_8, 298 K).

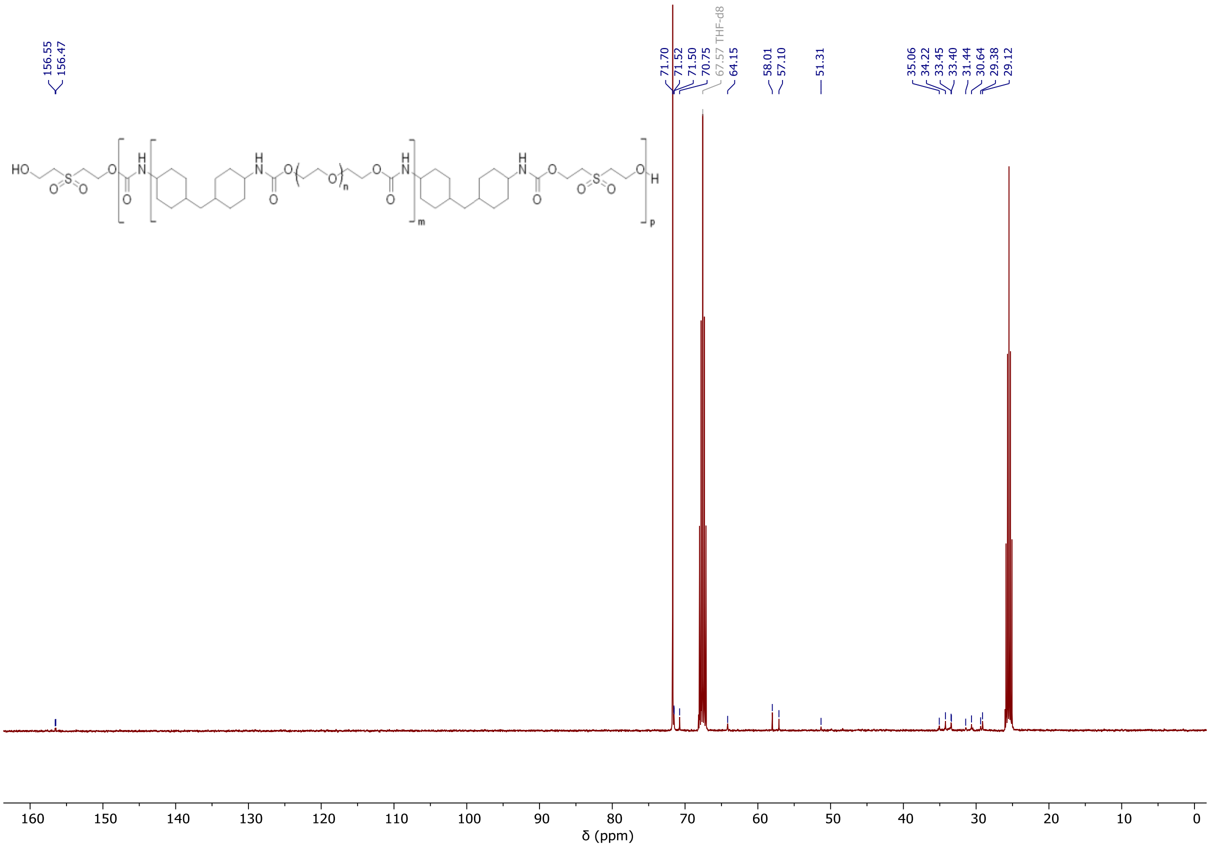

Figure S 13 ^{13}C {H} NMR spectrum of **CEPU2** (100 MHz, THF-d_8, 298 K).

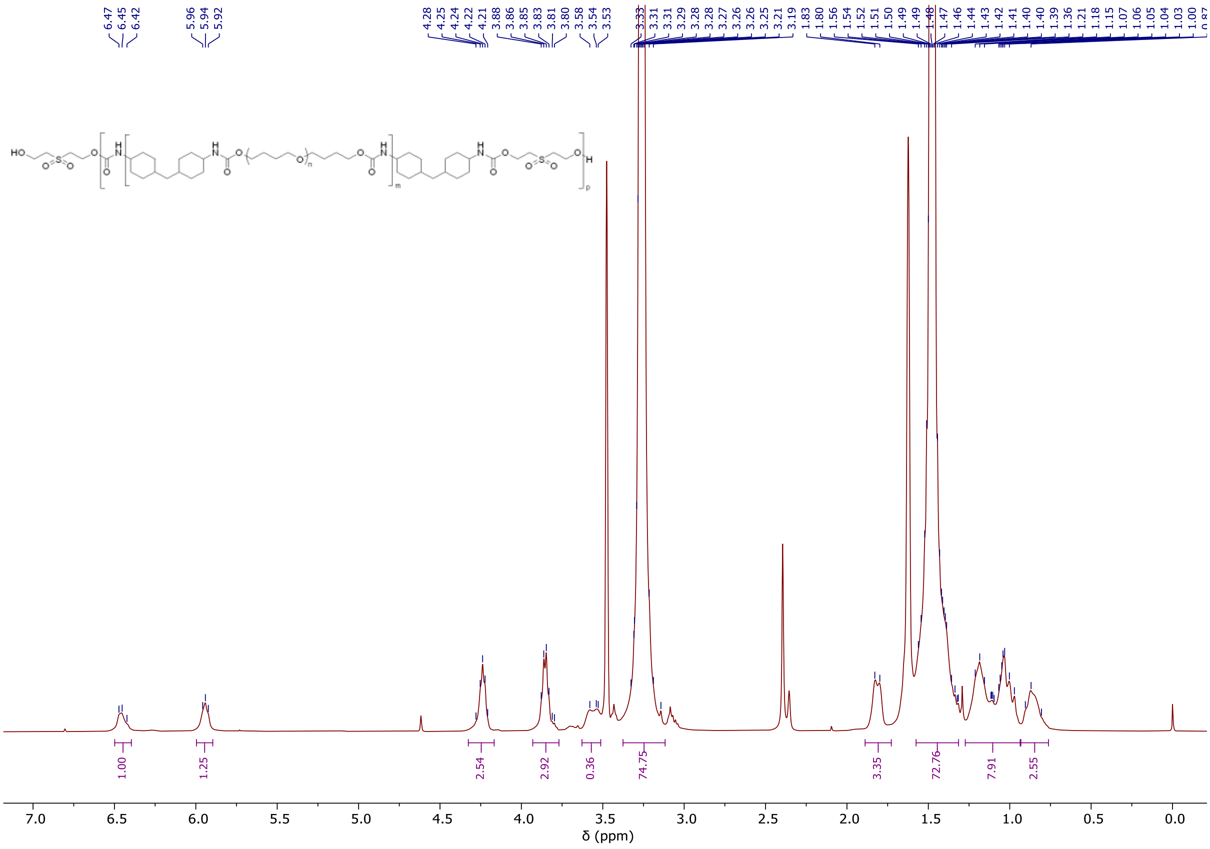

Figure S 14 ^{1}H NMR spectrum of **CEPU3** (400 MHz, THF-d_8, 298 K).

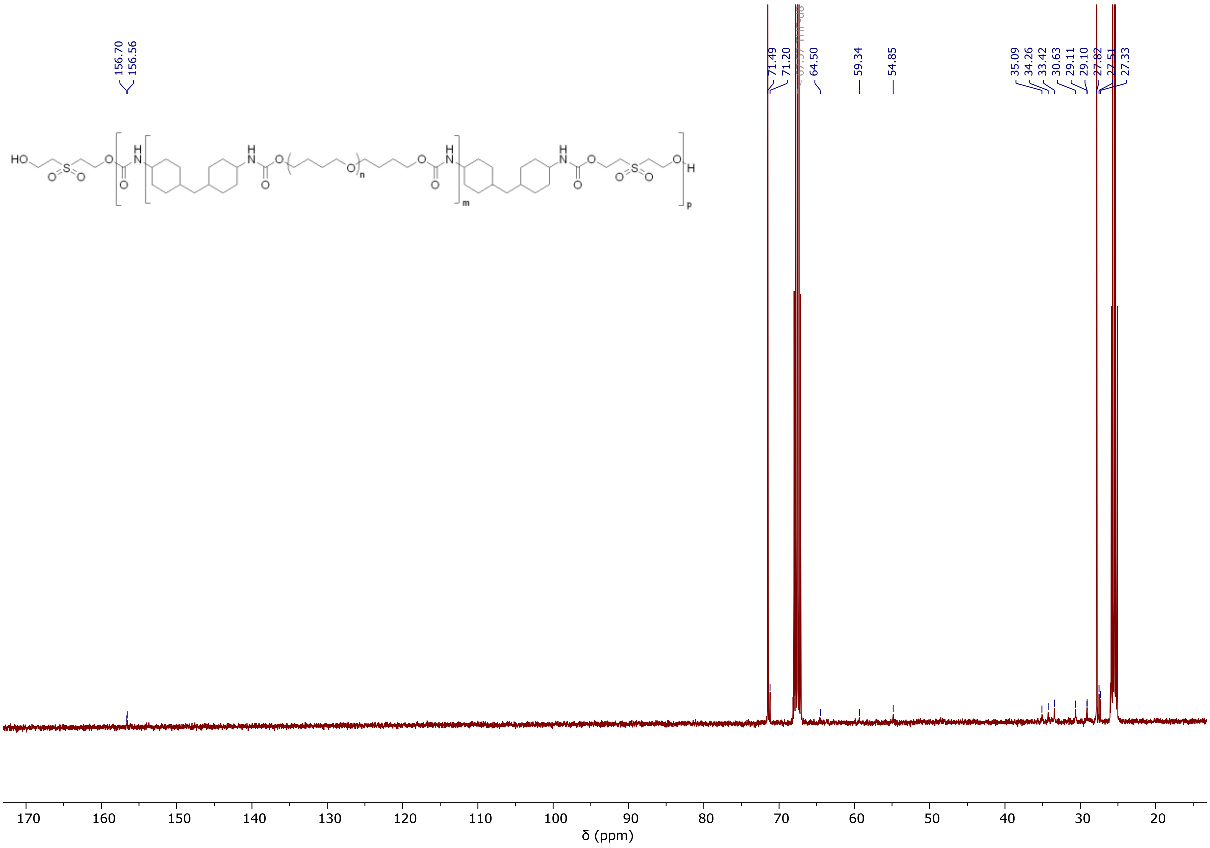

Figure S 15 ^{13}C {H} NMR spectrum of **CEPU3** (100 MHz, THF-d_8, 298 K).

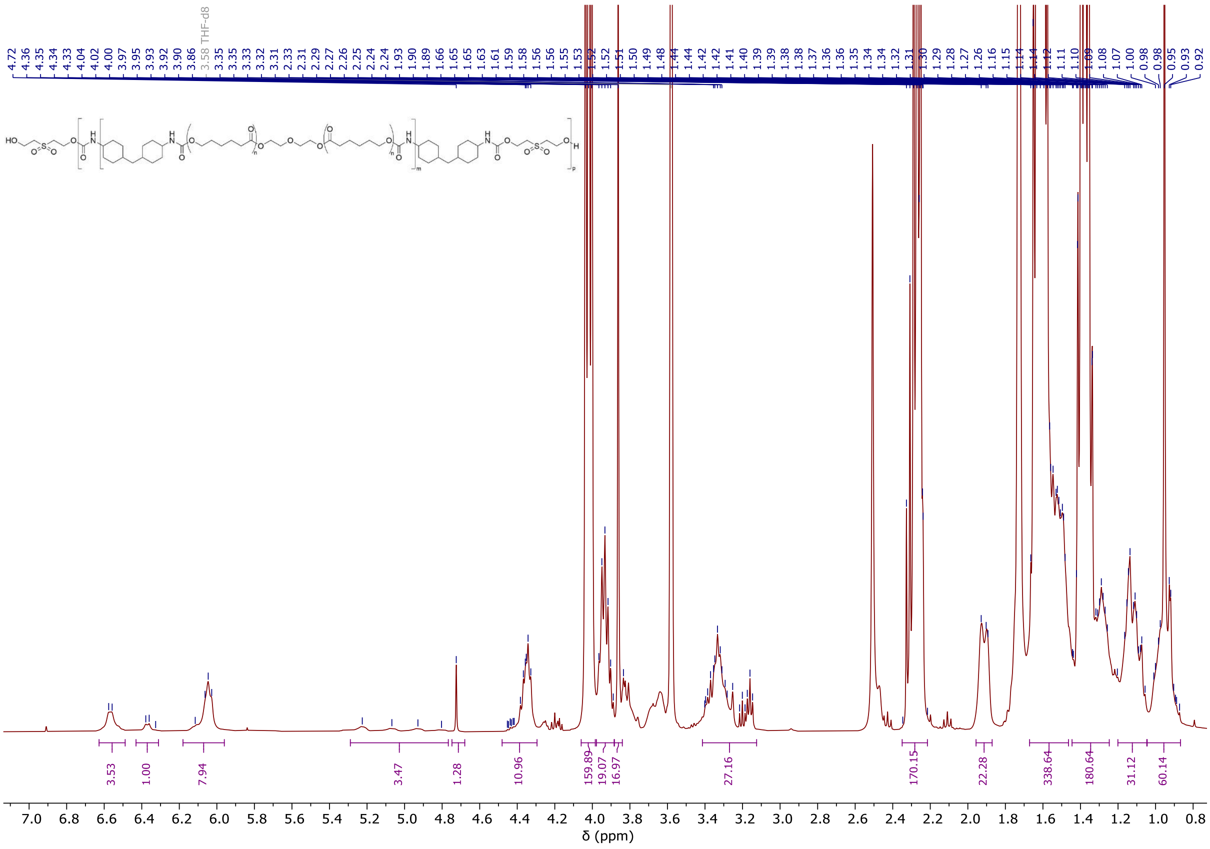

Figure S 16 ^{1}H NMR spectrum of **CEPU4** (400 MHz, THF-d_8, 298 K).

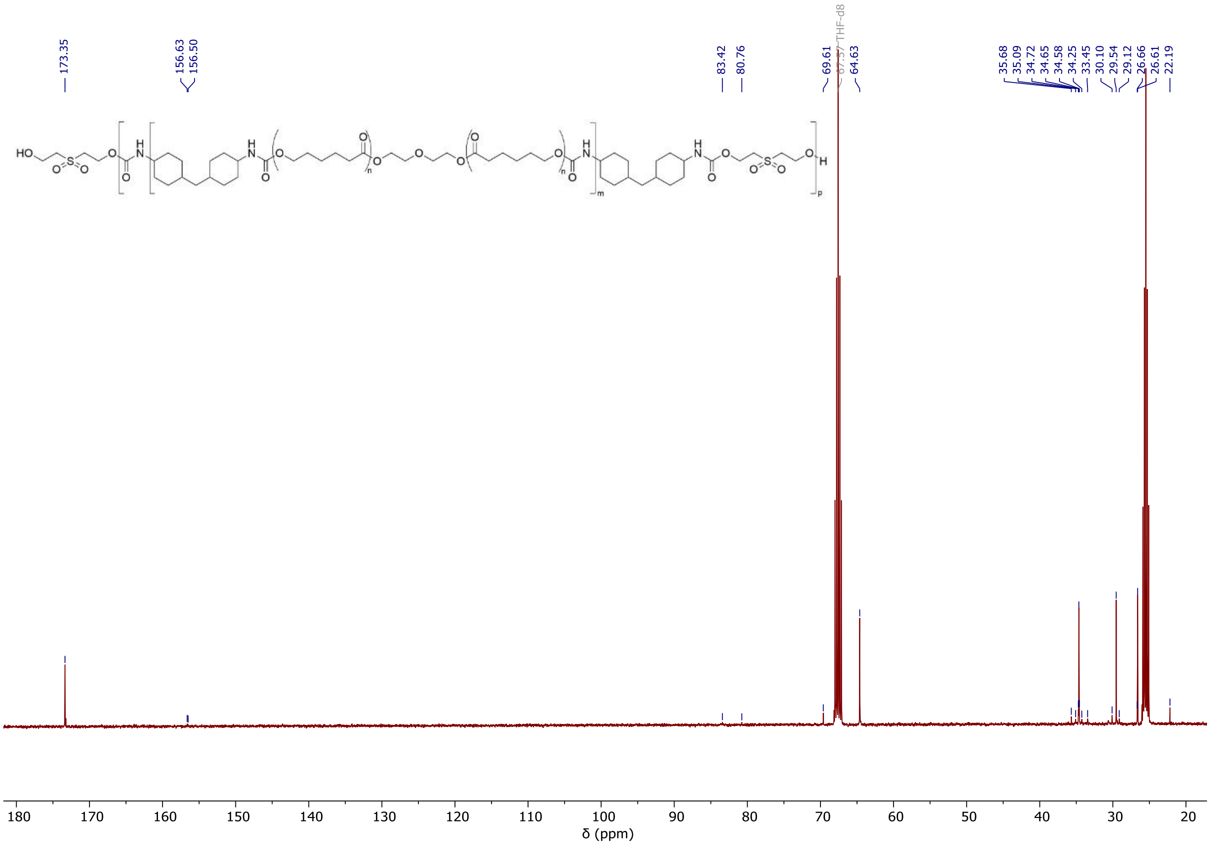

Figure S 17 ^{13}C {H} NMR spectrum of **CEPU4** (100 MHz, THF-d_8, 298 K).

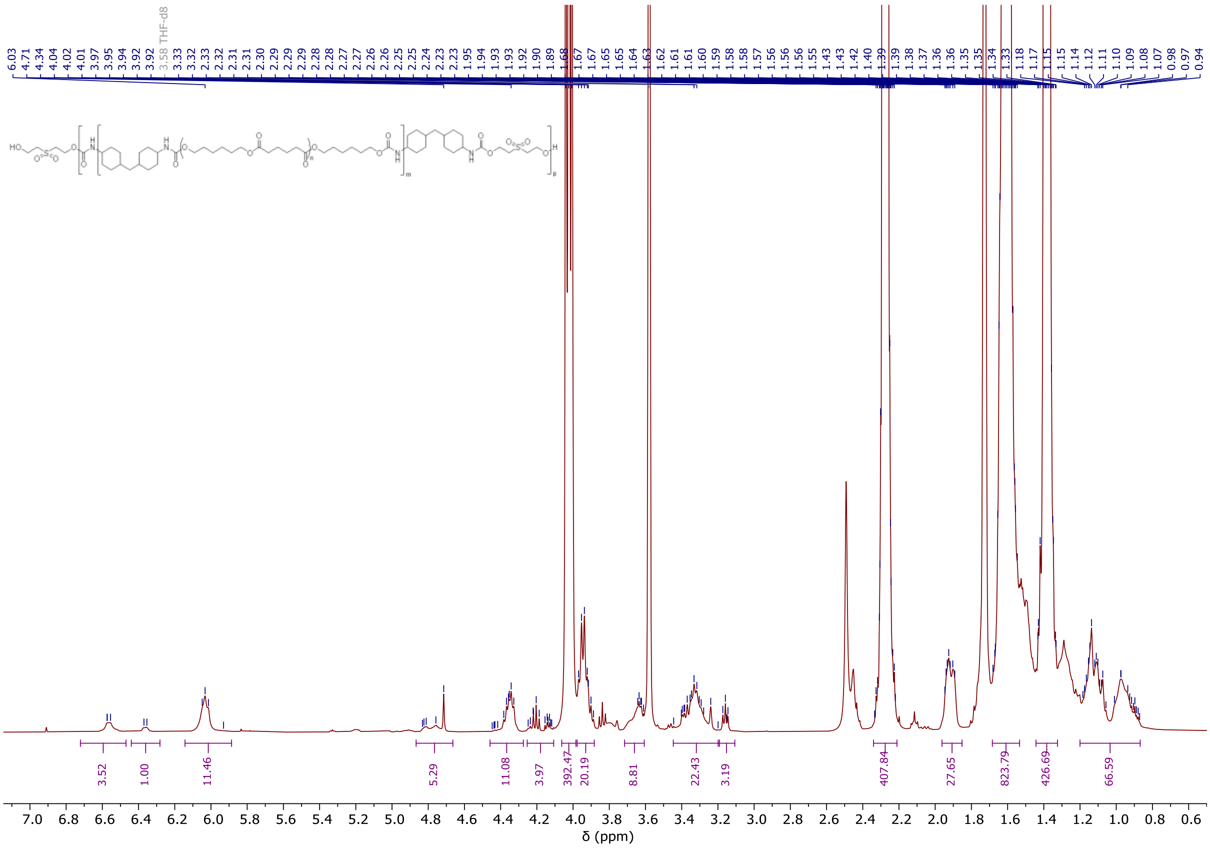

Figure S 18 ^{1}H NMR spectrum of **CEPU5** (400 MHz, THF-d_8, 298 K).

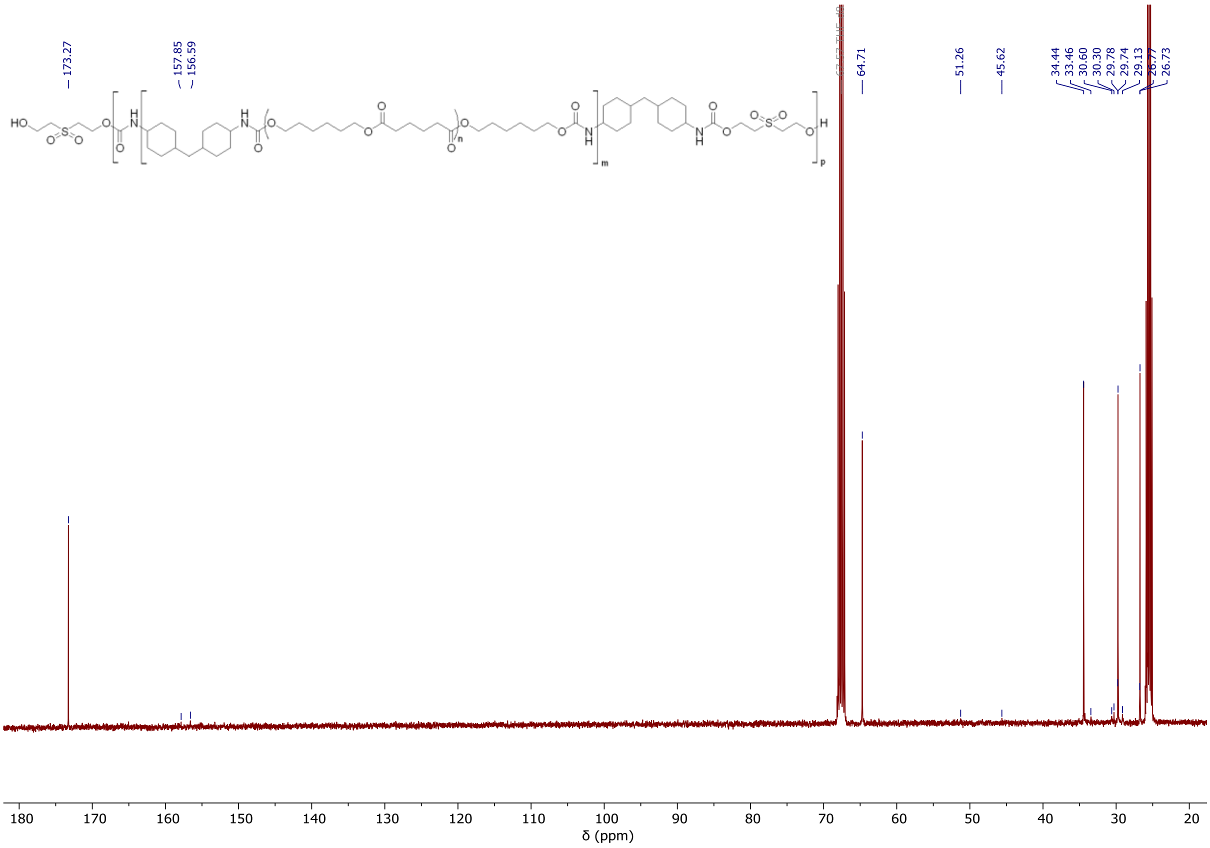

Figure S 19 ^{13}C {H} NMR spectrum of **CEPU5** (100 MHz, THF-d_8, 298 K).

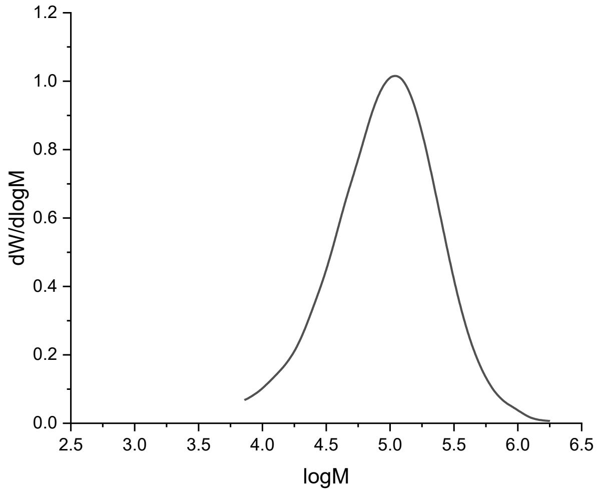

Figure S 20 GPC eluogram of **CEPU1** in THF.

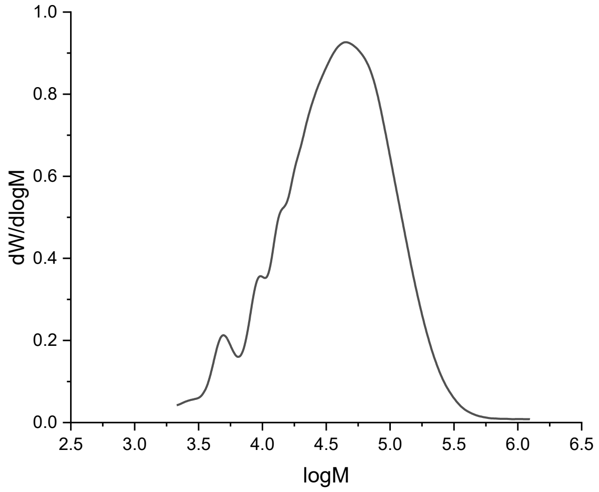

Figure S 21 GPC eluogram of **CEPU2** in THF.

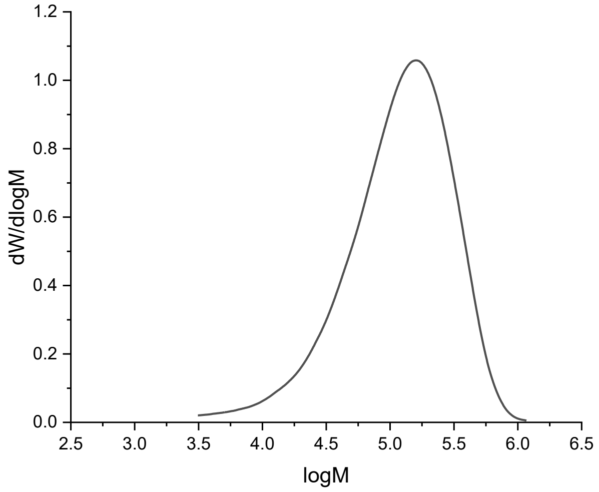

Figure S 22 GPC eluogram of **CEPU3** in THF.

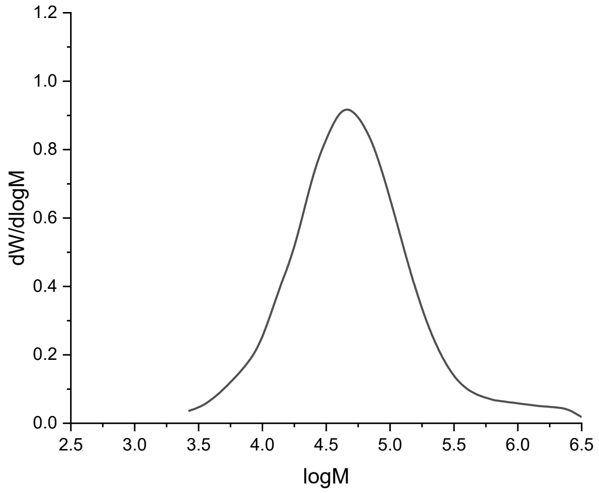

Figure S 23 GPC eluogram of **CEPU4** in THF.

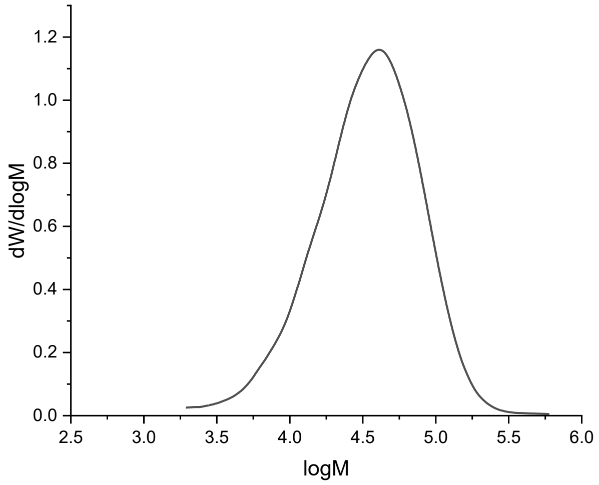

Figure S 24 GPC eluogram of **CEPU5** in THF.

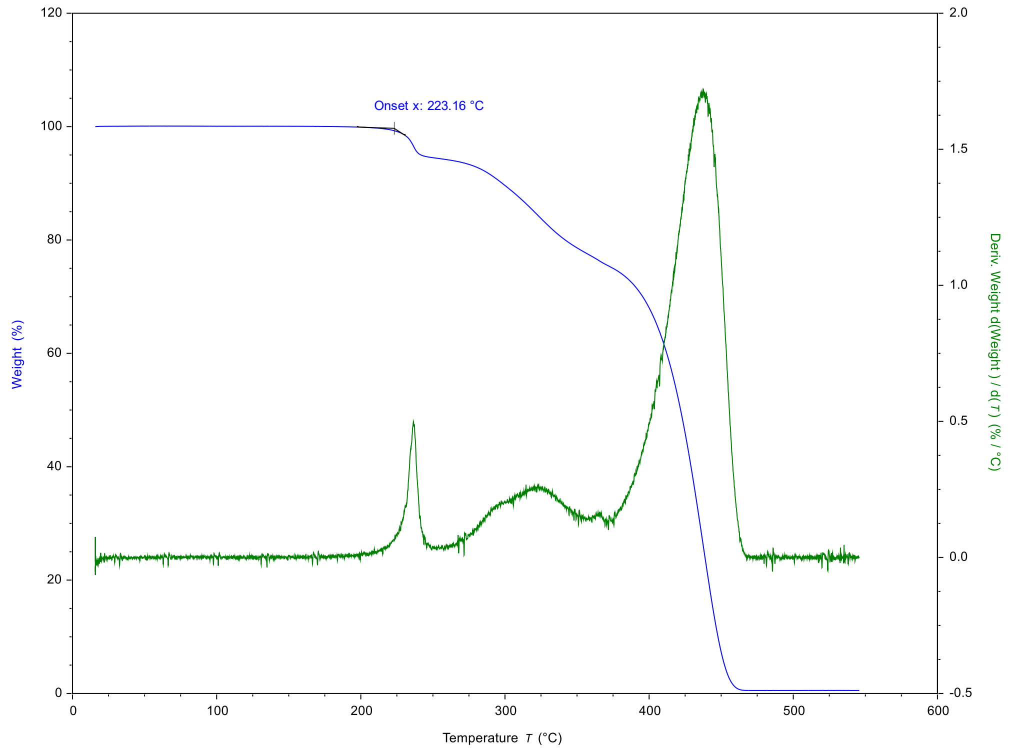

Figure S 25 TGA of **CEPU1** at 10 °C min^{-1}.

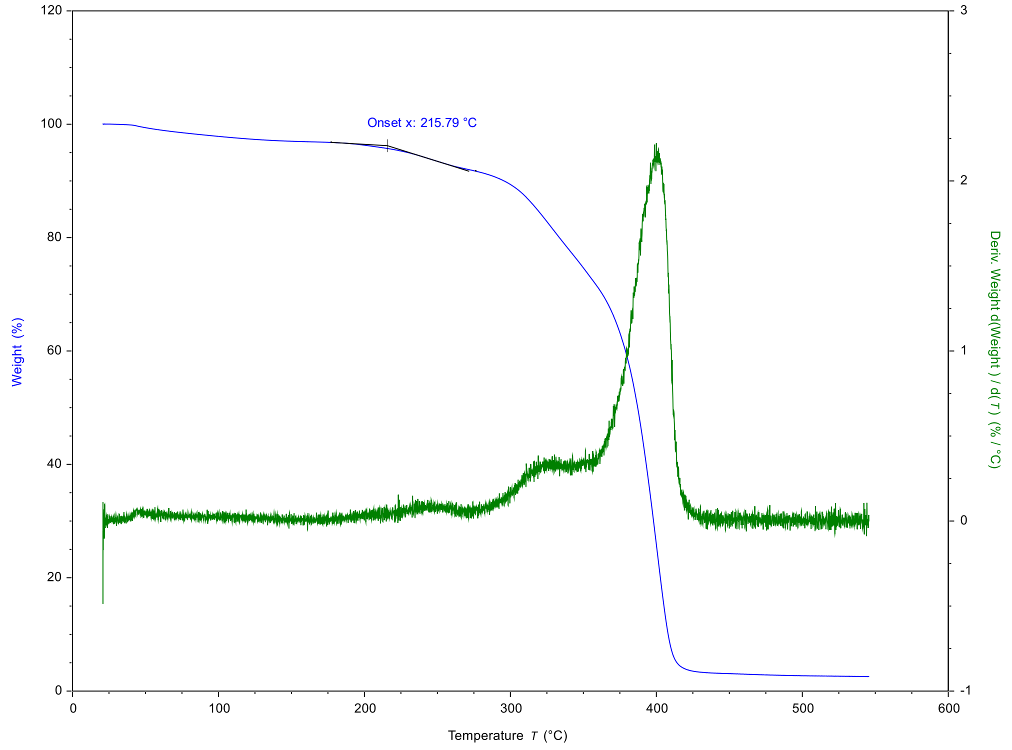

Figure S 26 TGA of **CEPU2** at 10 °C min^{-1}.

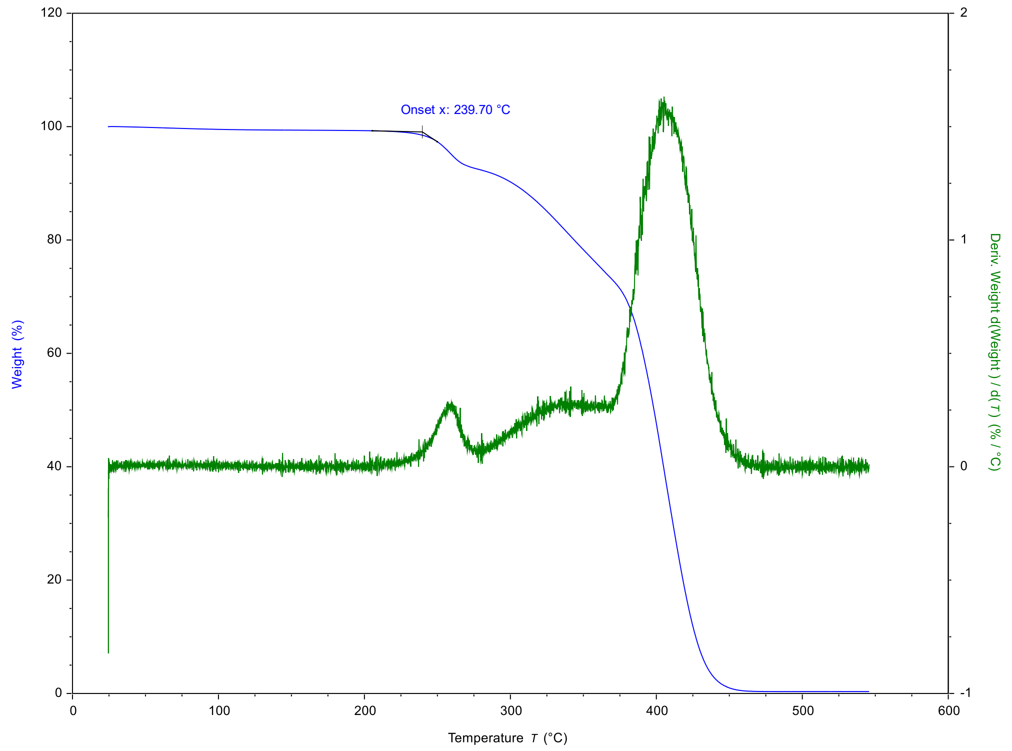

Figure S 27 TGA of **CEPU3** at 10 °C min^{-1}.

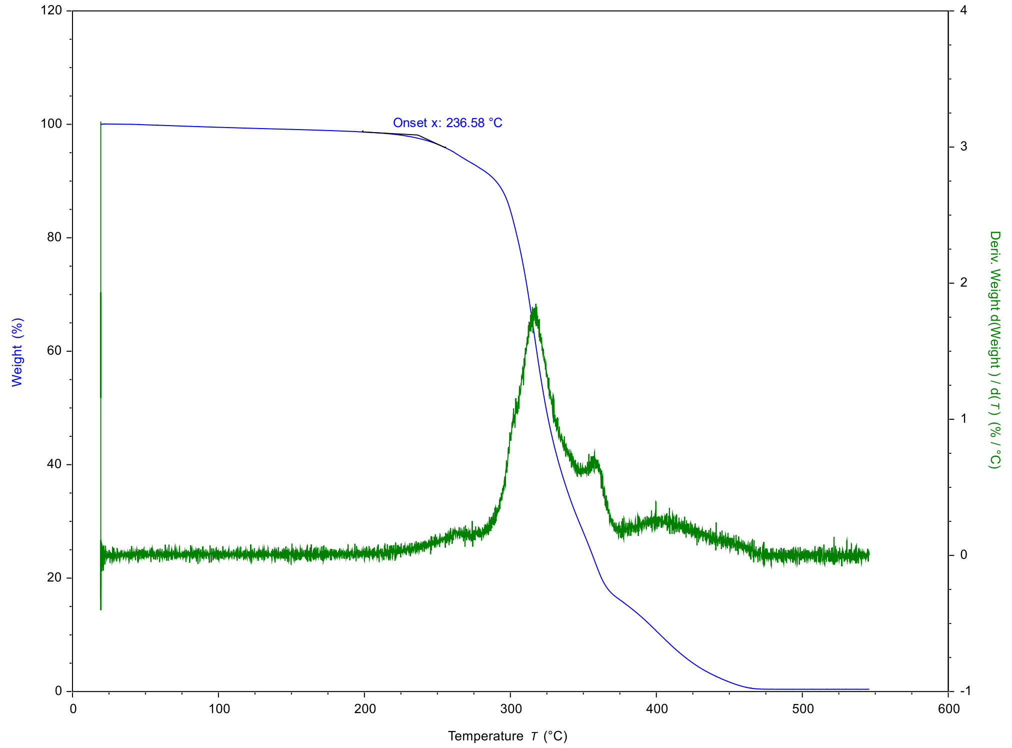

Figure S 28 TGA of **CEPU4** at 10 °C min^{-1}.

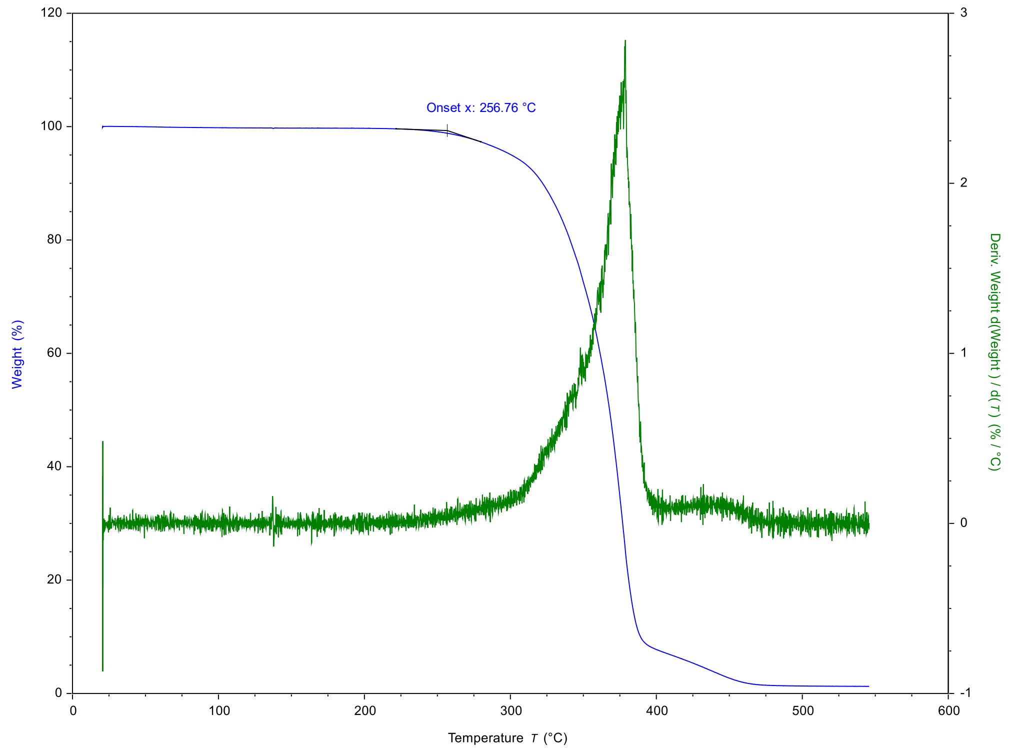

Figure S 29 TGA of **CEPU5** at 10 °C min^{-1}.

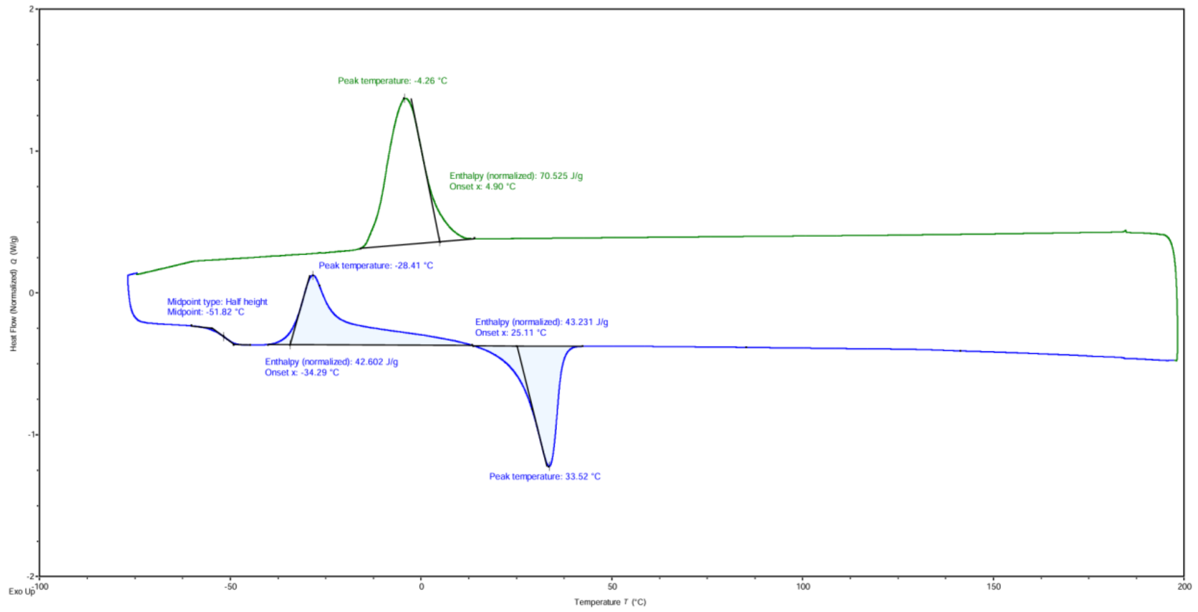

Figure S 30 DSC of **CEPU2** at 10 °C min^{-1}, showing the 2nd heating and cooling cycle.

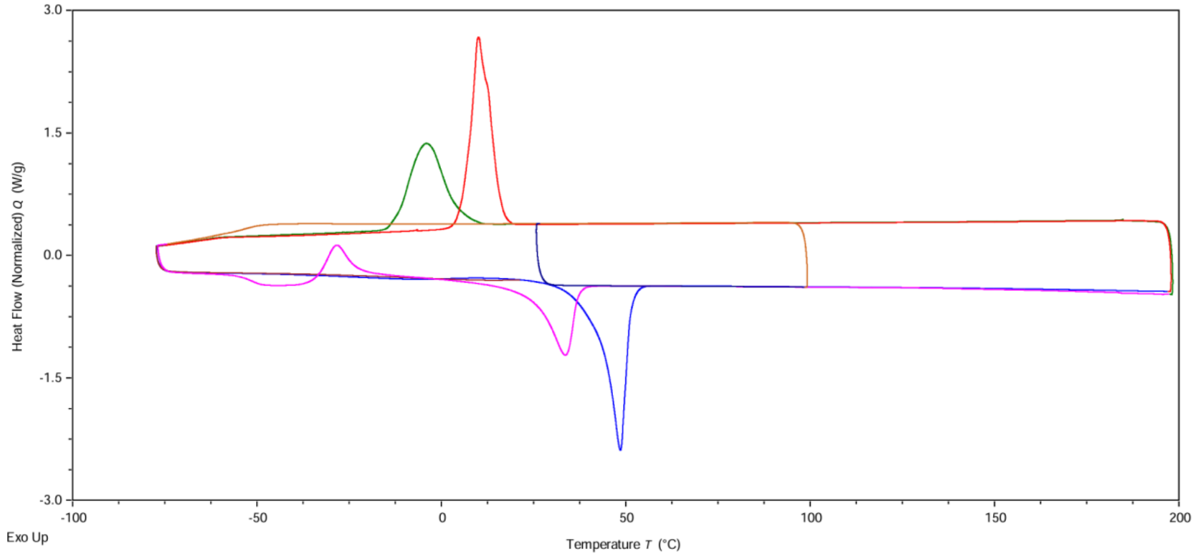

Figure S 31 DSC of **CEPU2** at 10 °C min^{-1}, showing the 1st, 2nd, and 3rd heating and cooling cycles.

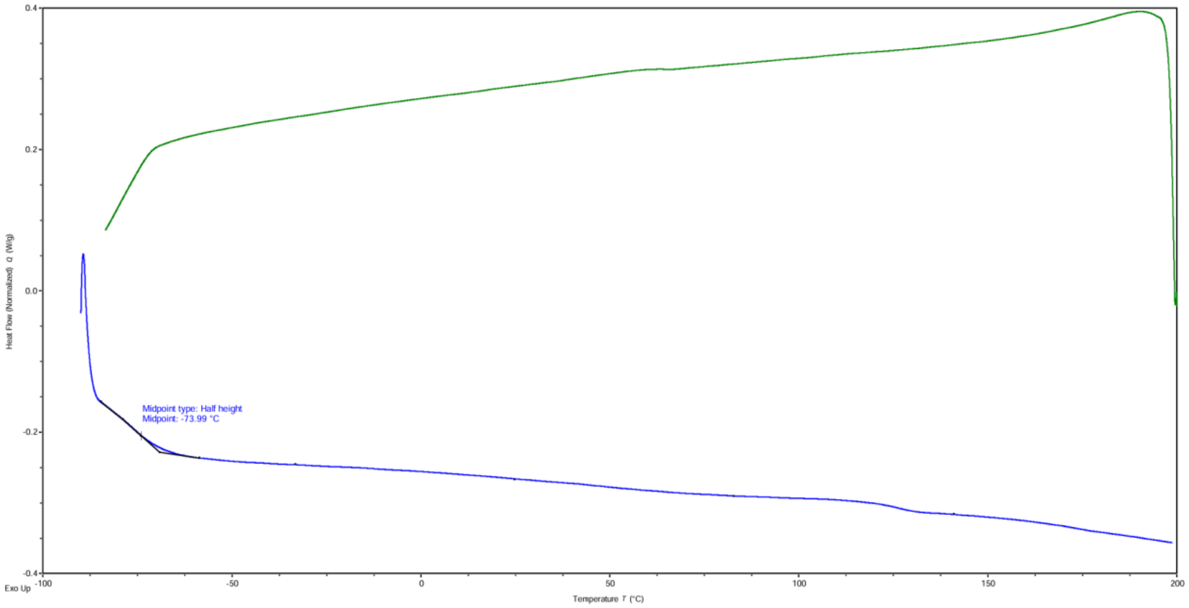

Figure S 32 DSC of **CEPU3** at 10 °C min^{-1}, showing the 2ndheating and cooling cycle.

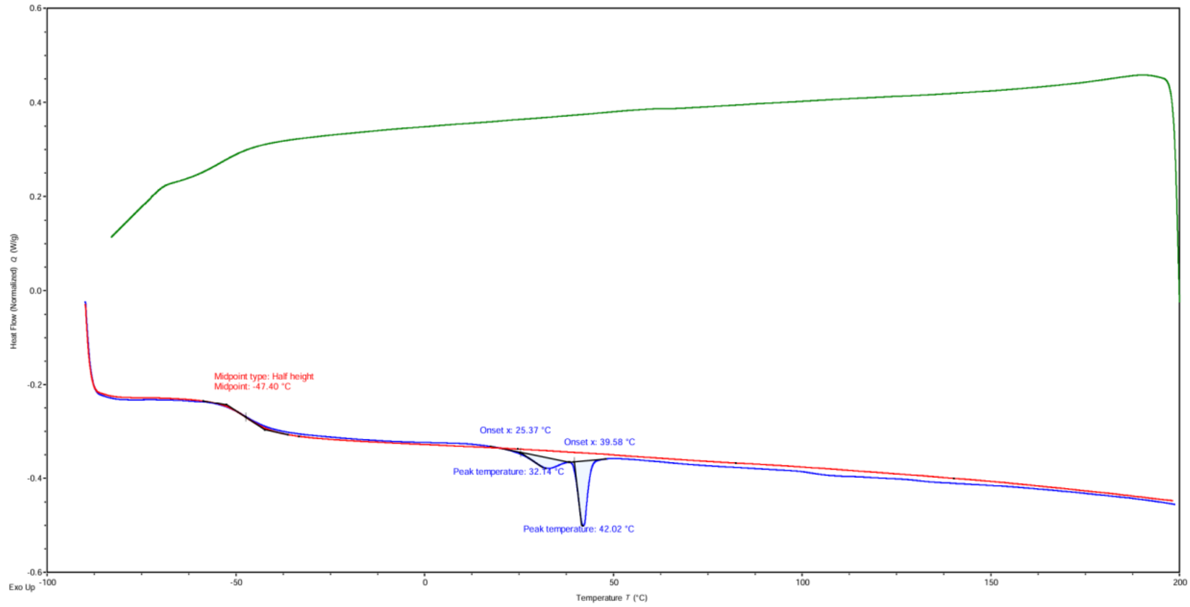

Figure S 33 DSC of **CEPU4** at 10 °C min^{-1}, showing the 1st and 2nd heating and cooling cycle.

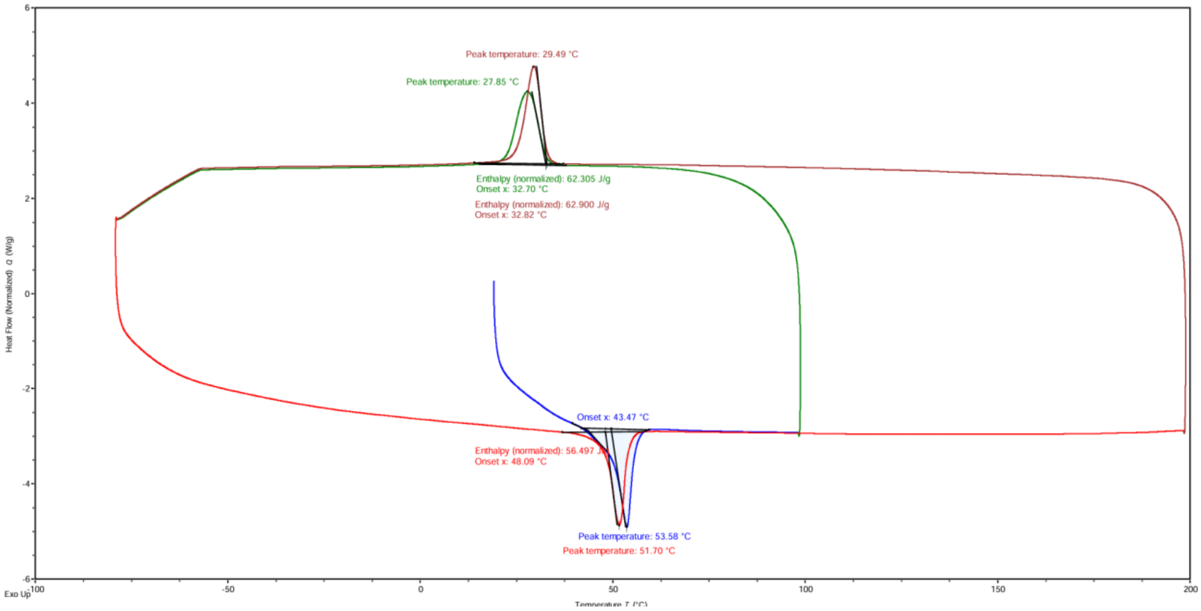

Figure S 34 DSC of **CEPU5** at 10 °C min^{-1}, showing the 1st and 2nd heating and cooling cycles.

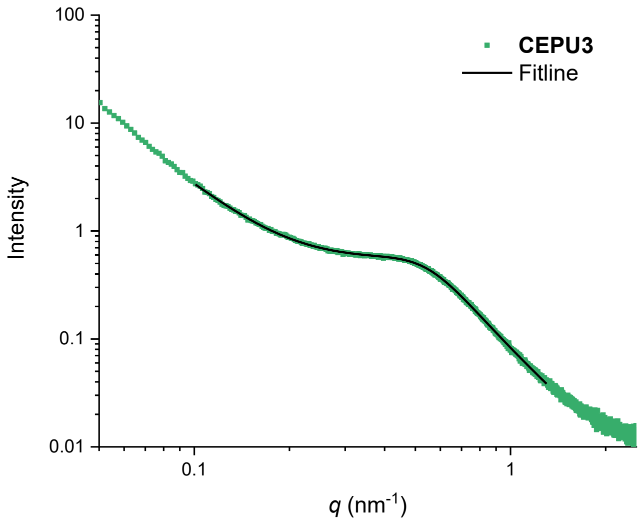

Figure S 35 SAXS profile of **CEPU3** and corresponding fit line at 20 °C.

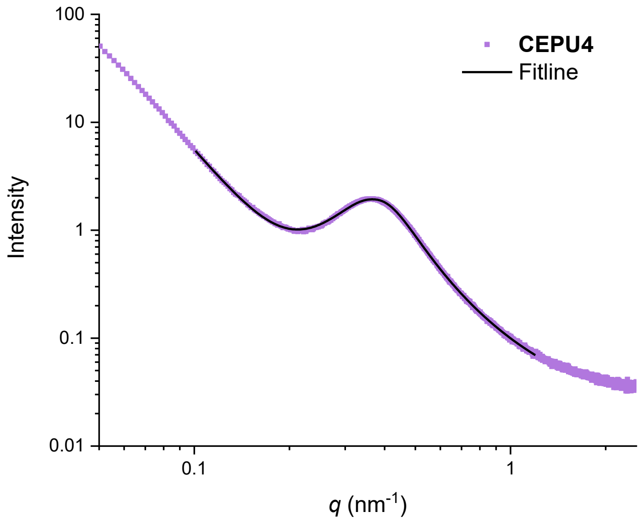

Figure S 36 SAXS profile of **CEPU4** and corresponding fit line at 20 °C.

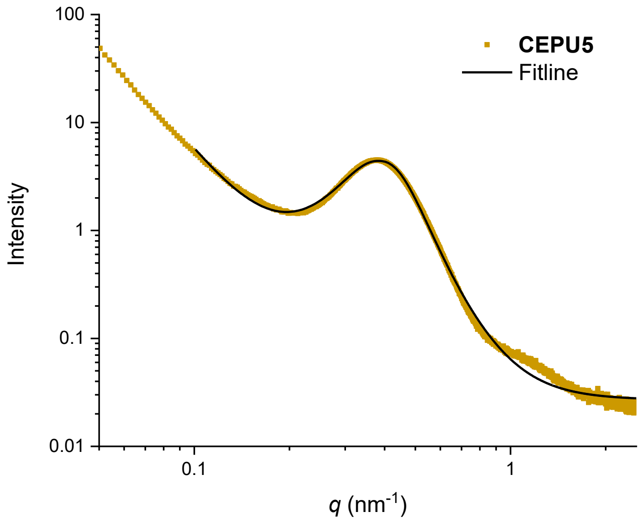

Figure S 37 SAXS profile of **CEPU5** and corresponding fit line at 20 °C.

Table S 1 q_{max} and corresponding *d*-spacing for **CEPU1-CEPU5** at 20 °C.

CEPU	q_{max} (nm^{-1})	*d*-spacing (nm)
**CEPU2**	-	-
**CEPU3**	0.44	14.3
**CEPU4**	0.37	17.0
**CEPU5**	0.38	16.5

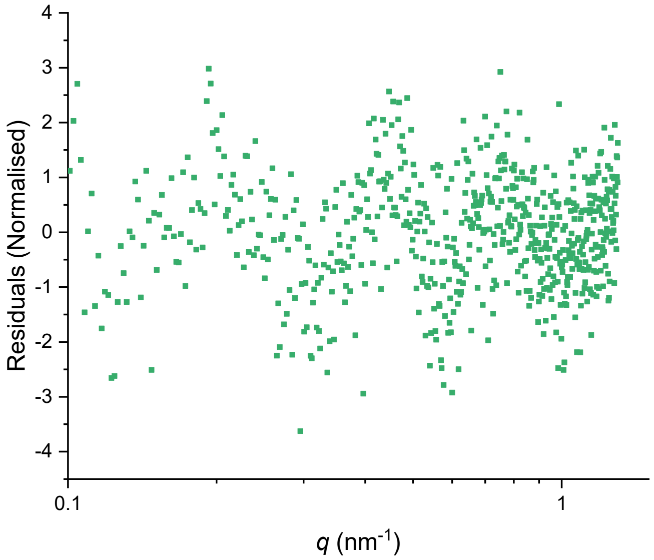

Figure S 38 SAXS fitting residuals for **CPEU3** at 20 °C.

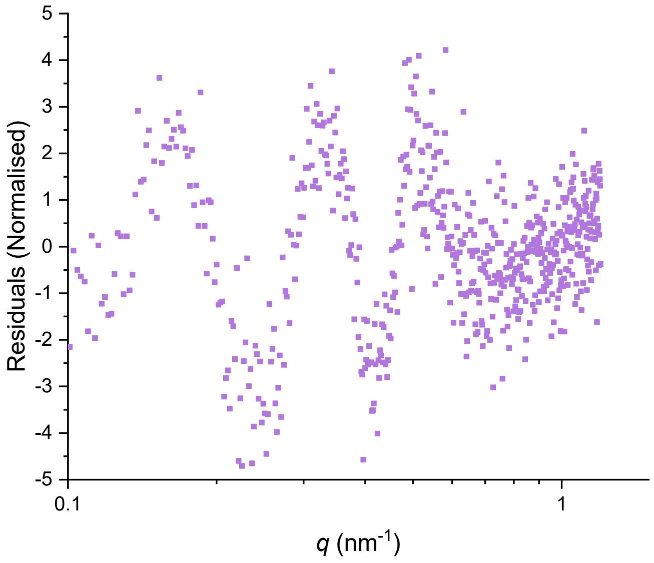

Figure S 39 SAXS fitting residuals for **CPEU4** at 20 °C.

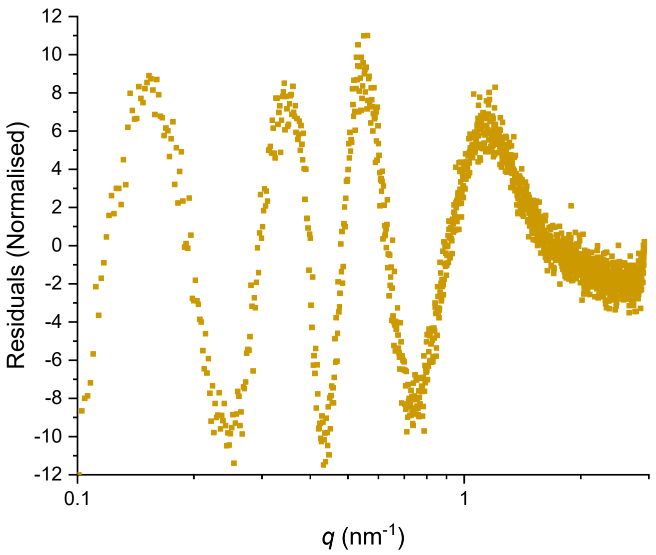

Figure S 40 SAXS fitting residuals for **CPEU5** at 20 °C.

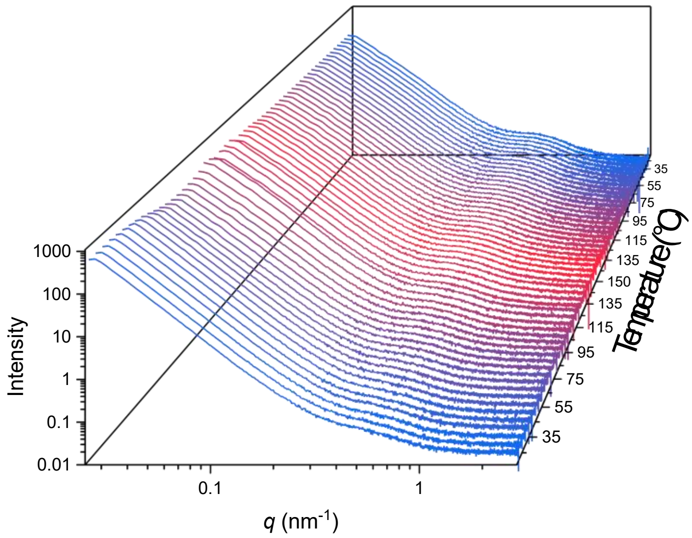

Figure S 41 VT-SAXS profiles of **CEPU2** recorded at 5 °C intervals from 20 °C to 150 °C at a heating and cooling rate of 10 °C min^{-1}.

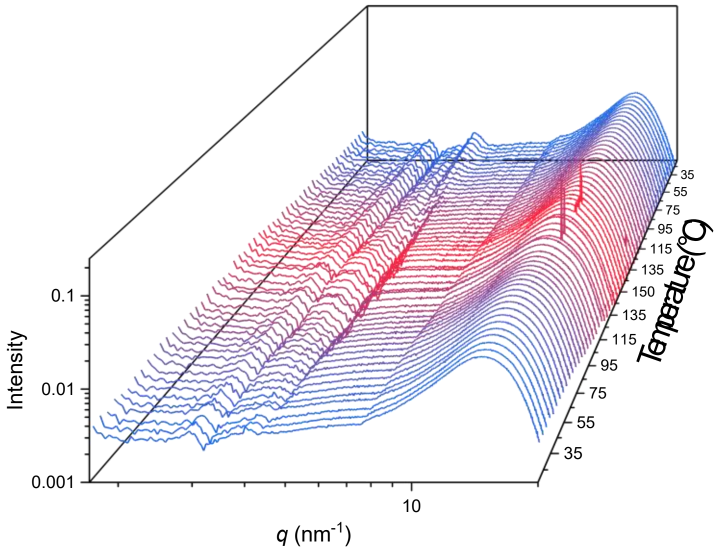

Figure S 42 VT-WAXS profiles of **CEPU2** recorded at 5 °C intervals from 20 °C to 150 °C at a heating and cooling rate of 10 °C min^{-1}.

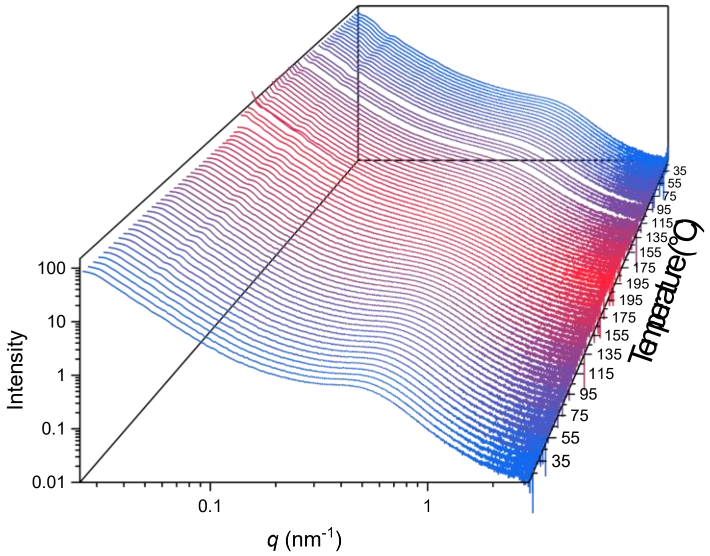

Figure S 43 VT-SAXS profiles of **CEPU3** recorded at 5 °C intervals from 20 °C to 200 °C at a heating and cooling rate of 10 °C min^{-1}.

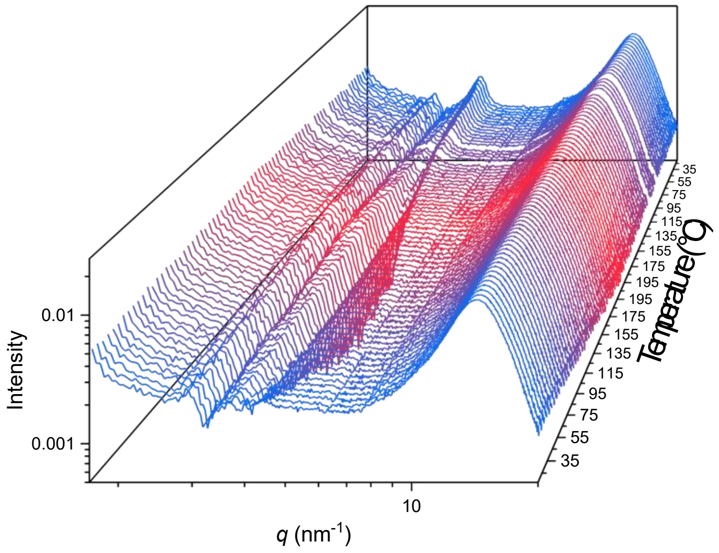

Figure S 44 VT-WAXS profiles of **CEPU3** recorded at 5 °C intervals from 20 °C to 200 °C at a heating and cooling rate of 10 °C min^{-1}.

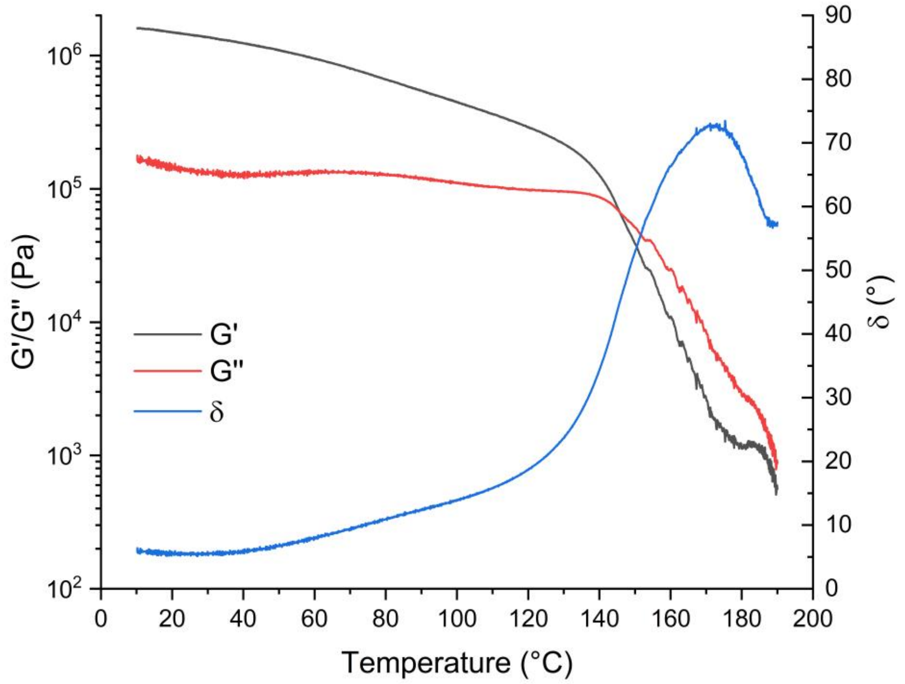

Figure S 45 Temperature sweep analysis of **CEPU1** using a normal force of 1 N and a frequency of 1 Hz.

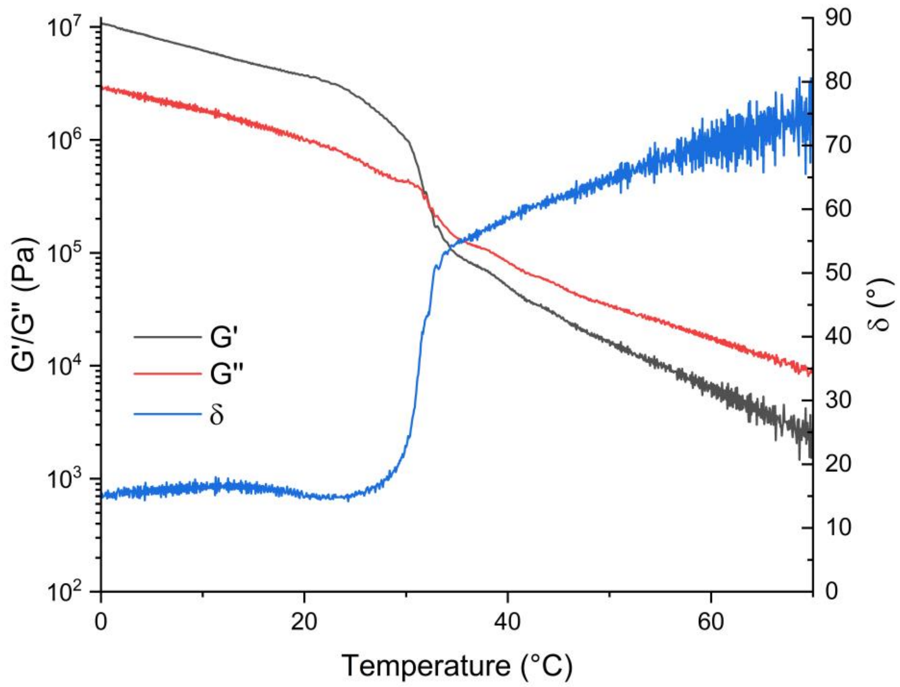

Figure S 46 Temperature sweep analysis of **CEPU2** using a normal force of 1 N and a frequency of 1 Hz.

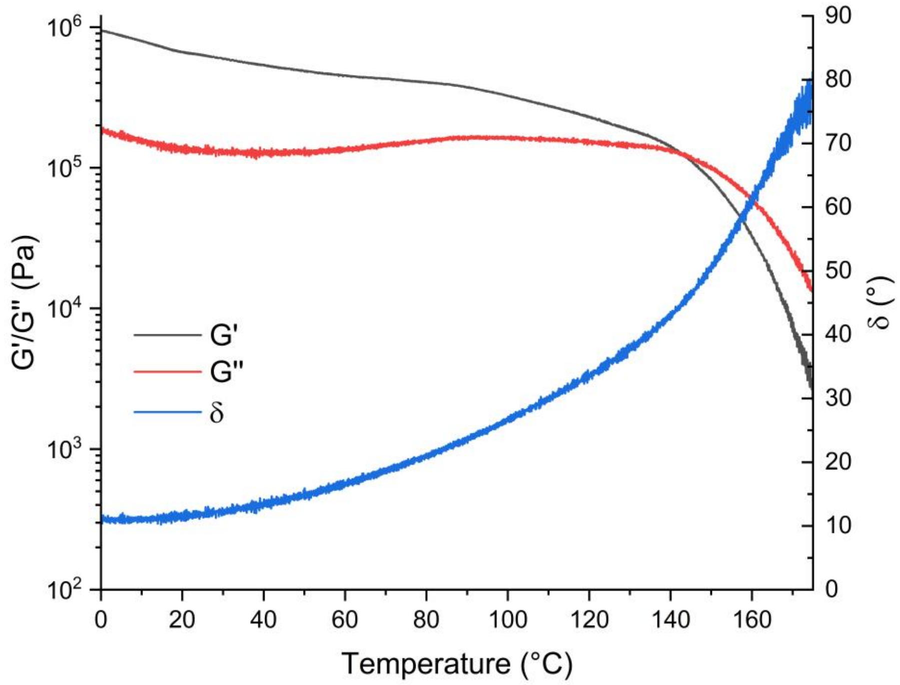

Figure S 47 Temperature sweep analysis of **CEPU3** using a normal force of 1 N and a frequency of 1 Hz.

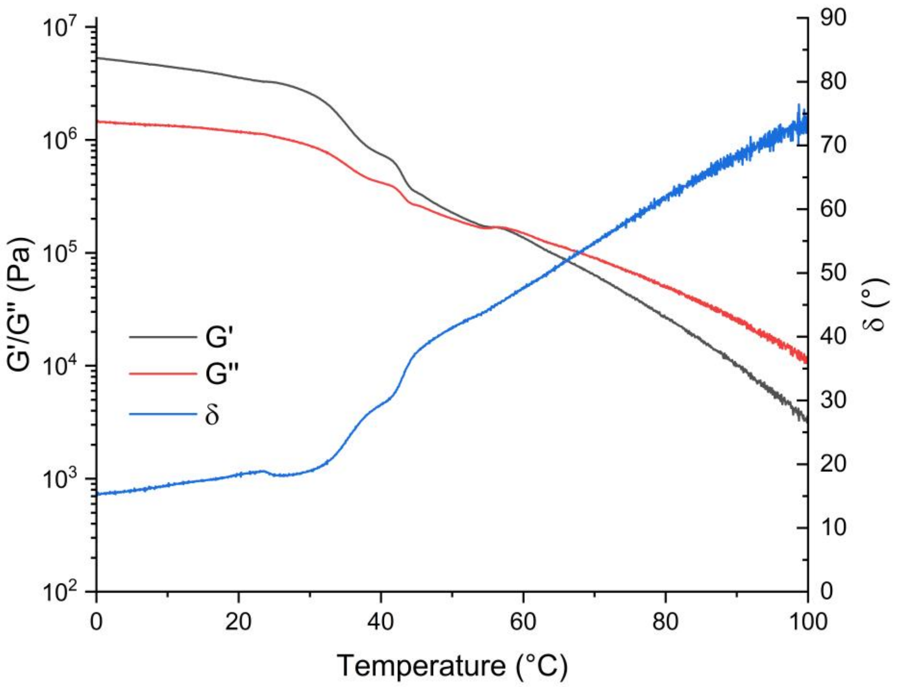

Figure S 48 Temperature sweep analysis of **CEPU4** using a normal force of 1 N and a frequency of 1 Hz.

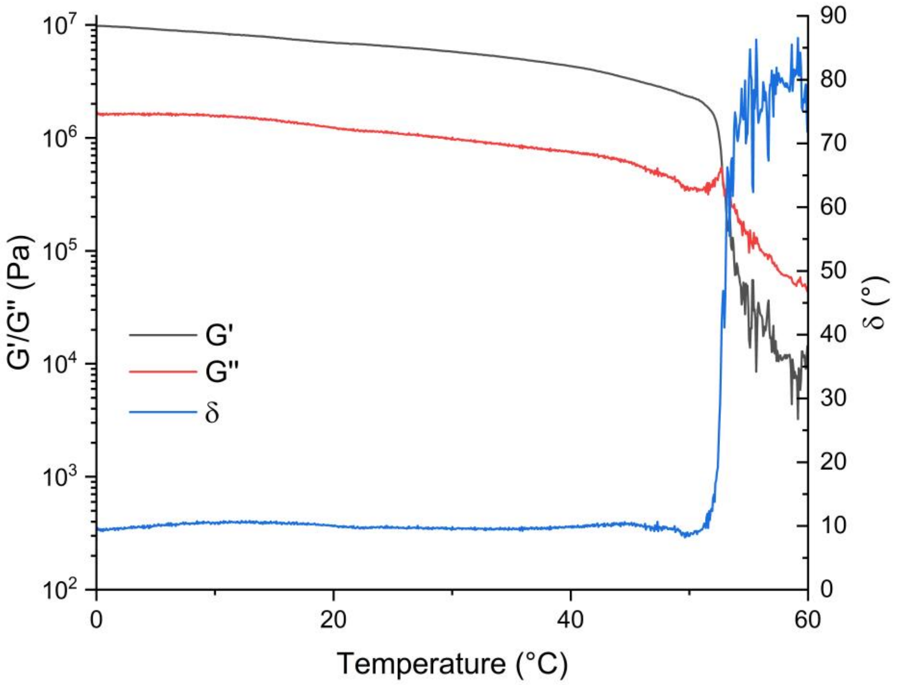

Figure S 49 Temperature sweep analysis of **CEPU5** using a normal force of 1 N and a frequency of 1 Hz.

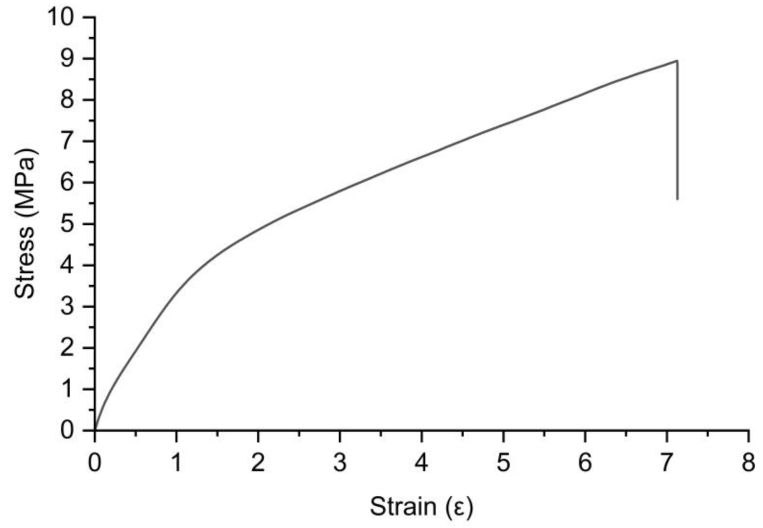

Figure S 50 Representative stress-strain curves for **CEPU1**.

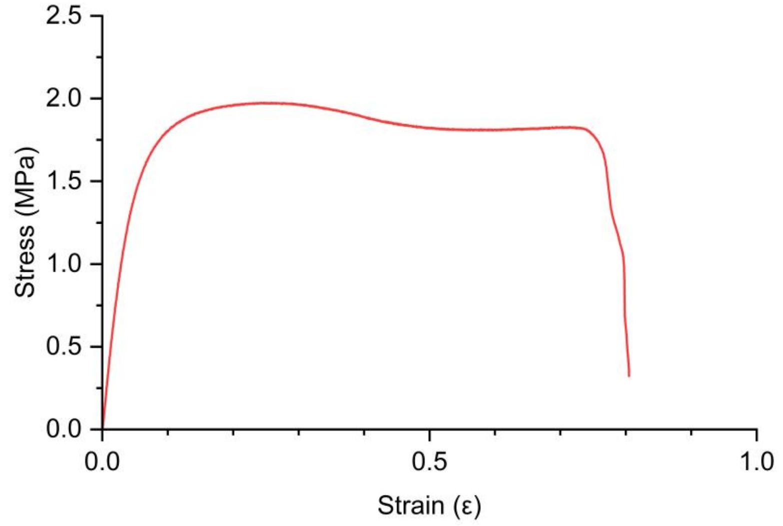

Figure S 51 Representative stress-strain curves for **CEPU2**.

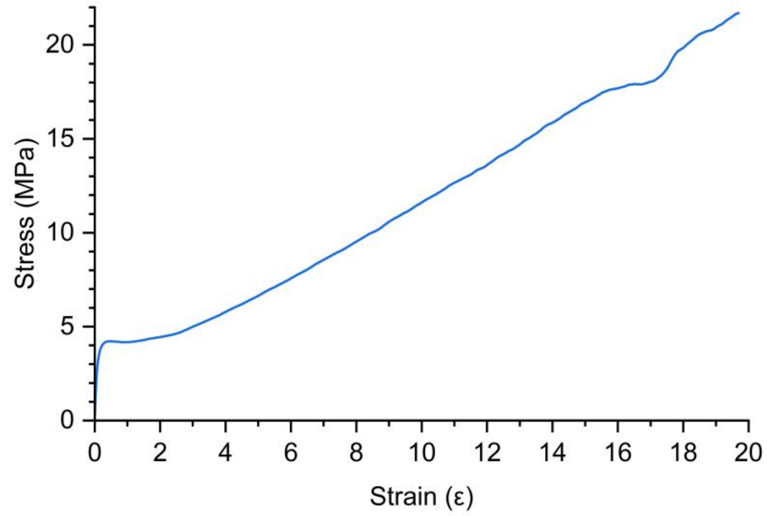

Figure S 52 Representative stress-strain curves for **CEPU3**.

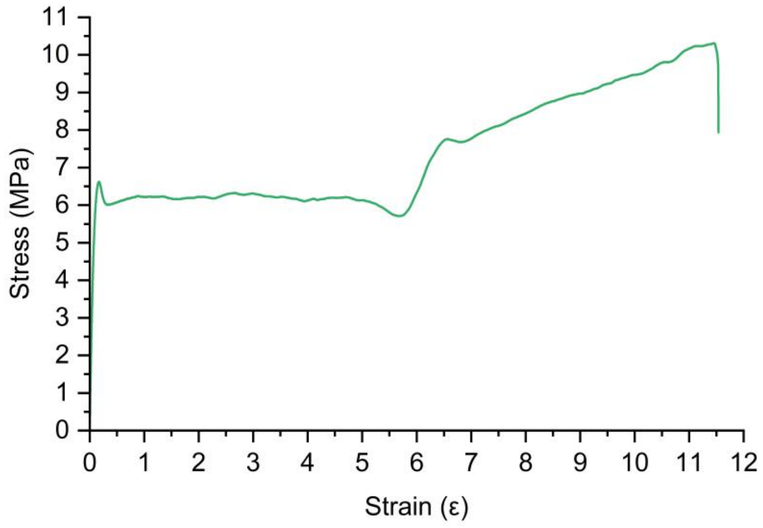

Figure S 53 Representative stress-strain curves for **CEPU4**.

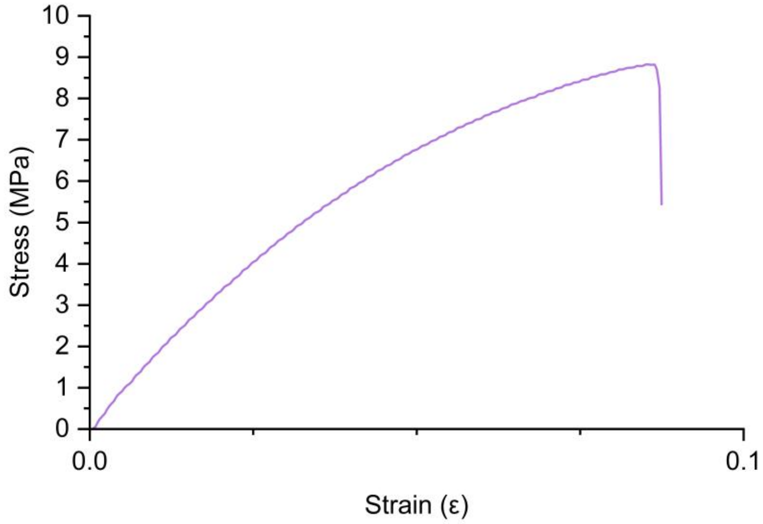

Figure S 54 Representative stress-strain curves for **CEPU5**.

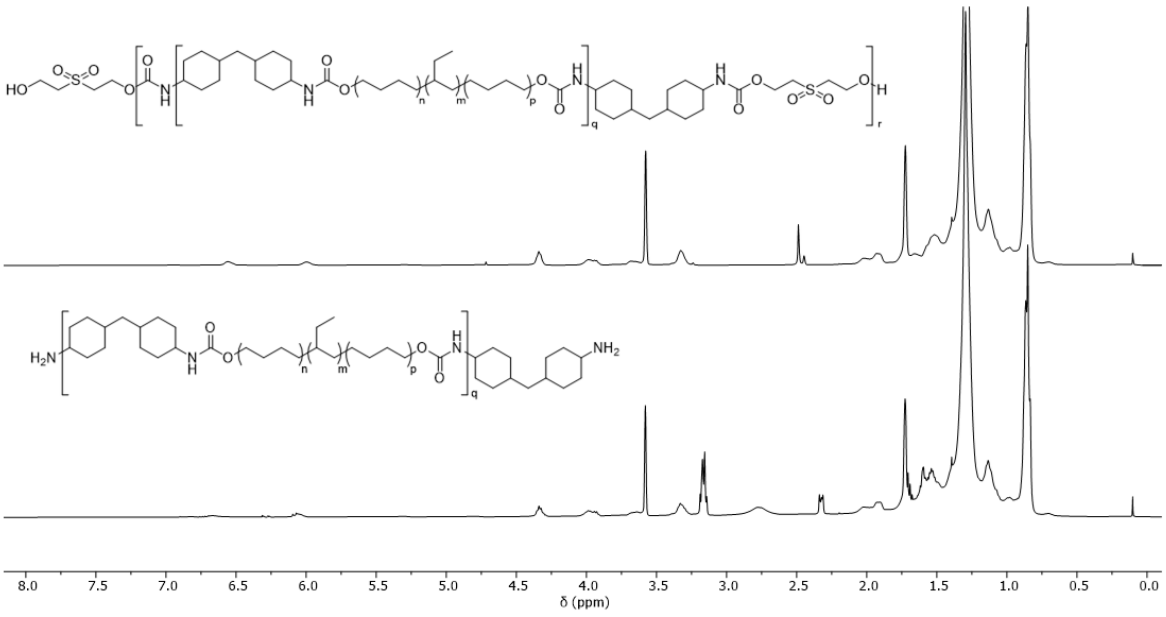

Figure S 55 ^{1}H NMR spectra showing the solution degradation of **CEPU1** with DBU, (400 MHz, THF-d_8). Top spectrum shows the pristine CEPU, bottom spectrum shows the degraded CEPU.

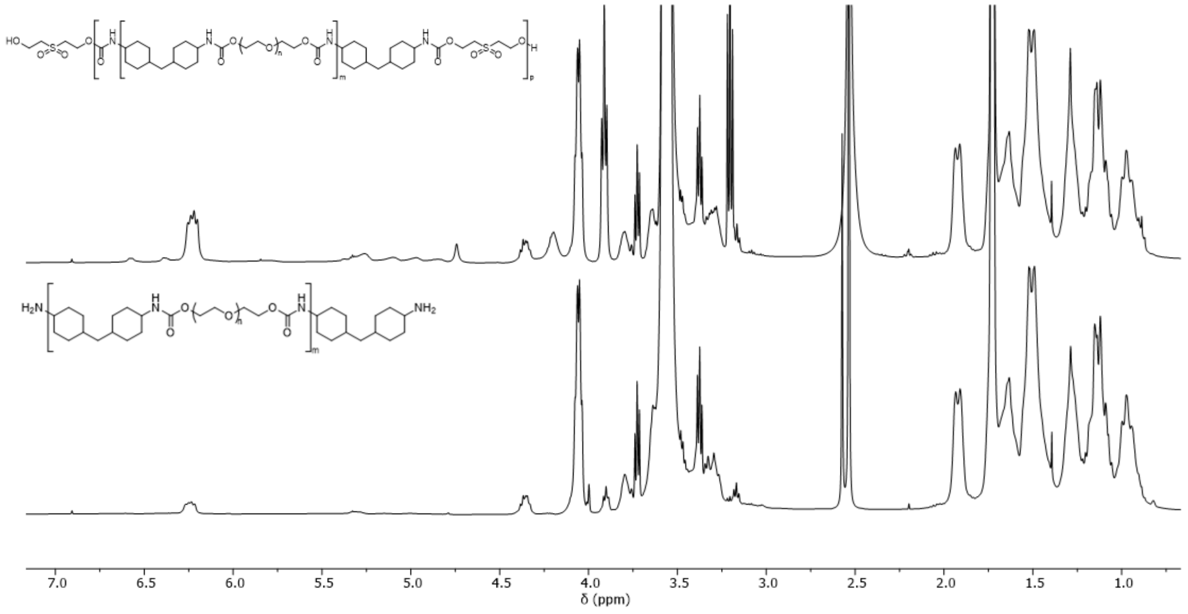

Figure S 56 ^{1}H NMR spectra showing the solution degradation of **CEPU2** with 40 wt.% NaOD in D_2O, (400 MHz, THF-d_8). Top spectrum shows the pristine CEPU, bottom spectrum shows the degraded CEPU.

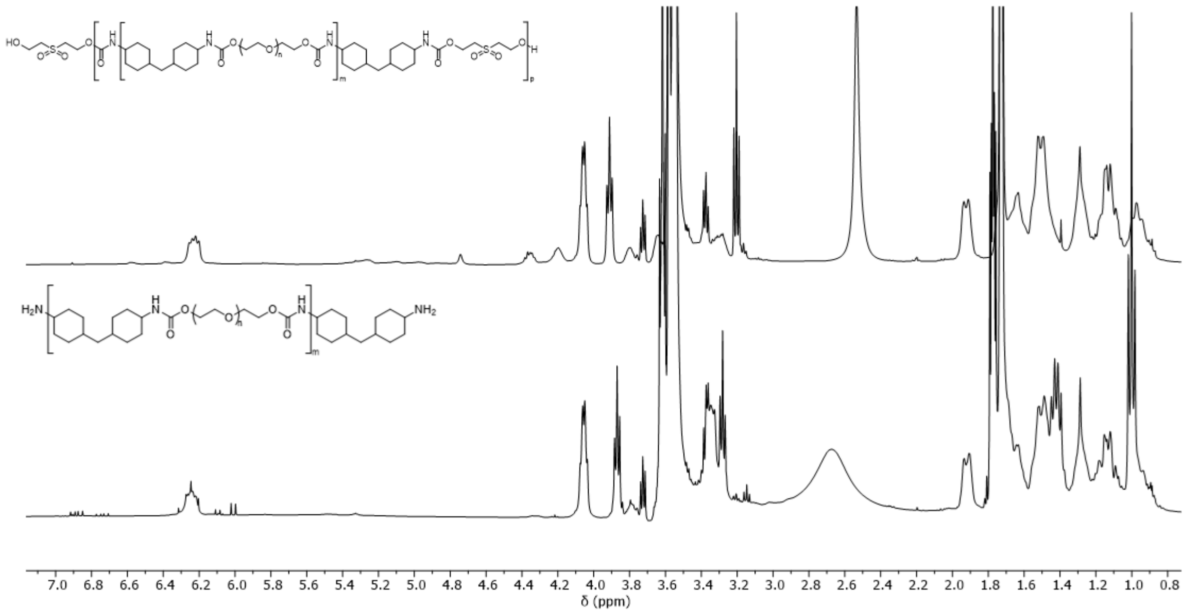

Figure S 57 ^{1}H NMR spectra showing the solution degradation of **CEPU2** with 1 M TBAF in THF, (400 MHz, THF-d_8). Top spectrum shows the pristine CEPU, bottom spectrum shows the degraded CEPU.

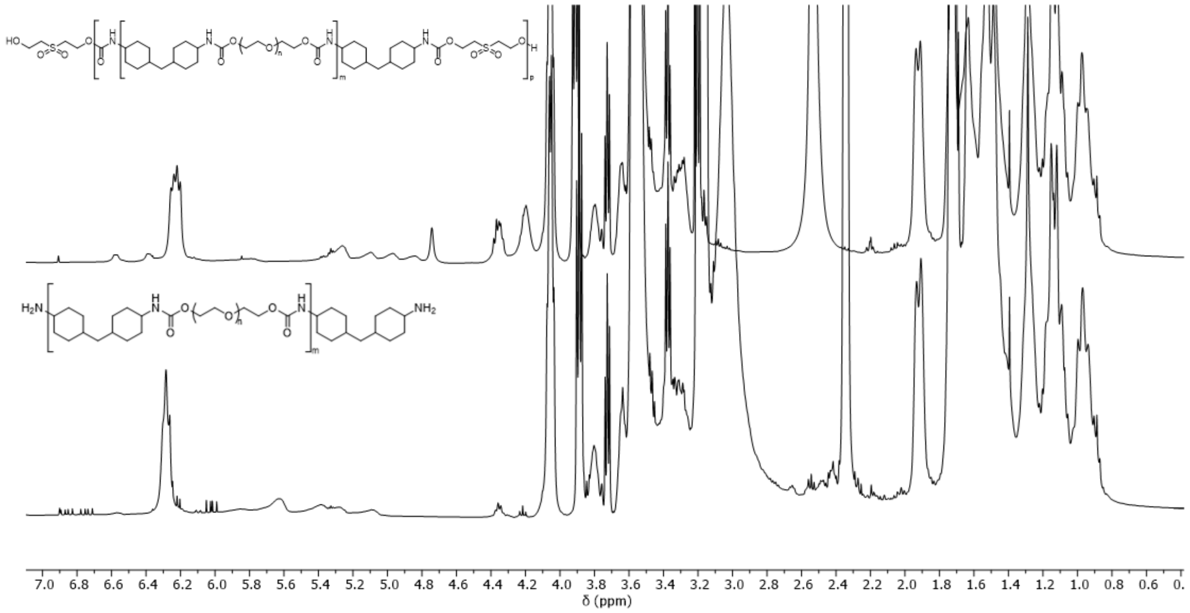

Figure S 58 ^{1}H NMR spectra showing the solution degradation of **CEPU2** with DBU, (400 MHz, THF-d_8). Top spectrum shows the pristine CEPU, bottom spectrum shows the degraded CEPU.

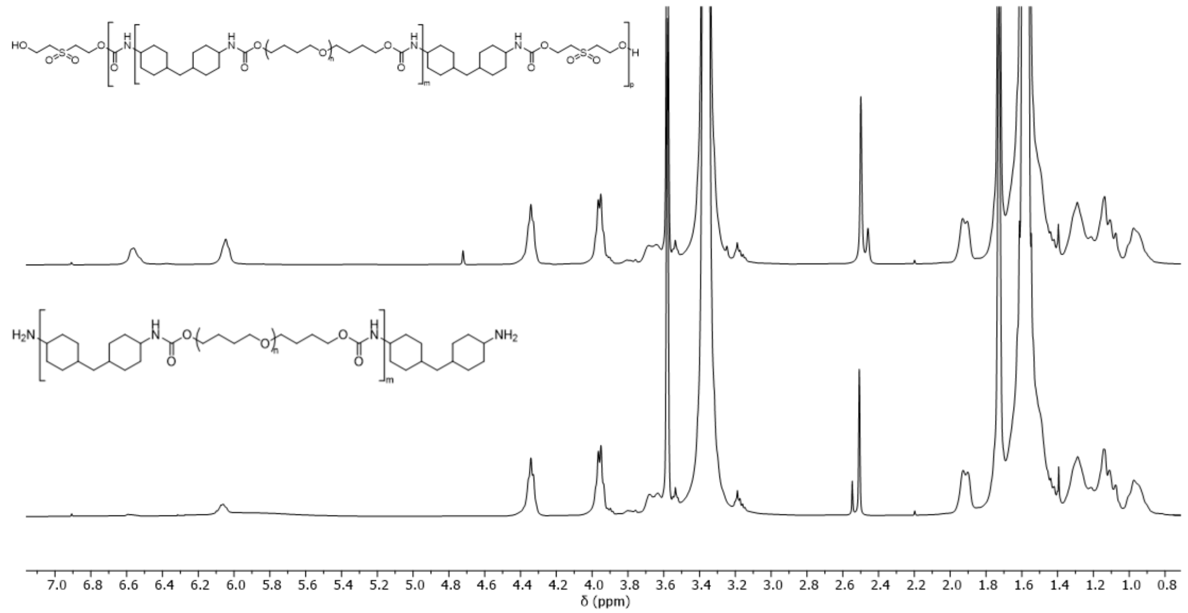

Figure S 59 ^{1}H NMR spectra showing the solution degradation of **CEPU3** with 40 wt.% NaOD in D_2O, (400 MHz, THF-d_8). Top spectrum shows the pristine CEPU, bottom spectrum shows the degraded CEPU.

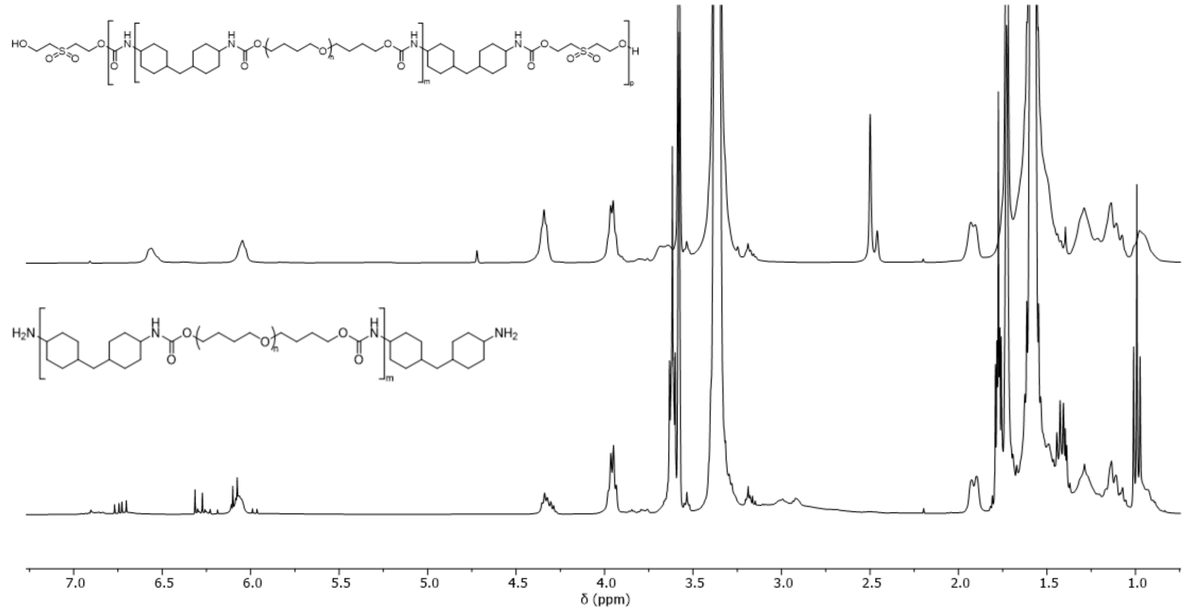

Figure S 60 ^{1}H NMR spectra showing the solution degradation of **CEPU3** with 1 M TBAF in THF, (400 MHz, THF-d_8). Top spectrum shows the pristine CEPU, bottom spectrum shows the degraded CEPU.

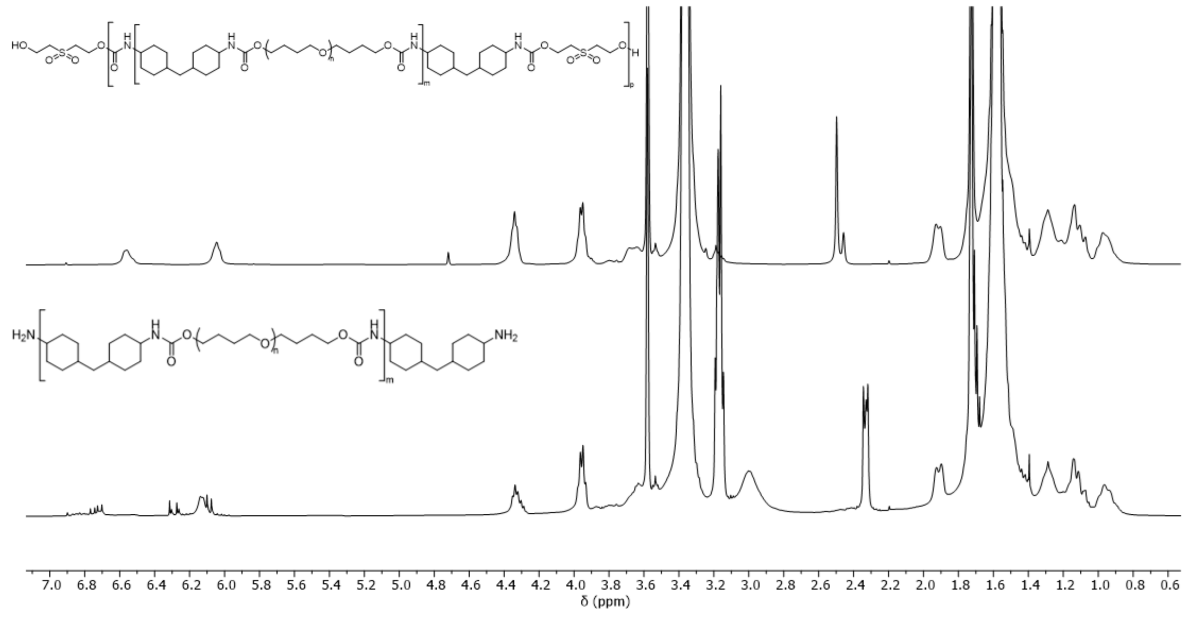

Figure S 61 ^{1}H NMR spectra showing the solution degradation of **CEPU3** with DBU, (400 MHz, THF-d_8). Top spectrum shows the pristine CEPU, bottom spectrum shows the degraded CEPU.

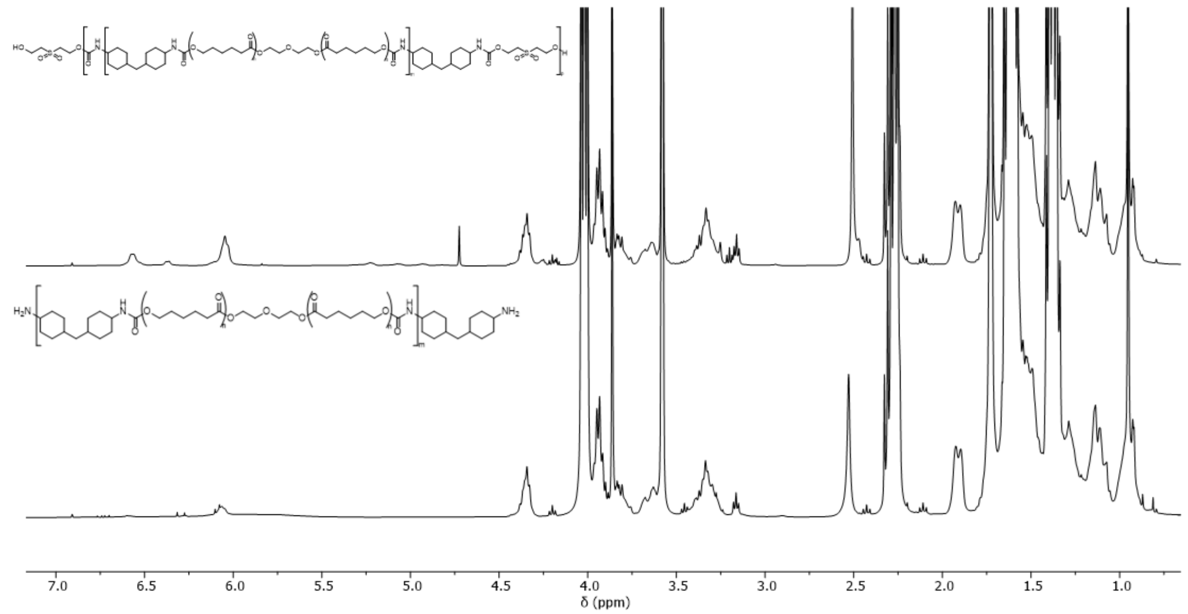

Figure S 62 ^{1}H NMR spectra showing the solution degradation of **CEPU4** with 40 wt.% NaOD in D_2O, (400 MHz, THF-d_8). Top spectrum shows the pristine CEPU, bottom spectrum shows the degraded CEPU.

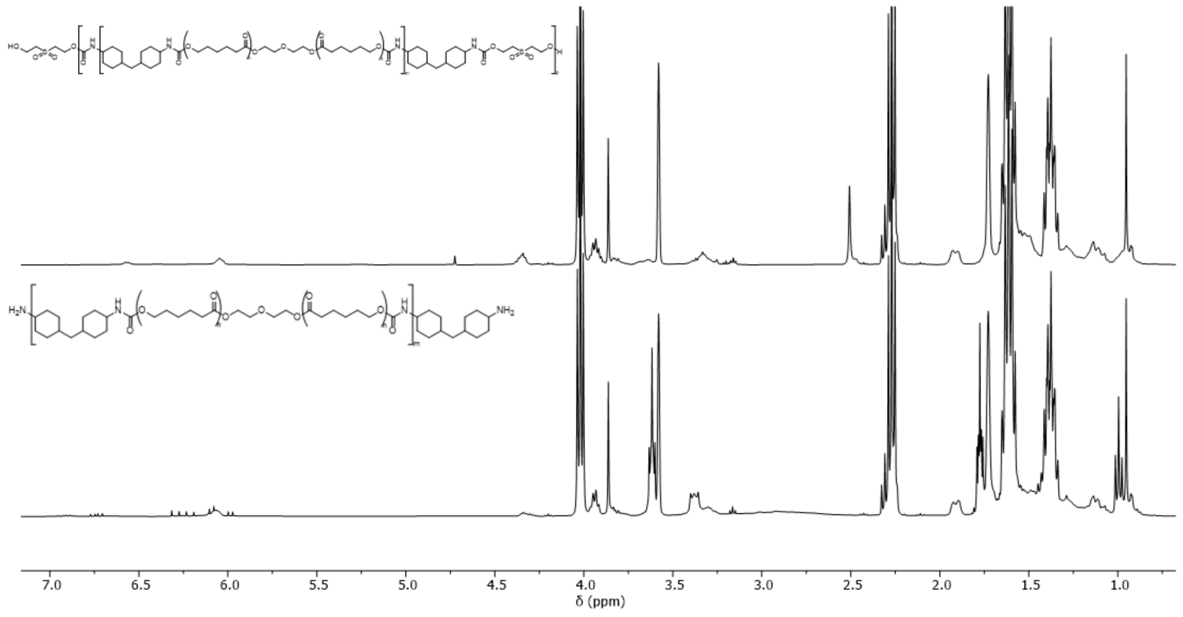

Figure S 63 ^{1}H NMR spectra showing the solution degradation of **CEPU4** with 1 M TBAF in THF, (400 MHz, THF-d_8). Top spectrum shows the pristine CEPU, bottom spectrum shows the degraded CEPU.

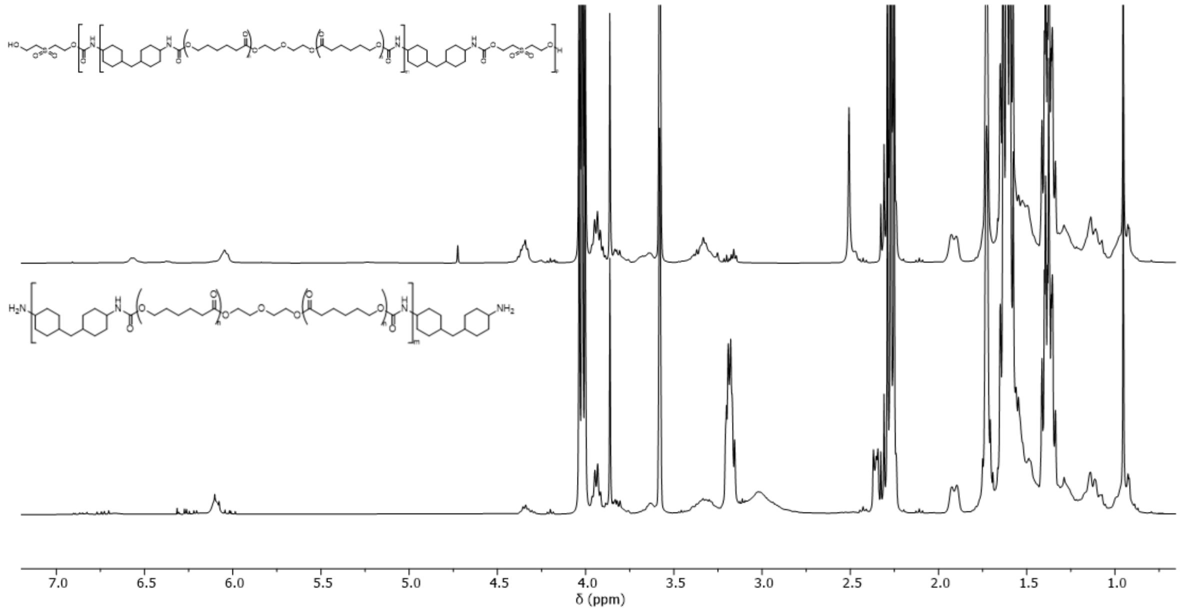

Figure S 64 ^{1}H NMR spectra showing the solution degradation of **CEPU4** with DBU, (400 MHz, THF-d_8). Top spectrum shows the pristine CEPU, bottom spectrum shows the degraded CEPU.

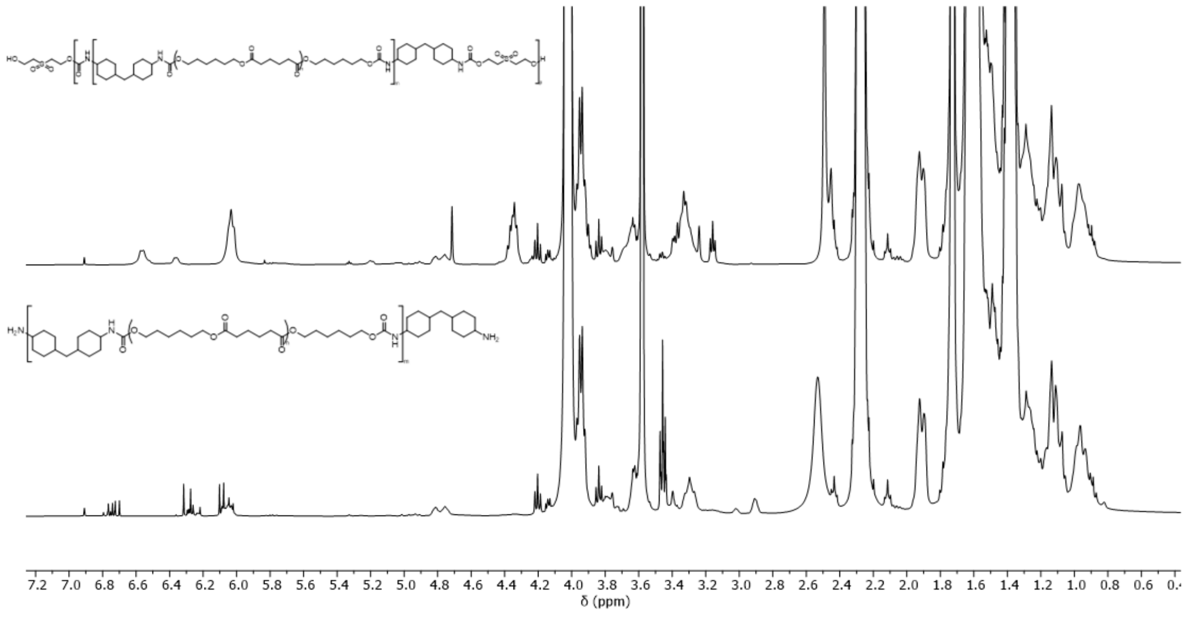

Figure S 65 ^{1}H NMR spectra showing the solution degradation of **CEPU5** with 40 wt.% NaOD in D_2O, (400 MHz, THF-d_8). Top spectrum shows the pristine CEPU, bottom spectrum shows the degraded CEPU.

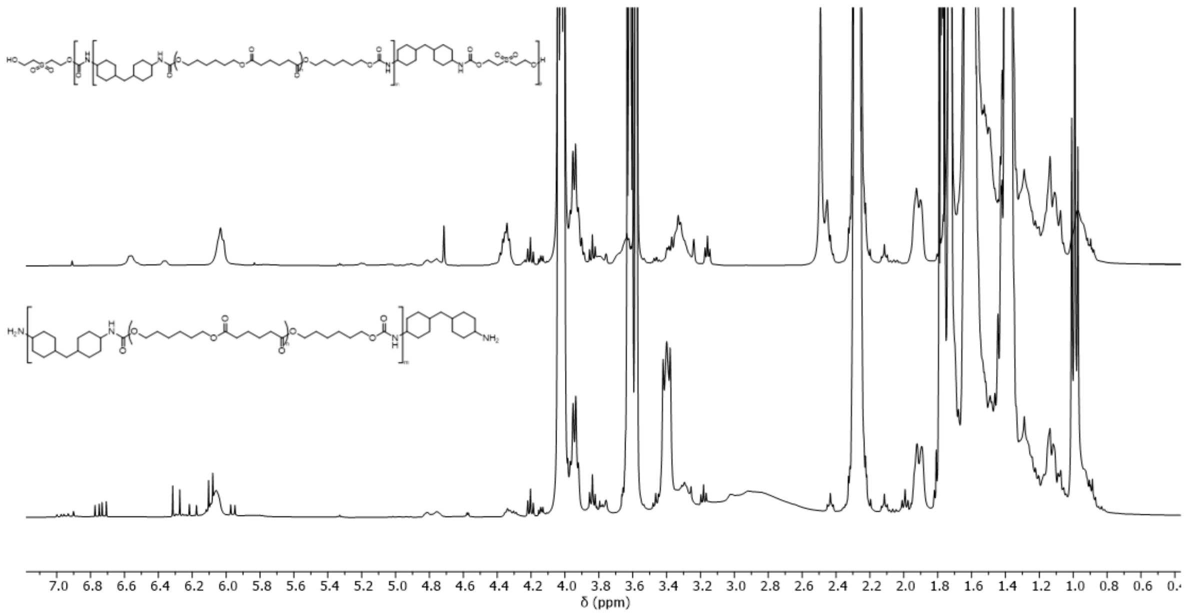

Figure S 66 ^{1}H NMR spectra showing the solution degradation of **CEPU5** with 1 M TBAF in THF, (400 MHz, THF-d_8). Top spectrum shows the pristine CEPU, bottom spectrum shows the degraded CEPU.

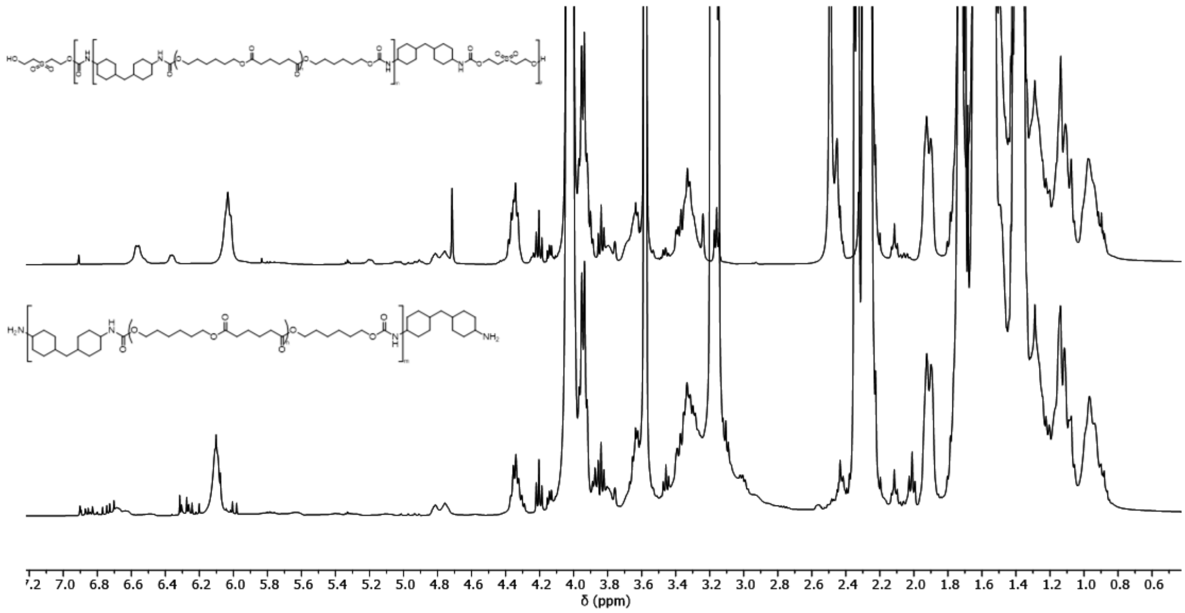

Figure S 67 ^{1}H NMR spectra showing the solution degradation of **CEPU5** with DBU, (400 MHz, THF-d_8). Top spectrum shows the pristine CEPU, bottom spectrum shows the degraded CEPU.

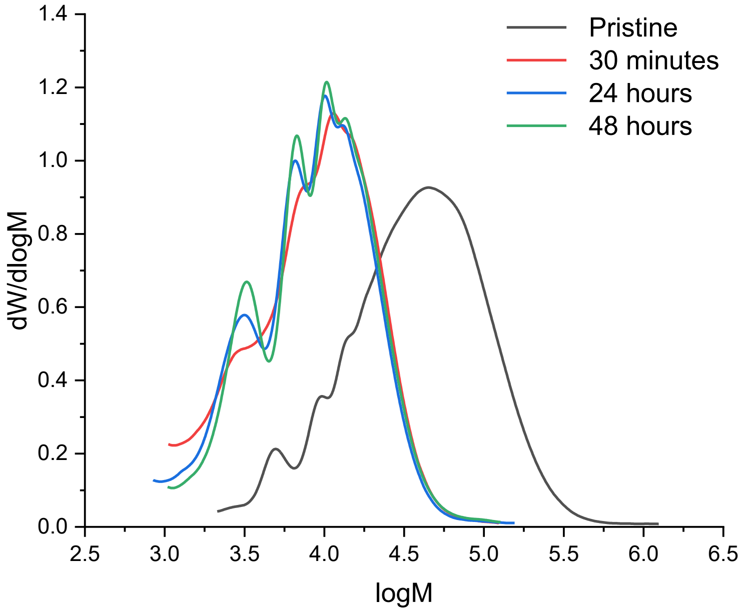

Figure S 68 GPC eluogram of **CEPU2** in THF as a pristine sample and 30 min, 24 hr, and 48 hr post addition of TBAF.

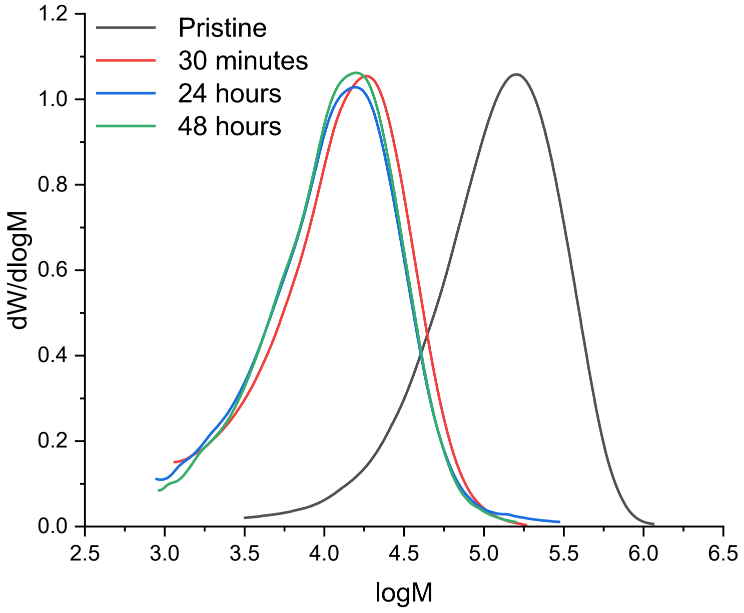

Figure S 69 GPC eluogram of **CEPU3** in THF as a pristine sample and 30 min, 24 hr, and 48 hr post addition of TBAF.

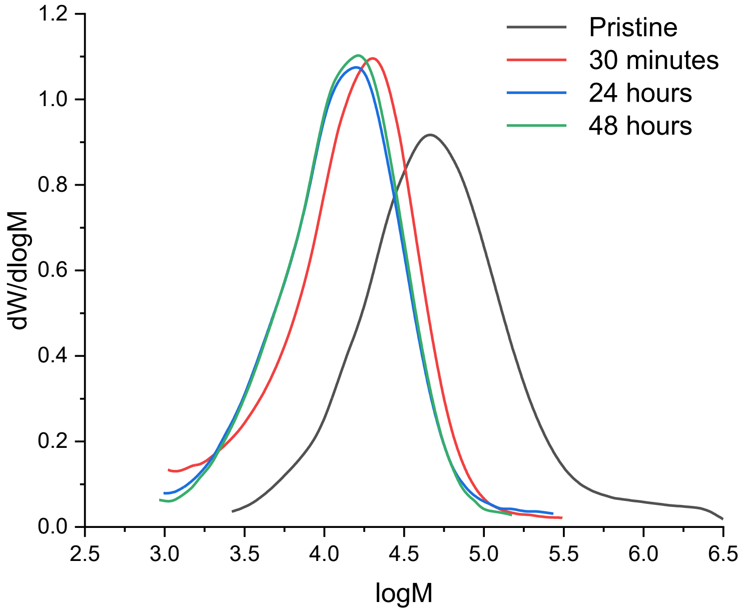

Figure S 70 GPC eluogram of **CEPU4** in THF as a pristine sample and 30 min, 24 hr, and 48 hr post addition of TBAF.

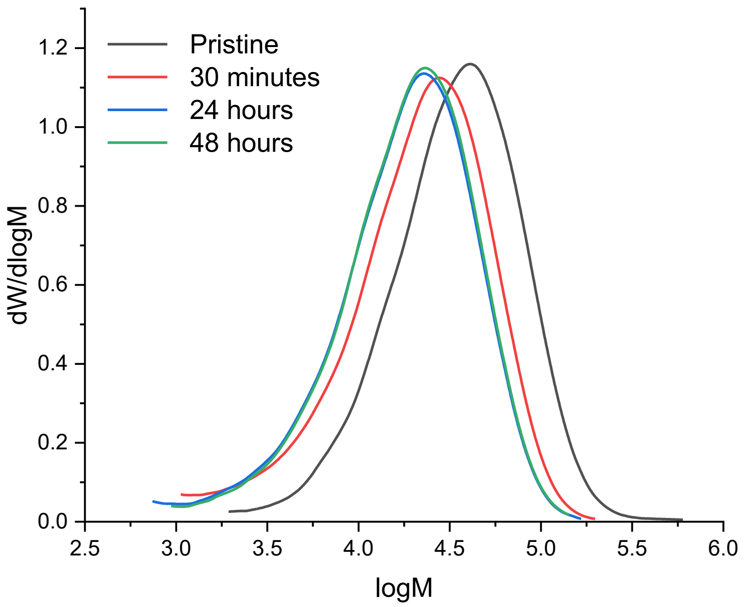

Figure S 71 GPC eluogram of **CEPU5** in THF as a pristine sample and 30 min, 24 hr, and 48 hr post addition of TBAF.

Table S 2 M_n and M_w of **CEPU1-CEPU5** as pristine samples and 30 min, 24 hr, and 48 hr post addition of TBAF acquired from a THF GPC; the recorded are averages of three separate samples of each CEPU. The error shown is the standard deviation between the three repeats of each sample.

CEPU	Exposure time	M_n (g mol^{-1})	M_w (g mol^{-1})	Đ
**CEPU1**	Pristine	44700 ± 200	140400 ± 700	3.14
	30 minutes	7800 ± 200	15600 ± 500	2.00
	24 hours	7700 ± 0	14000 ± 0	1.82
	48 hours	8100 ± 0	15000 ± 200	1.85
**CEPU2**	Pristine	22200 ± 100	62600 ± 1300	2.82
	30 minutes	5600 ± 0	12500 ± 200	2.23
	24 hours	5600 ± 0	12400 ± 300	2.21
	48 hours	6200 ± 0	12300 ± 100	1.98
**CEPU3**	Pristine	64800 ± 900	167800 ± 300	2.59
	30 minutes	7900 ± 100	19600 ± 400	2.48
	24 hours	7100 ± 100	17100 ± 200	2.41
	48 hours	7400 ± 100	17000 ± 100	2.30
**CEPU4**	Pristine	28100 ± 200	124300 ± 1600	4.42
	30 minutes	8800 ± 100	21700 ± 500	2.47
	24 hours	7700 ± 100	17500 ± 900	2.27
	48 hours	8200 ± 100	16900 ± 300	2.06
**CEPU5**	Pristine	23600 ± 100	45700 ± 300	1.94
	30 minutes	12900 ± 200	29400 ± 100	2.28
	24 hours	11400 ± 300	24800 ± 100	2.18
	48 hours	11800 ± 0	25000 ± 0	2.12

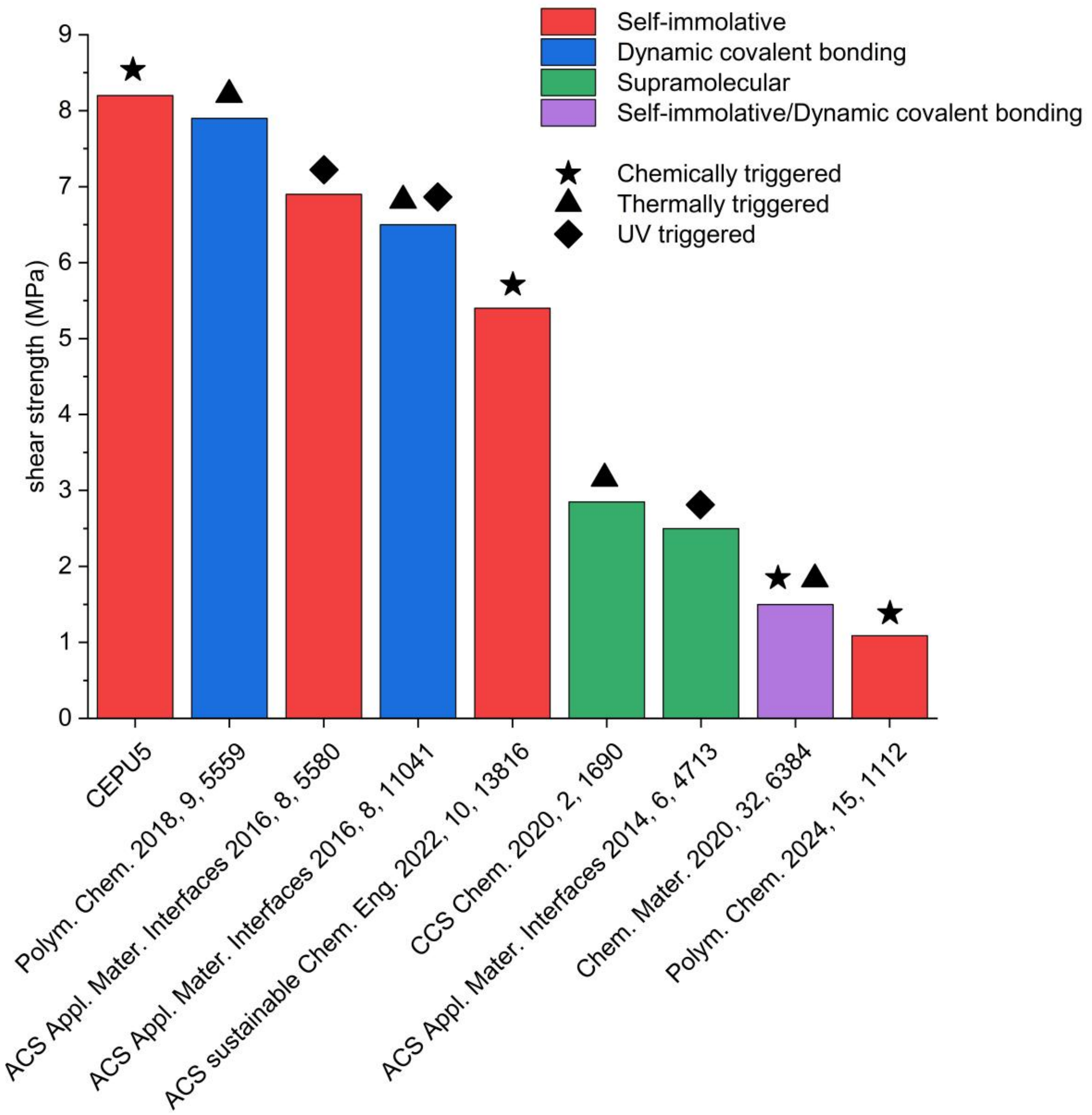

Figure S 72 Glass adhered shear strength of **CEPU5** compared to other debond-on-demand adhesives. [2–9]

Table S 3 Shear strength of the best CEPU adhesive on aluminium, glass, high density poly(ethylene) (HDPE), poly(propylene) (PP), Nylon, polyethylene terephthalate (PET), and polyvinyl chloride (PVC) as the pristine sample and after exposure to deionised water, 40 wt.% NaOH(aq), 1 M TBAF(aq), and 1 M DBU(aq). Percentages shown are the variation between the pristine and treated samples. The error shown is the standard deviation between the three repeats of each sample.

Substrate (CEPU adhesive)	Pristine (MPa)	Deionised water (MPa)	40 wt.% $NaOH_{(aq)}$ (MPa)	1 M $TBAF_{(aq)}$ (MPa)	1 M $DBU_{(aq)}$ (MPa)
Aluminium (**CEPU1**)	2.69 ± 0.09	2.51 ± 0.13<br>-7%	0.78 ± 0.59<br>-71%	0.90 ± 0.04<br>-67%	1.08 ± 0.32<br>-60%
Glass (**CEPU5**)	8.20 ± 0.13	7.76 ± 0.29<br>-5%	0.62 ± 0.37<br>-92%	0.89 ± 0.25<br>-89%	2.09 ± 0.58<br>-74%
HDPE (**CEPU3**)	0.95 ± 0.06	1.01 ± 0.17<br>+6%	0.57 ± 0.06<br>-40%	0.43 ± 0.05<br>-54%	0.48 ± 0.02<br>-49%
PP (**CEPU4**)	1.55 ± 0.11	1.49 ± 0.25<br>-4%	0.40 ± 0.10<br>-74%	0.35 ± 0.06<br>-77%	0.36 ± 0.03<br>-77%
Nylon (**CEPU1**)	1.96 ± 0.15	1.87 ± 0.07<br>-5%	0.99 ± 0.03<br>-49%	1.10 ± 0.11<br>-44%	1.27 ± 0.10<br>-35%
PET (**CEPU5**)	1.18 ± 0.03	1.13 ± 0.28<br>-4%	0.80 ± 0.04<br>-33%	0.53 ± 0.38<br>-55%	0.70 ± 0.04<br>-41%
PVC (**CEPU5**)	7.35 ± 0.31	7.19 ± 0.40<br>-2%	2.36 ± 0.33<br>-68%	2.10 ± 0.41<br>-71%	1.75 ± 0.28<br>-76%

Table S 4 Solubility of **CEPU1-CEPU5** in a range of organic solvents after 24 hours.

Solvent	**CEPU1**	**CEPU2**	**CEPU3**	**CEPU4**	**CEPU5**
water	0	1	0	0	0
acetone	0	1	0	1	1
butanone	0	1	1	1	1
methanol	0	1	0	0	0
ethanol	0	1	0	0	0
isopropanol	0	0	0	0	0
THF	1	1	1	1	1
2-methyltetrahydrofuran	1	1	1	1	1
dimethylformamide	0	1	1	1	1
dimethylacetamide	0	1	1	1	1
acetonitrile	0	1	0	0	0
chloroform	1	1	1	1	1
dichloromethane	1	1	1	1	1
hexane	0	0	0	0	0
toluene	1	1	1	1	1
m-xylene	1	1	1	0	1
ethyl acetate	0	1	1	1	1
diethyl ether	1	0	0	0	0
petroleum ether	0	0	0	0	0
cyclohexane	0	0	0	0	0
1,4-dioxane	0	1	1	1	1
benzyl alcohol	0	1	1	1	1
benzonitrile	0	1	1	1	1
benzaldehyde	1	1	1	1	1
2-(2-methoxyethoxy)ethanol	0	1	0	0	0
2-methoxyethanol	0	1	1	1	0
2-isopropyl ethanol	0	1	1	0	0
propylene carbonate	0	1	0	0	1
tert-butyl methyl ether	1	0	0	0	0
diethylene glycol dimethylether	0	1	1	1	0
**total**	**9**	**24**	**17**	**16**	**16**

1 = fully soluble; 0 = insoluble; water and petroleum ether were excluded form HSP solubility sphere calculations.

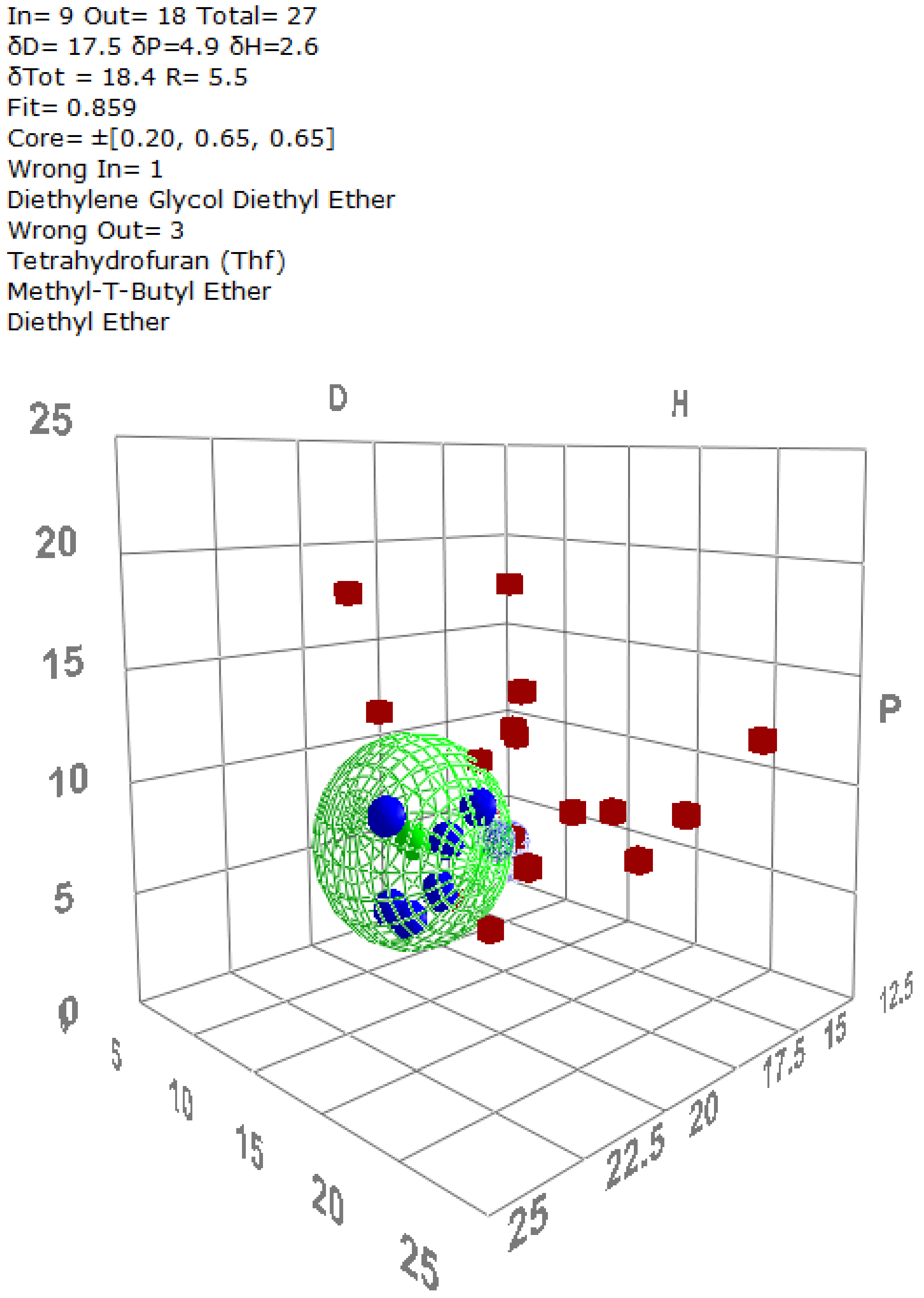

Figure S 73 HSP solubility sphere of **CEPU1**.

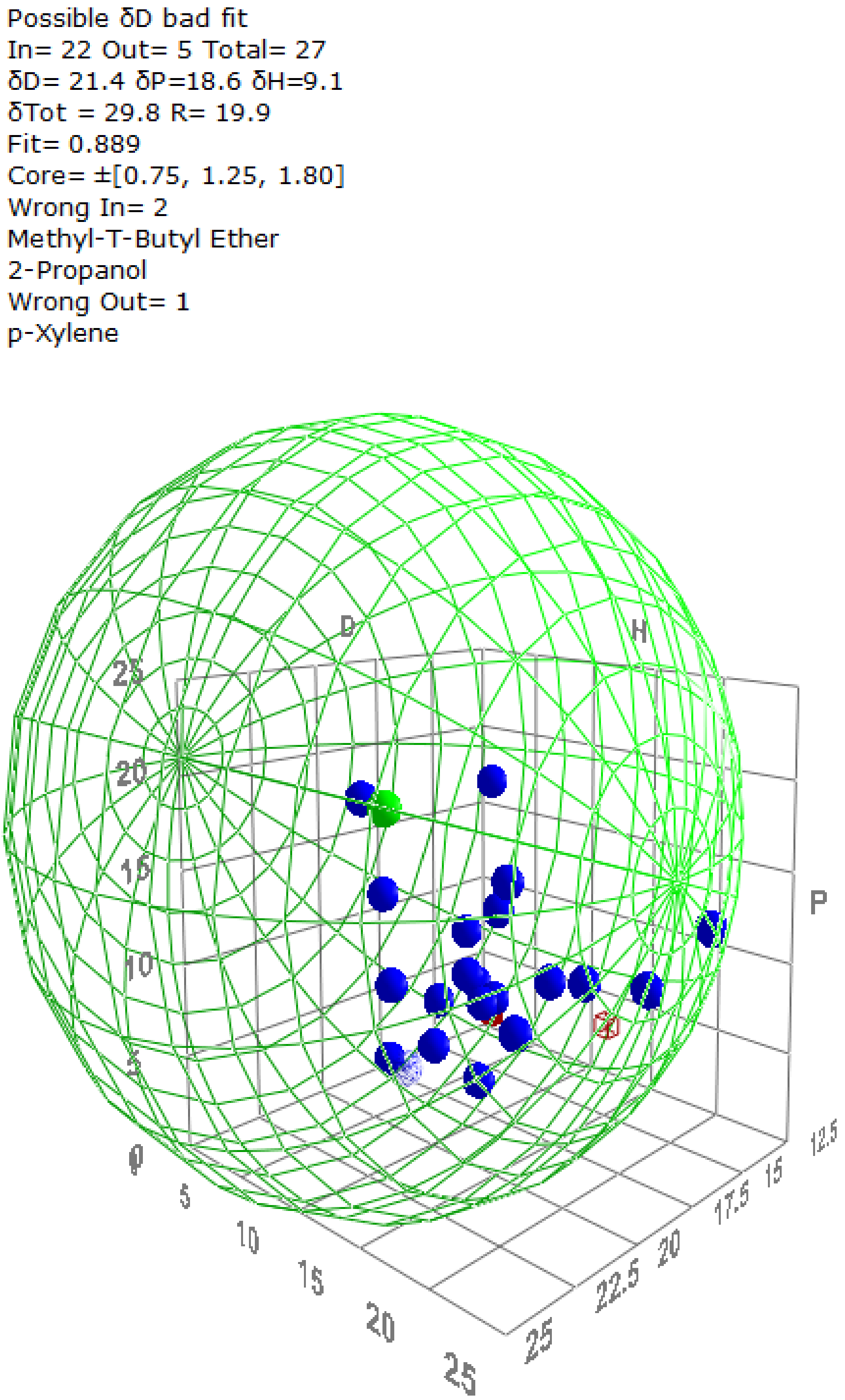

Figure S 74 HSP solubility sphere of **CEPU2**.

In= 17 Out= 10 Total= 27
δD= 19.1 δP=5.9 δH=9.2
δTot = 22.0 R= 8.8
Fit= 1.000
Core= ±[0.15, 0.35, 0.30]
Wrong In= 0
Wrong Out= 0

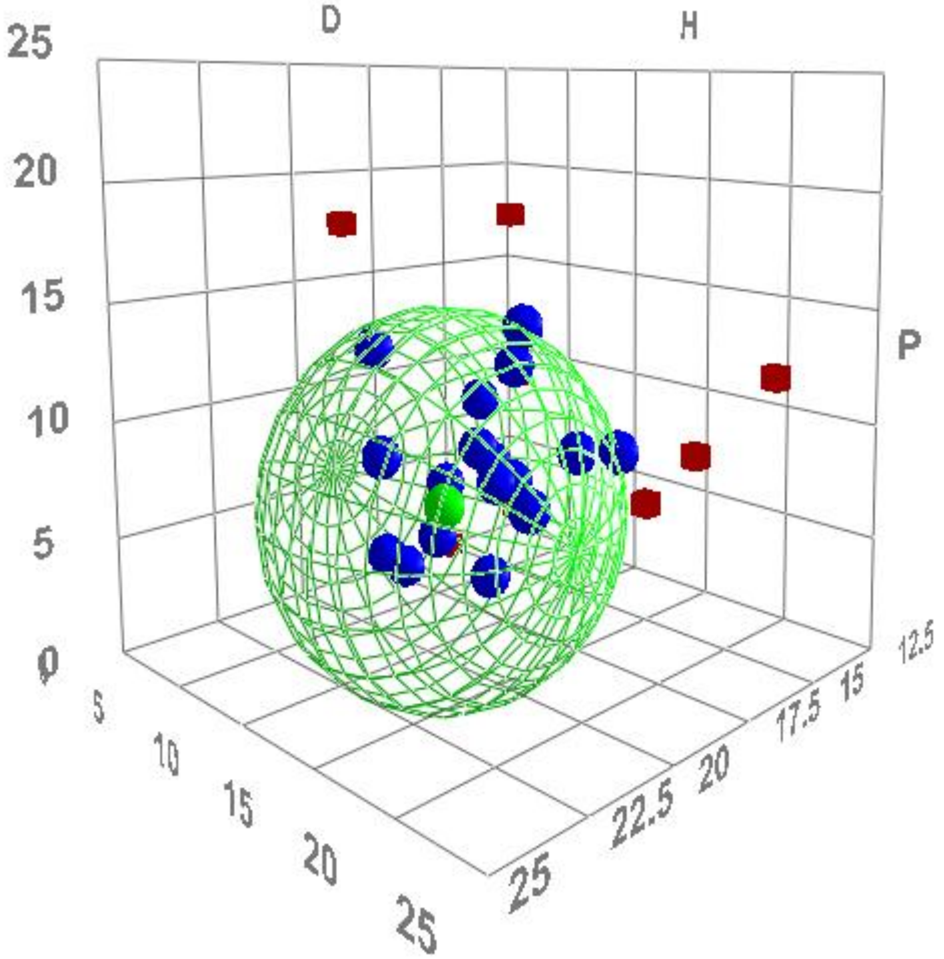

Figure S 75 HSP solubility sphere of **CEPU3**.

Possible δD bad fit
In= 16 Out= 11 Total= 27
δD= 21.0 δP=8.2 δH=9.4
δTot = 24.4 R= 11.3
Fit= 0.890
Core= ±[0.15, 0.35, 0.45]
Wrong In= 1
Diethylene Glycol Monomethyl Ether
Wrong Out= 3
Acetone
Ethylene Glycol Monomethyl Ether
Toluene

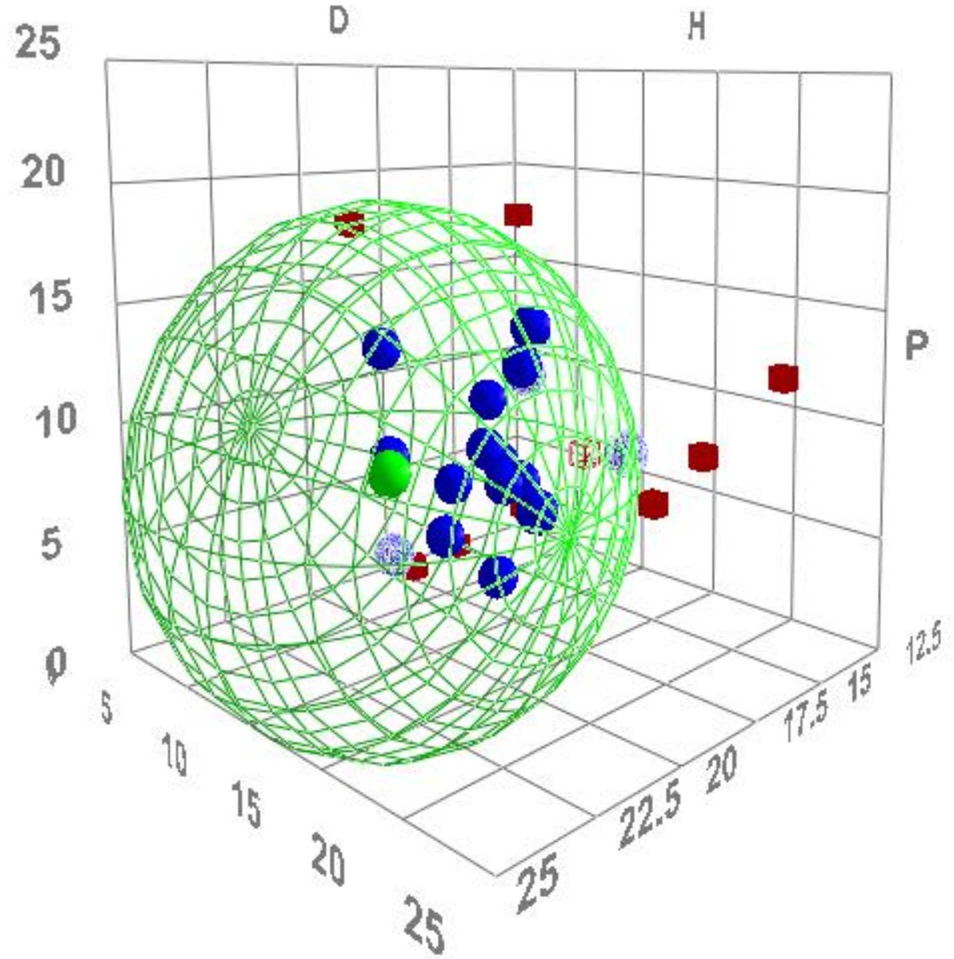

Figure S 76 HSP solubility sphere of **CEPU4**.

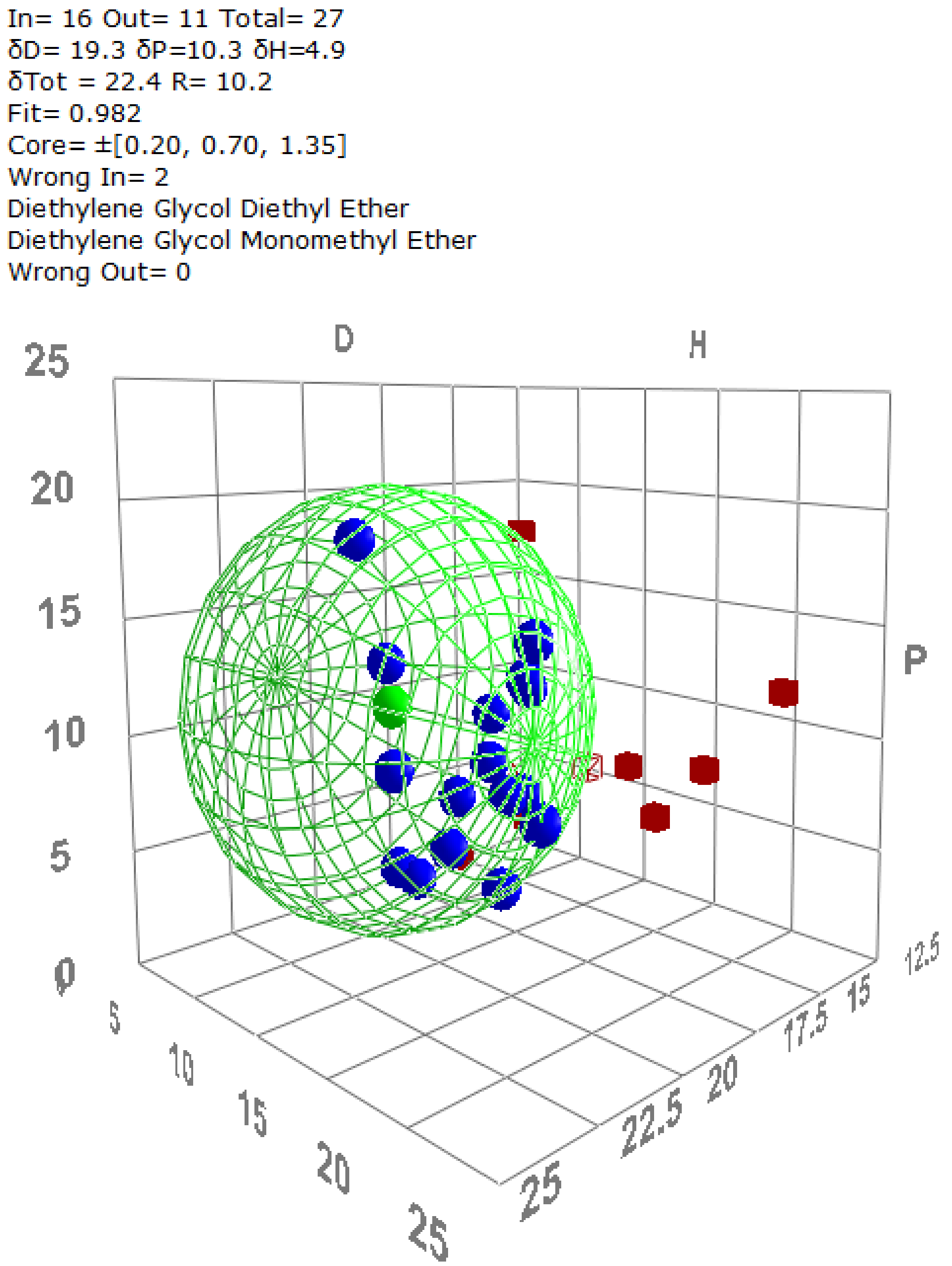

Figure S 77 HSP solubility sphere of **CEPU5**.

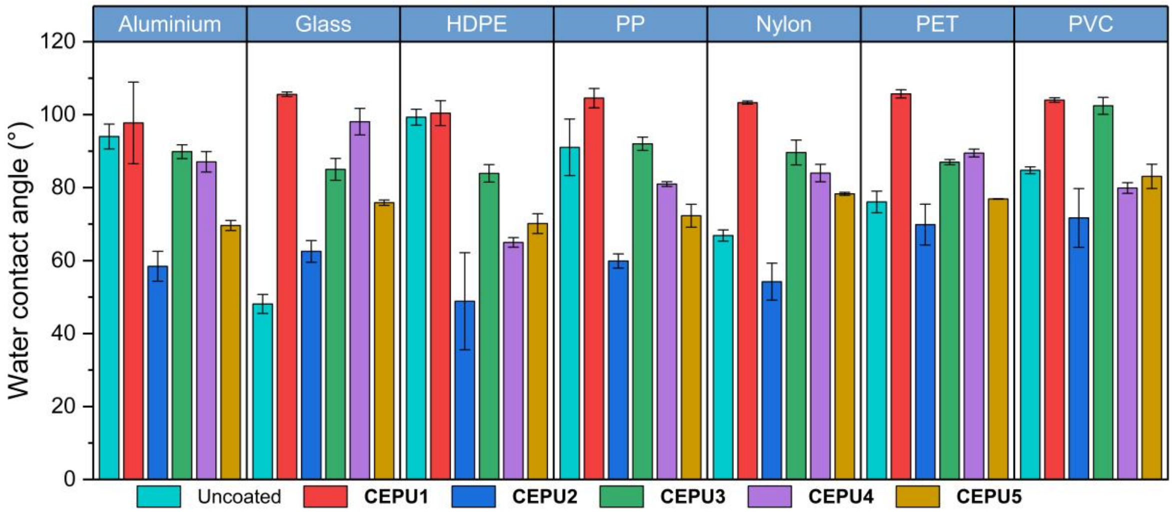

Figure S 78 Water contact angles on CEPU coated surfaces. The error shown is the standard deviation between the three repeats of each sample.

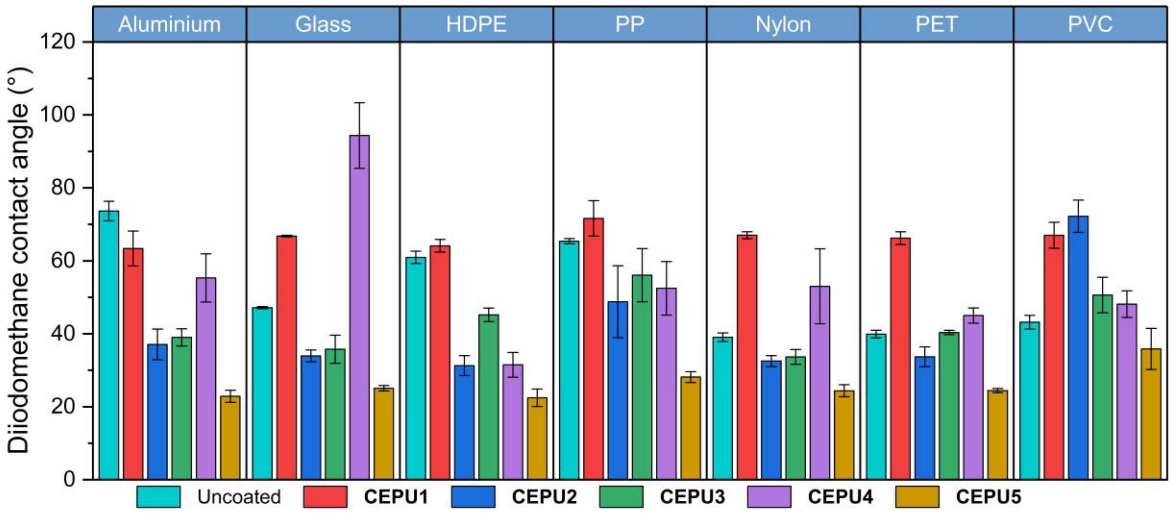

Figure S 79 Diiodomethane contact angles on CEPU coated surfaces. The error shown is the standard deviation between the three repeats of each sample.

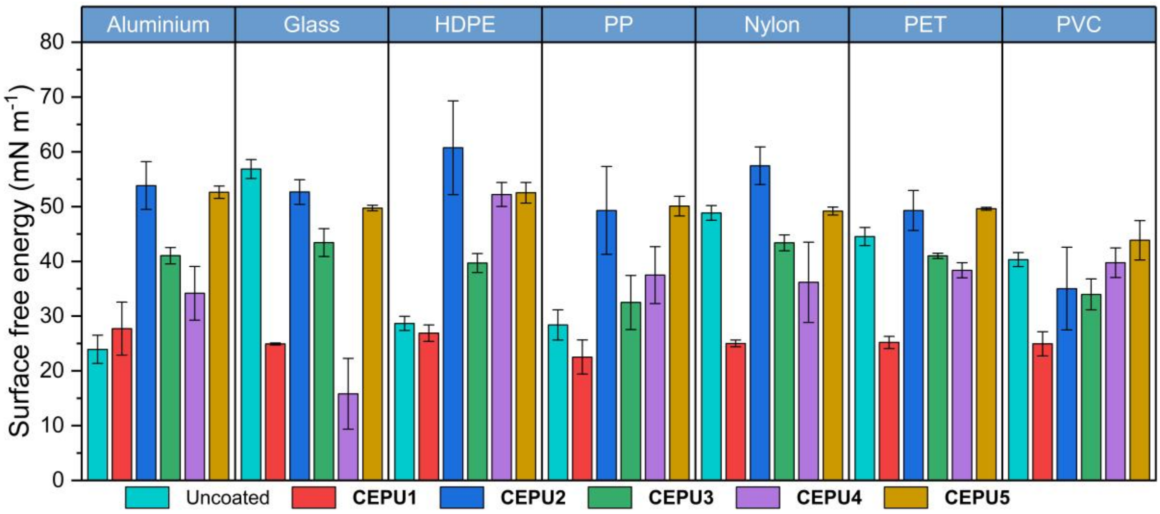

Figure S 80 Surface free energy of CEPU coated surfaces. The error shown is the standard deviation between the three repeats of each sample.

Table S 5 Water contact angles, diiodomethane contact angles, and surface free energies of uncoated and CEPU coated surfaces. The error shown is the standard deviation between the three repeats of each sample.

CEPU	Surface	Water contact angle (°)	Diiodomethane contact angle (°)	Surface free energy (mN m^{-1})
Uncoated	Aluminium	93.99 ± 3.41	73.66 ± 2.67	23.94 ± 2.58
	Glass	48.11 ± 2.61	47.17 ± 0.27	56.87 ± 1.73
	HDPE	99.32 ± 2.18	60.97 ± 1.67	28.67 ± 1.29
	PP	91.05 ± 7.76	65.37 ± 0.72	28.39 ± 1.29
	Nylon	66.87 ± 1.54	39.06 ± 1.16	48.84 ± 2.76
	PET	76.07 ± 2.95	39.91 ± 1.04	44.53 ± 1.34
	PVC	84.77 ± 0.93	43.18 ± 1.88	40.32 ± 1.65
**CEPU1**	Aluminium	97.73 ± 11.20	63.38 ± 4.78	27.71 ± 1.29
	Glass	105.58 ± 0.62	66.78 ± 0.22	24.92 ± 4.83
	HDPE	100.40 ± 3.41	64.13 ± 1.73	26.89 ± 0.18
	PP	104.54 ± 2.65	71.63 ± 4.84	22.53 ± 1.50
	Nylon	103.30 ±0.43	67.02 ± 0.95	25.02 ± 3.12
	PET	105.70 ± 1.13	66.24 ± 1.74	25.20 ± 0.61
	PVC	103.99 ± 0.62	67.01 ± 3.56	24.95 ± 1.10
**CEPU2**	Aluminium	58.44 ± 4.10	37.07 ± 4.20	53.84 ± 2.20
	Glass	62.52 ± 2.98	33.95 ± 1.63	52.66 ± 4.36
	HDPE	48.85 ± 13.30	31.28 ± 2.75	60.74 ± 2.26
	PP	59.89 ± 1.95	48.79 ± 9.86	49.30 ± 8.55
	Nylon	54.22 ± 5.07	32.52 ± 1.50	57.45 ± 8.01
	PET	69.87 ± 5.60	33.70 ± 2.70	49.29 ± 3.44
	PVC	71.68 ± 8.04	72.22 ± 4.42	35.02 ± 3.65
**CEPU3**	Aluminium	89.86 ±1.88	39.02 ± 2.34	41.04 ± 7.56
	Glass	85 ± 3.00	35.77 ± 3.84	43.42 ± 1.50
	HDPE	83.91 ± 2.40	45.19 ± 1.85	39.69 ± 2.54
	PP	92.02 ± 1.81	56.08 ± 7.28	32.49 ± 1.72
	Nylon	89.63 ± 3.40	33.66 ± 2.03	43.38 ± 1.44
	PET	86.99 ± 0.74	40.37 ± 0.60	41.01 ± 0.47
	PVC	102.43 ± 2.33	50.61 ± 4.86	33.97 ± 2.82
**CEPU4**	Aluminium	87.07 ± 2.79	55.30 ± 6.61	34.17 ± 24.91
	Glass	98.08 ± 3.62	94.35 ± 0.75	15.81 ± 11.46
	HDPE	64.98 ± 1.32	31.50 ± 2.41	52.21 ± 2.20
	PP	80.98 ± 0.64	52.48 ± 1.47	37.49 ± 5.20
	Nylon	84.00 ± 2.39	53.02 ± 1.64	36.18 ± 7.33
	PET	89.48 ± 1.08	45.00 ± 0.58	38.37 ± 1.38
	PVC	79.90 ± 1.46	48.14 ± 5.62	39.74 ± 2.71
**CEPU5**	Aluminium	69.64 ± 1.39	22.86 ± 1.67	52.62 ± 1.12
	Glass	75.89 ± 0.73	25.08 ± 0.75	49.73 ± 0.51
	HDPE	70.14 ± 2.71	22.47 ± 2.41	52.52 ± 1.87
	PP	72.30 ± 3.14	28.13 ± 1.47	50.09 ± 1.79
	Nylon	78.31 ± 0.41	24.38 ± 1.64	49.18 ± 0.72
	PET	76.89 ± 0.10	24.43 ± 0.58	49.60 ± 0.25
	PVC	83.10 ± 3.32	35.85 ± 5.62	43.85 ± 3.60

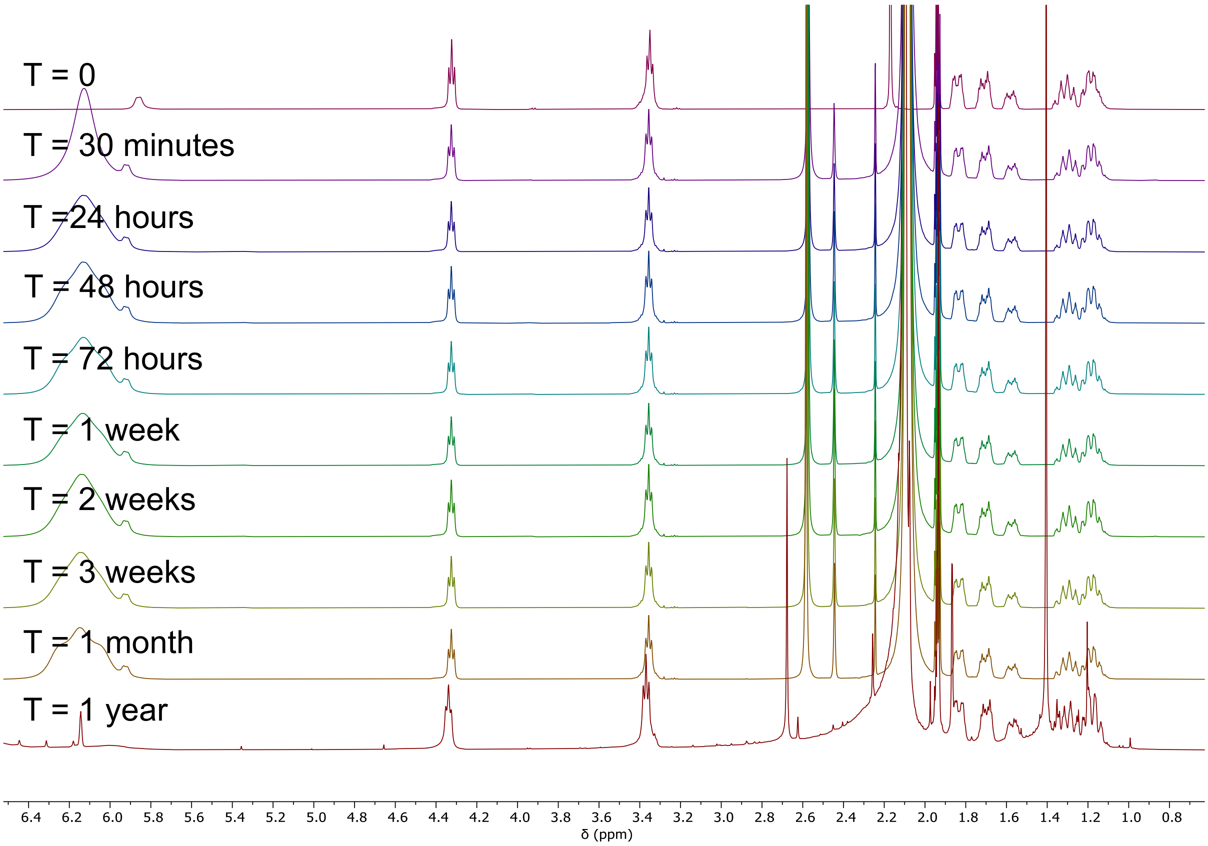

Figure S 81 ^{1}H NMR stability of model small molecule **1** with $TBAPF_6$ over 1 year.

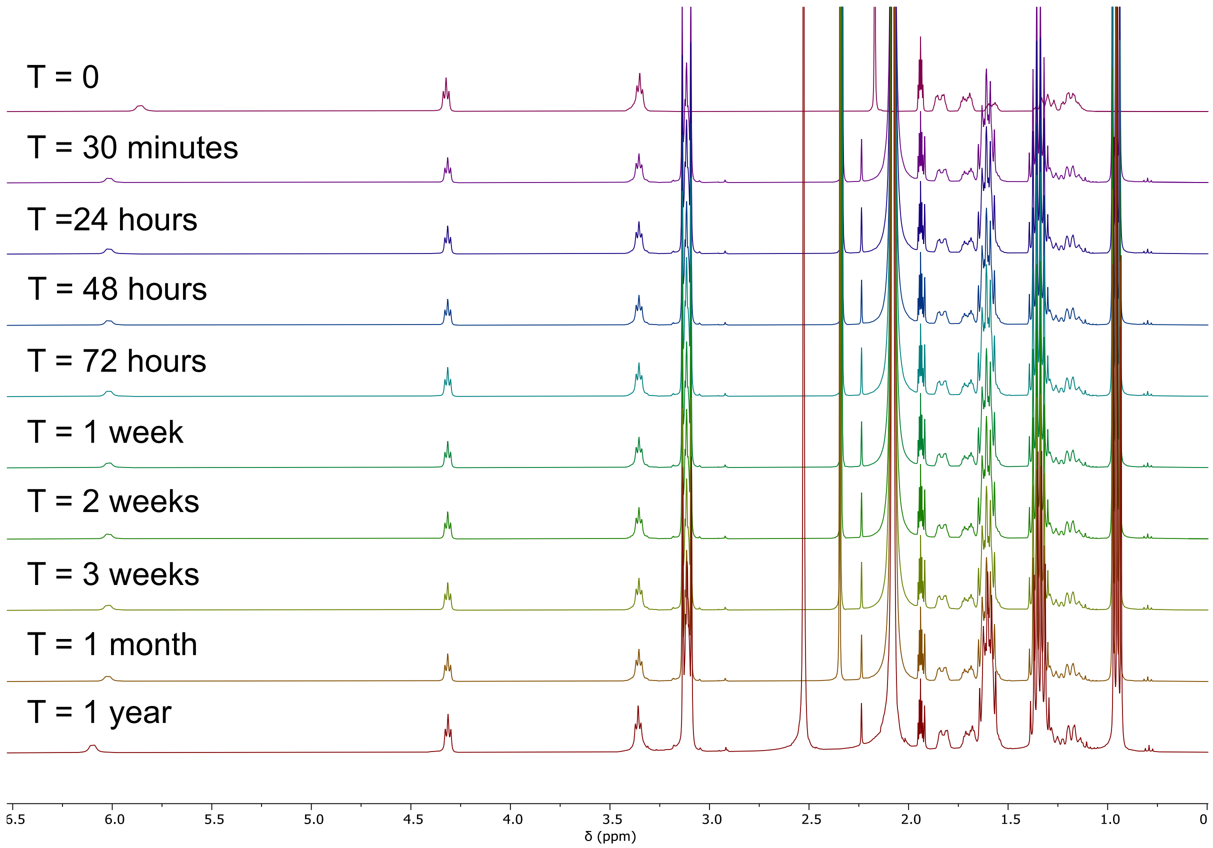

Figure S 82 ^{1}H NMR stability of model small molecule **1** with TBAN over 1 year.

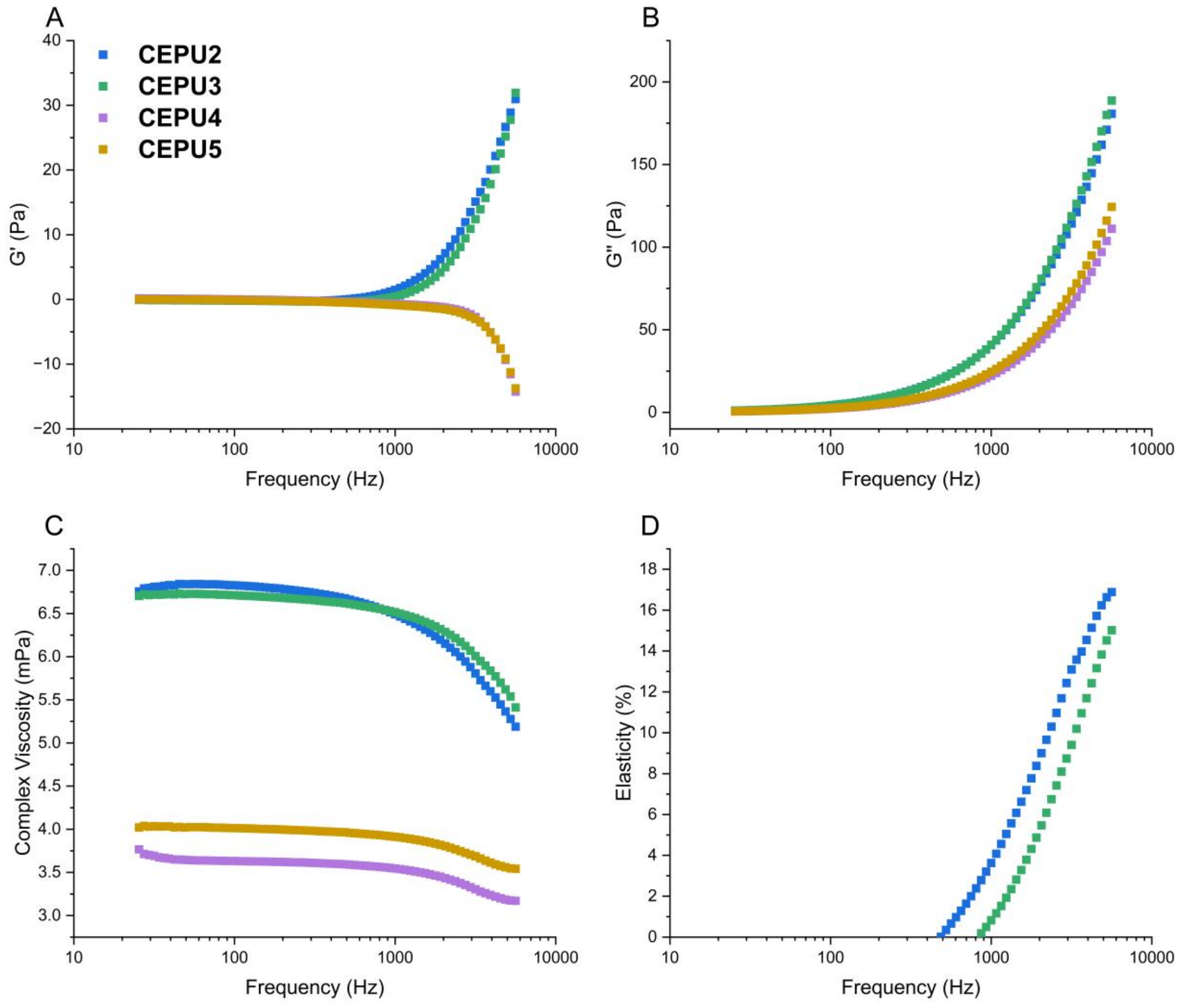

Figure S 83 PAV rheology of inkjet formulations of **CEPU2**-**CEPU5** from 10-10000 Hz at 25 °C. **A** Storage modulus (G'), **B** Loss modulus (G''), **C** Complex viscosity, and **D** Percentage viscosity.

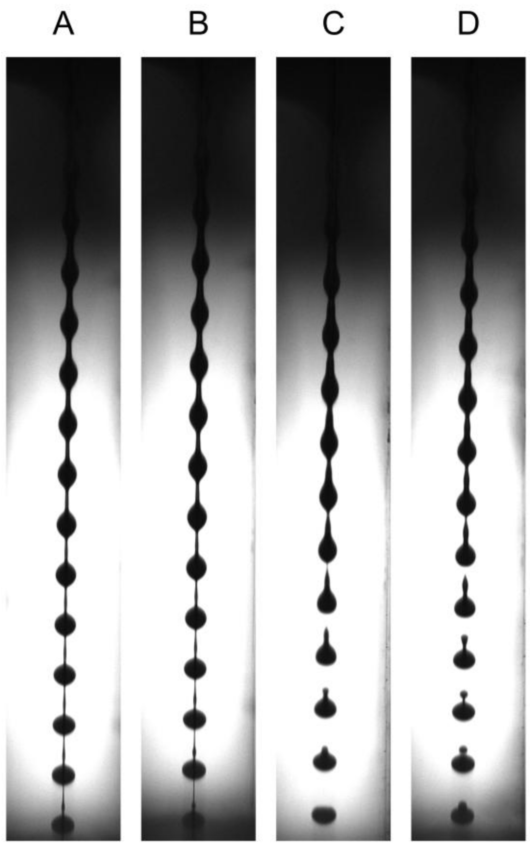

Figure S 84 CIJ jet stream break-up of inkjet formulations; **A CEPU2**, **B CEPU3**, **C CEPU4**, **D CEPU5**.

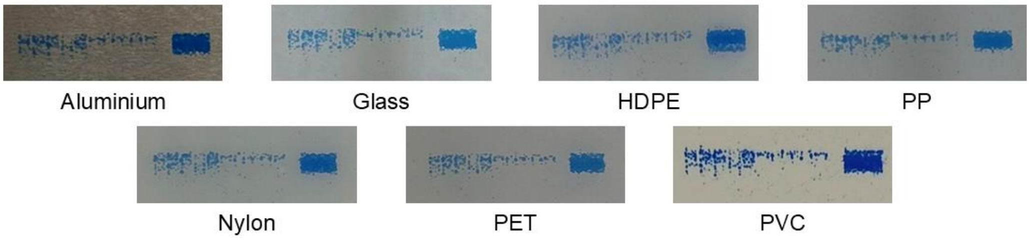

Figure S 85 CIJ deposition of **CEPU2** formulations onto aluminium, glass, high density poly(ethylene) (HDPE), poly(propylene) (PP), Nylon, polyethylene terephthalate (PET), and polyvinyl chloride (PVC).

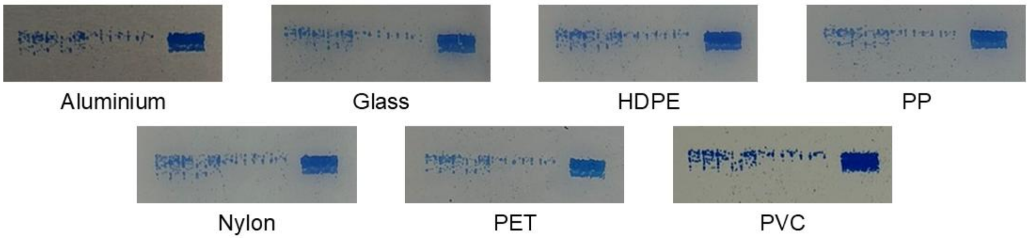

Figure S 86 CIJ deposition of **CEPU3** formulations onto aluminium, glass, high density poly(ethylene) (HDPE), poly(propylene) (PP), Nylon, polyethylene terephthalate (PET), and polyvinyl chloride (PVC).

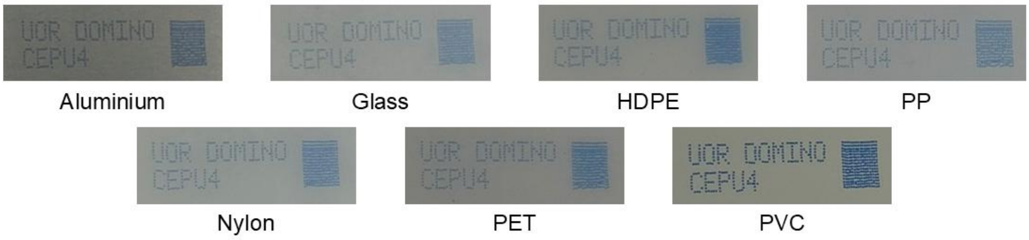

Figure S 87 CIJ deposition of **CEPU4** formulations onto aluminium, glass, high density poly(ethylene) (HDPE), poly(propylene) (PP), Nylon, polyethylene terephthalate (PET), and polyvinyl chloride (PVC).

Table S 6 Peel test adhesion evaluation of CEPU formulations using adhesive tape (810 grade). The amount of material removed from the surface was graded by an arbitrary value between 1 and 5 (where 5 indicated no removal of the print (excellent adhesion) and 1 indicated the complete removal of the print (very poor adhesion)).

CEPU formulation	Aluminium	Glass	HDPE	PP	Nylon	PET	PVC
**CEPU1**	1	5	1	3	5	5	5
**CEPU2**	2	5	1	1	5	5	5
**CEPU3**	1	5	1	1	5	5	5
**CEPU4**	2	5	1	1	5	5	5
**CEPU5**	3	5	1	2	5	5	5

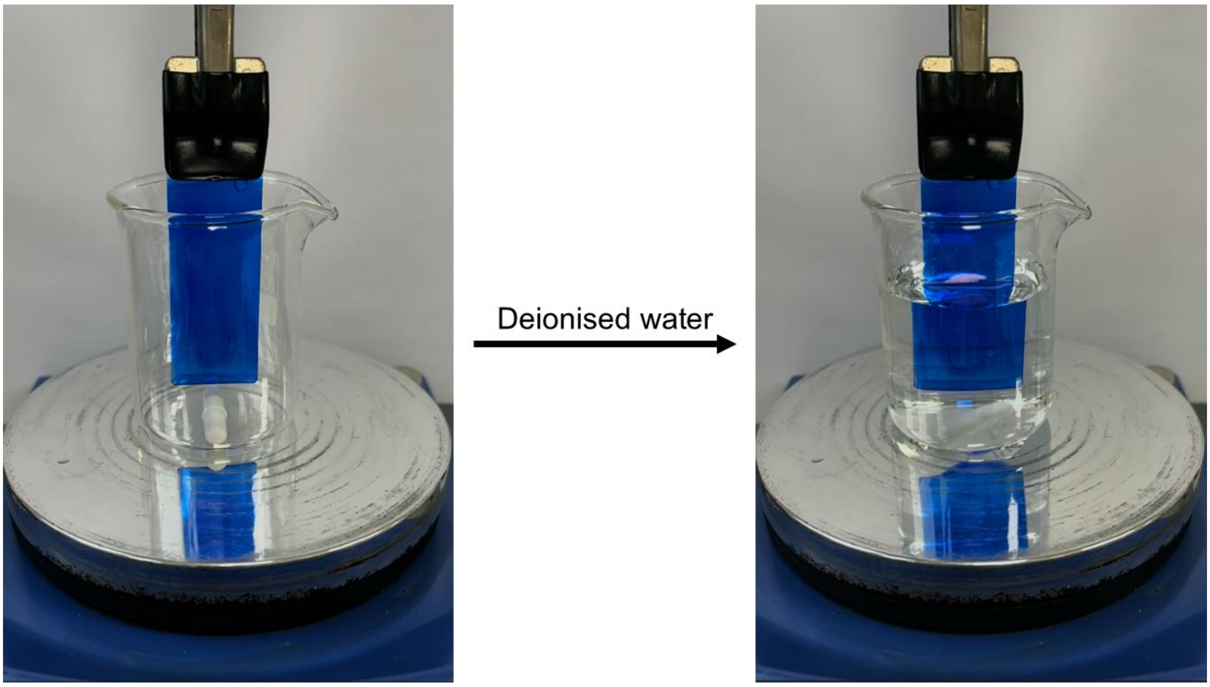

Figure S 88 Treatment of **CEPU3** inkjet formulation on a glass slide using deionised water before and after 10 minutes.

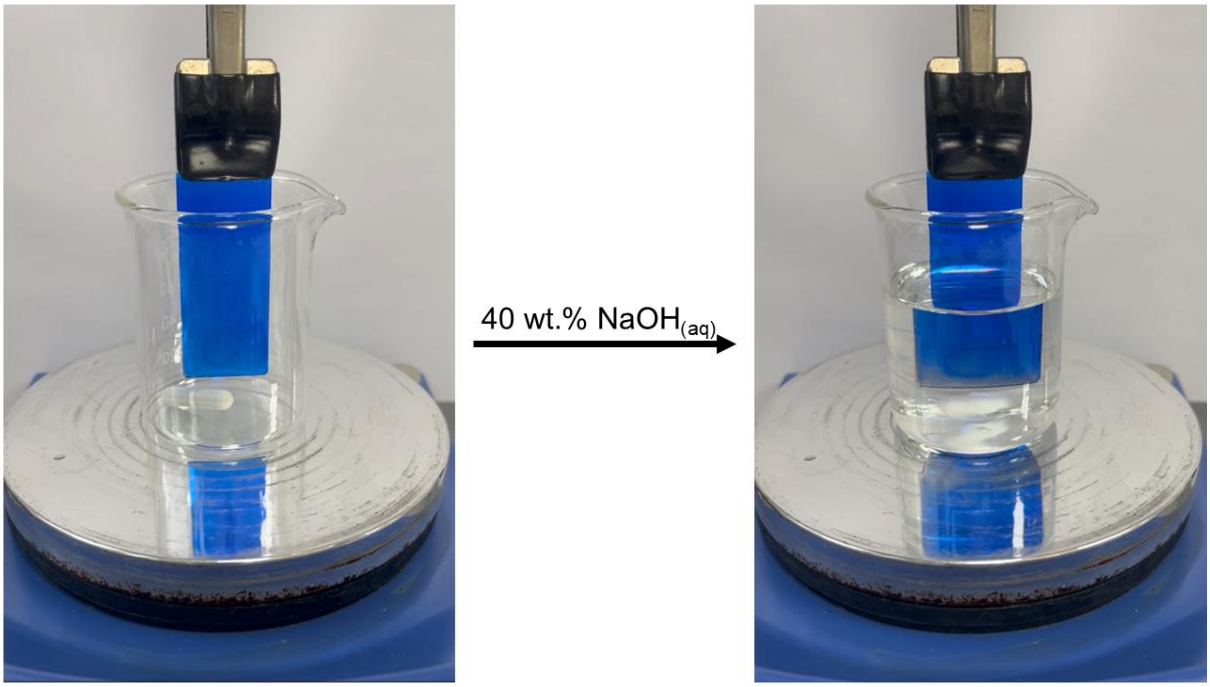

Figure S 89 Debonding of **CEPU3** inkjet formulation form a glass slide using 40 wt.% $NaOH_{(aq)}$ before and after 10 minutes.

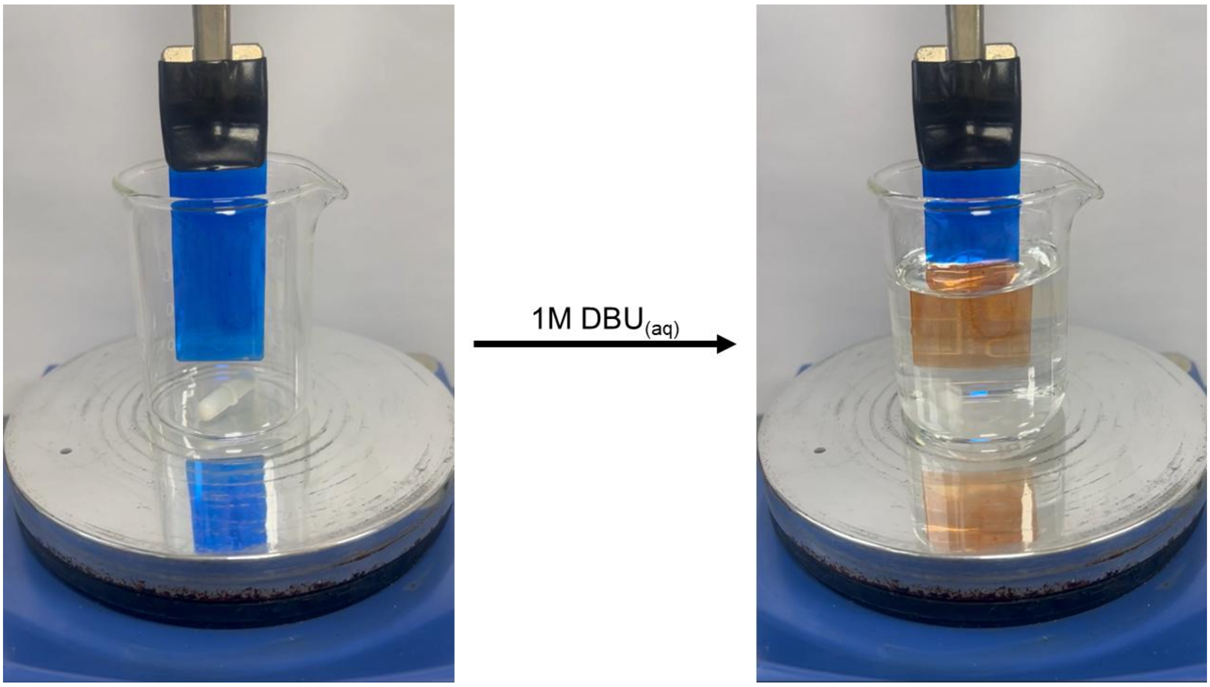

Figure S 90 Debonding of **CEPU3** inkjet formulation form a glass slide using 1 M $DBU_{(aq)}$ before and after 10 minutes.

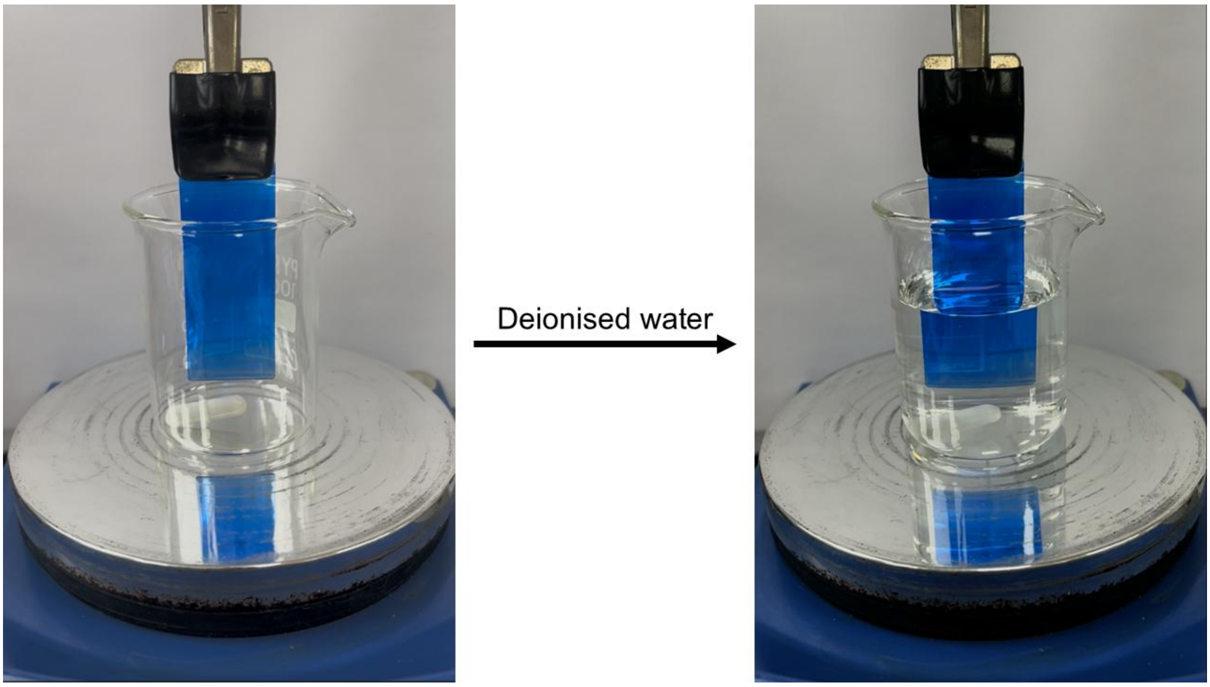

Figure S 91 Treatment of **CEPU5** inkjet formulation on a glass slide using deionised water before and after 10 minutes.

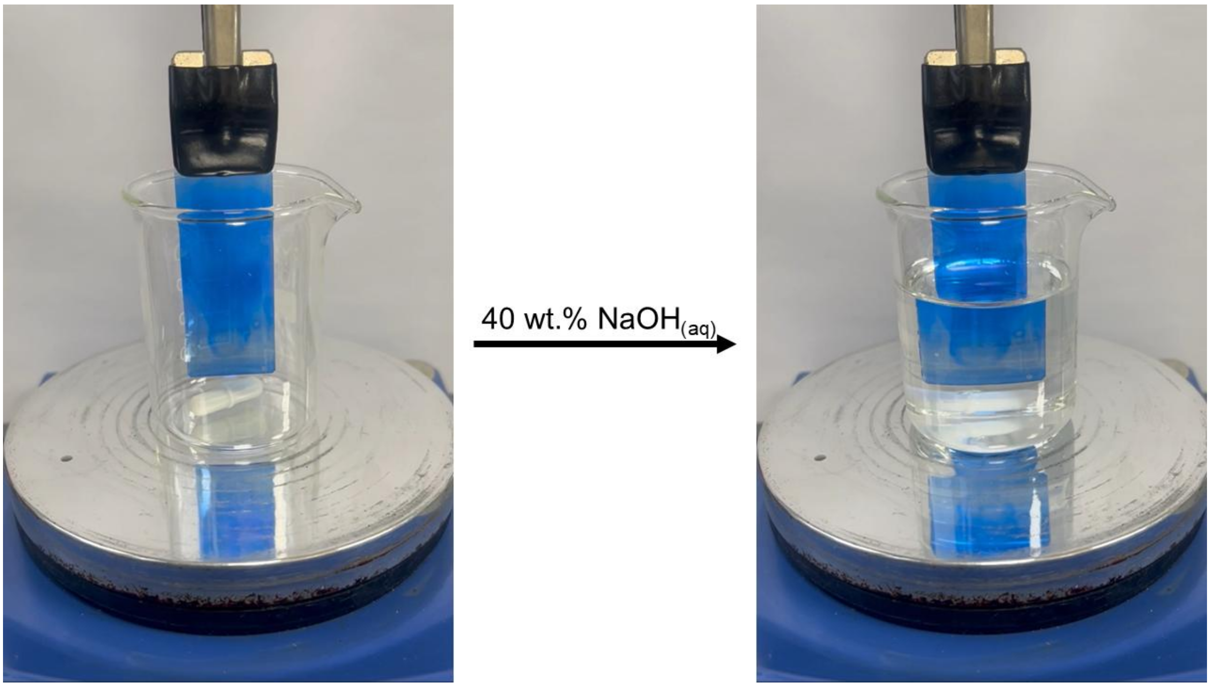

Figure S 92 Debonding of **CEPU5** inkjet formulation form a glass slide using 40 wt.% $NaOH_{(aq)}$ before and after 10 minutes.

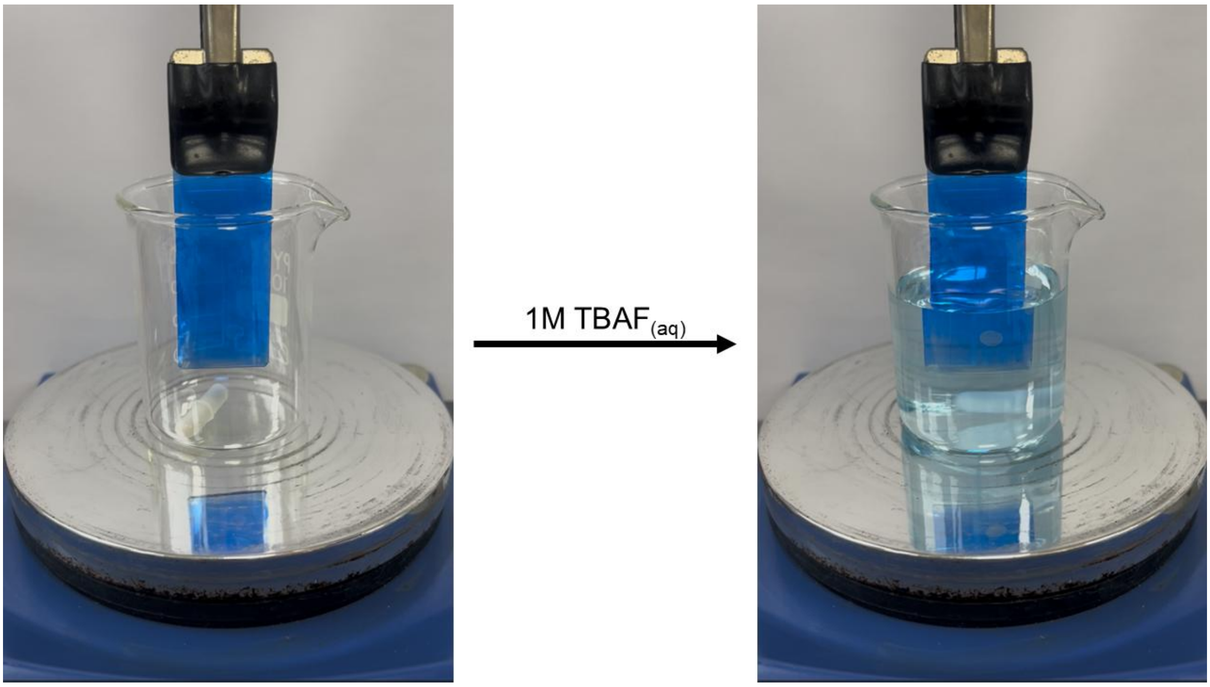

Figure S 93 Debonding of **CEPU5** inkjet formulation form a glass slide using 1 M $TBAF_{(aq)}$ before and after 10 minutes.

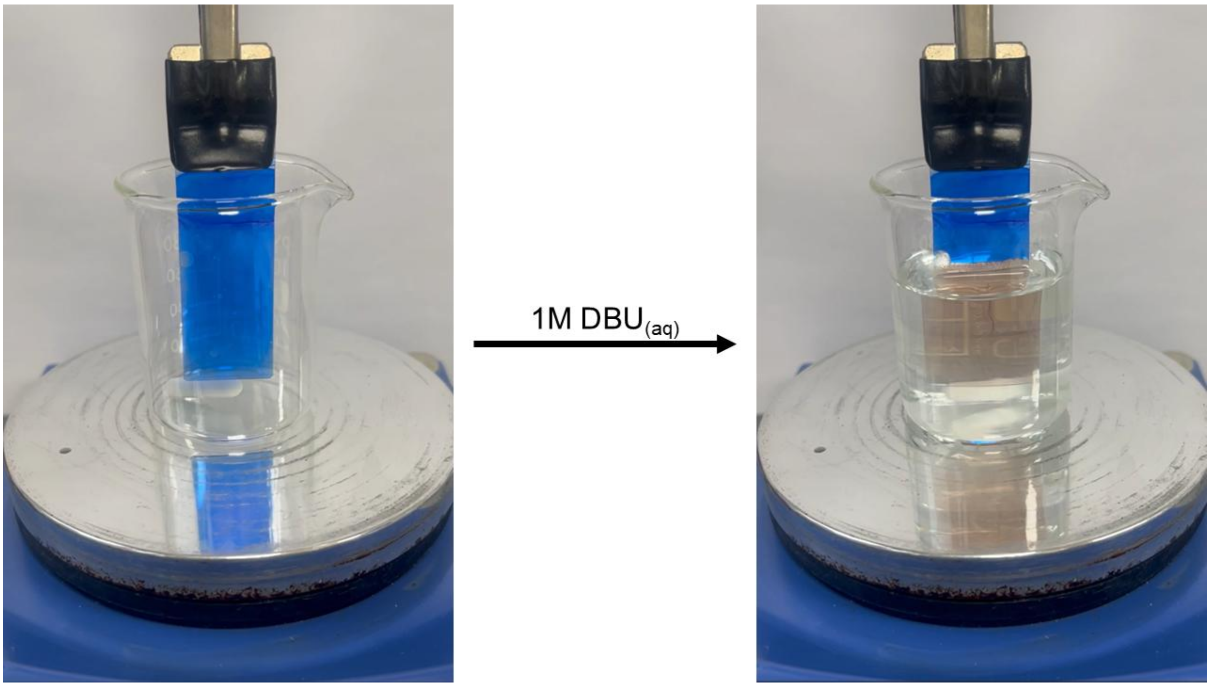

Figure S 94 Debonding of **CEPU5** inkjet formulation form a glass slide using 1 M $DBU_{(aq)}$ before and after 10 minutes.

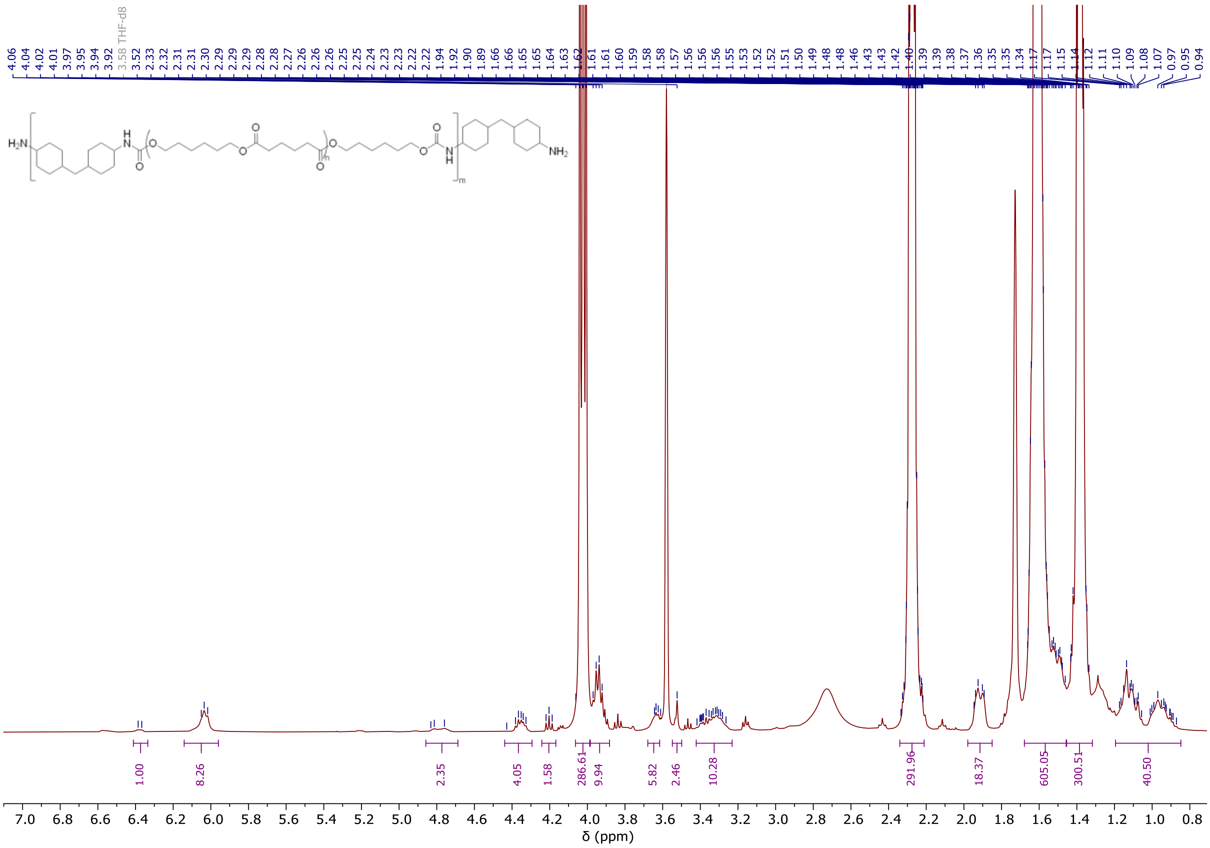

Figure S 95 ^{1}H NMR spectrum of **CEPU5** prepolymer (400 MHz, THF-d_8, 298 K).

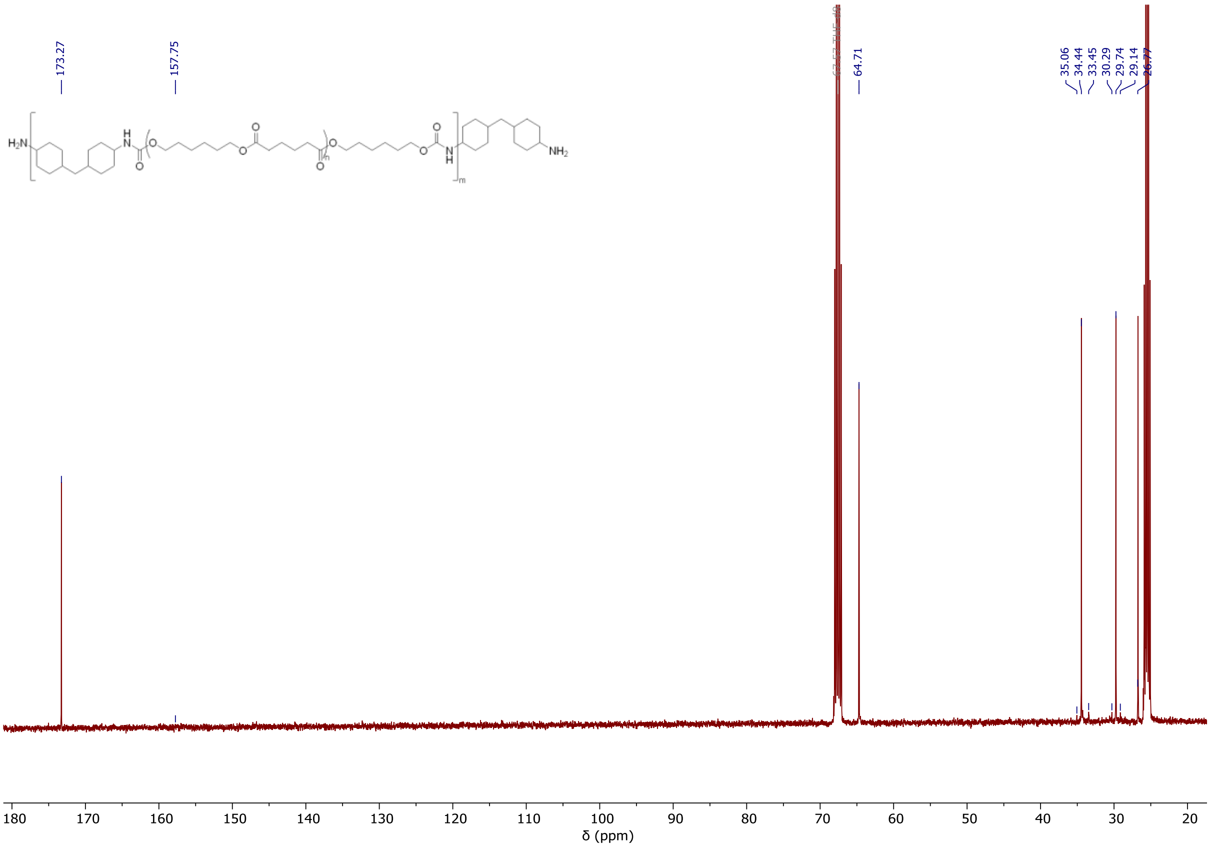

Figure S 96 ^{13}C {H} NMR spectrum of **CEPU5** prepolymer (100 MHz, THF-d_8, 298 K).

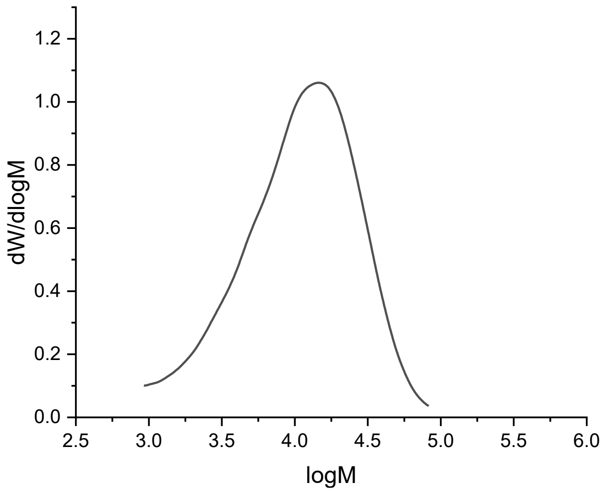

Figure S 97 GPC eluogram of **CEPU5** prepolymer in THF.

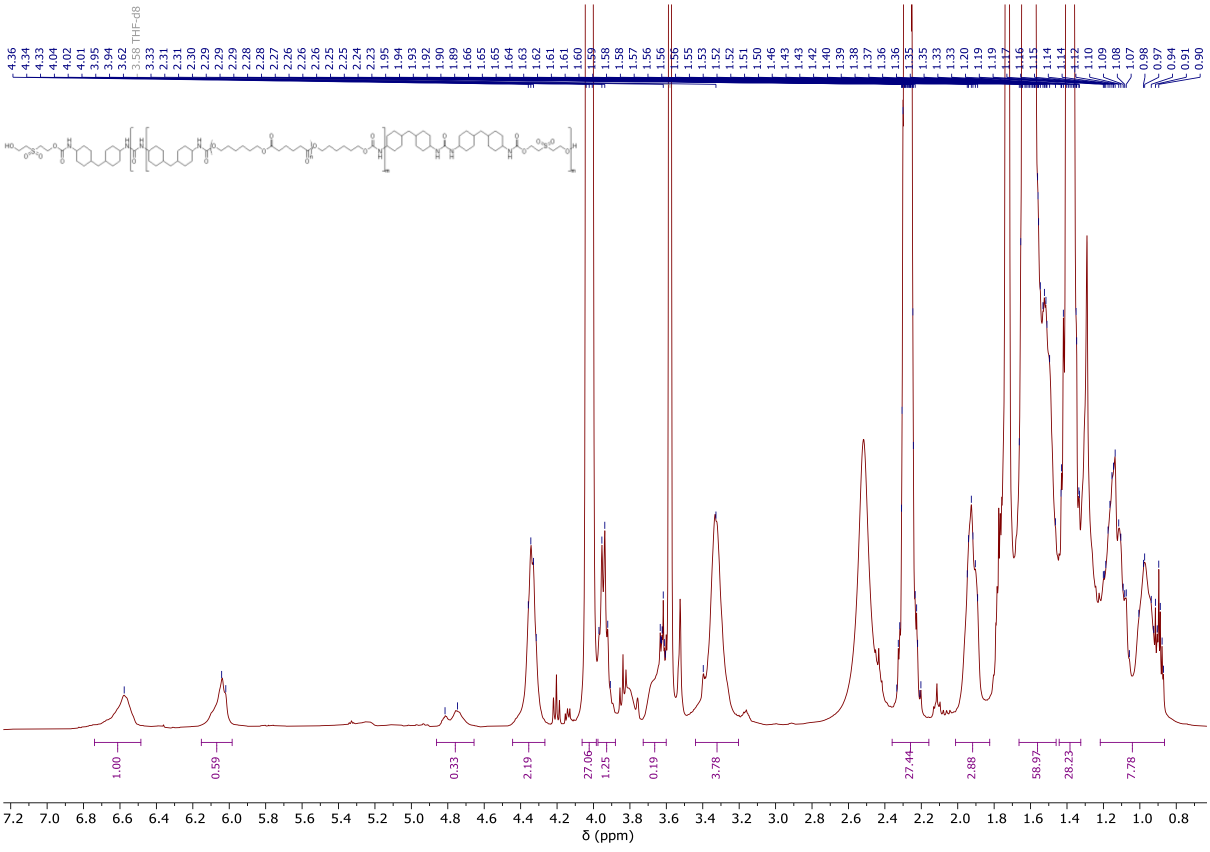

Figure S 98 ^{1}H NMR spectrum of **rCEPU5** (400 MHz, THF-d_8, 298 K).

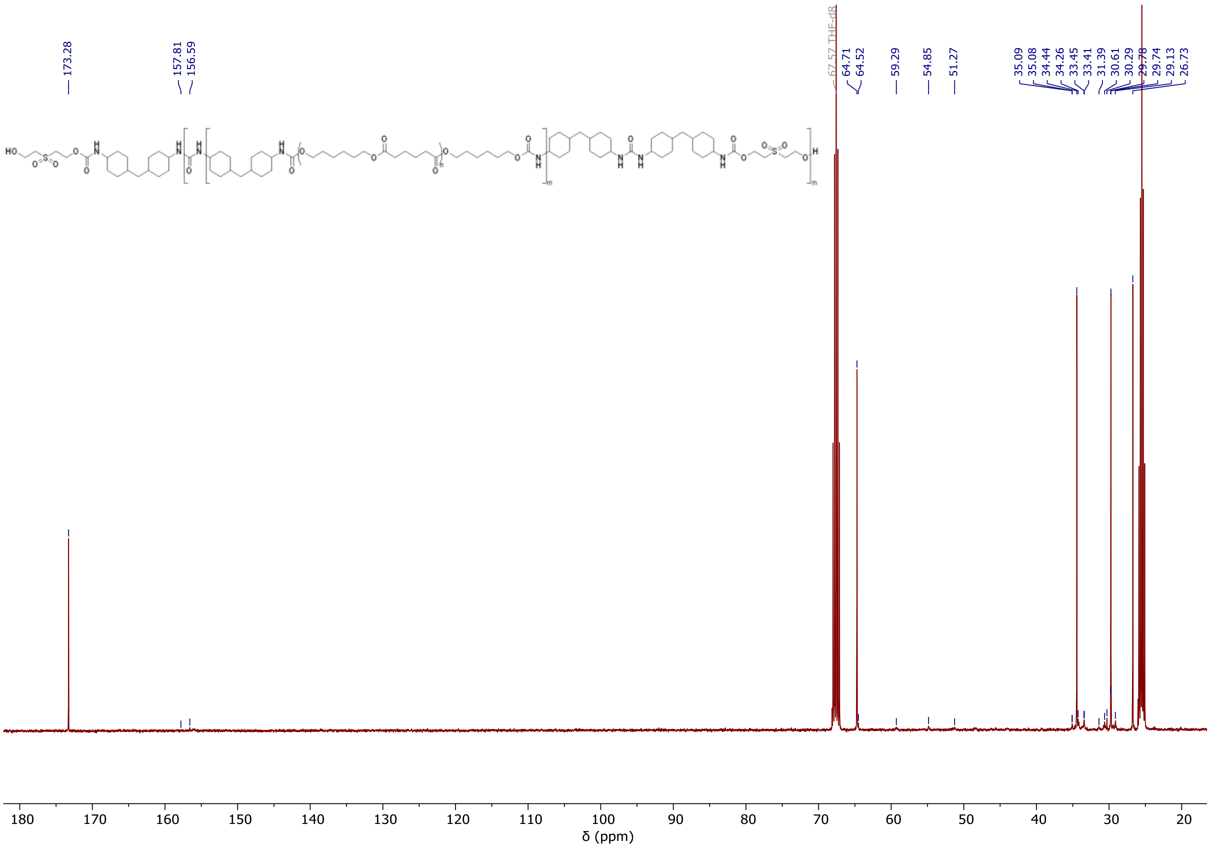

Figure S 99 ^{13}C {H} NMR spectrum of **rCEPU5** (100 MHz, THF-d_8, 298 K).

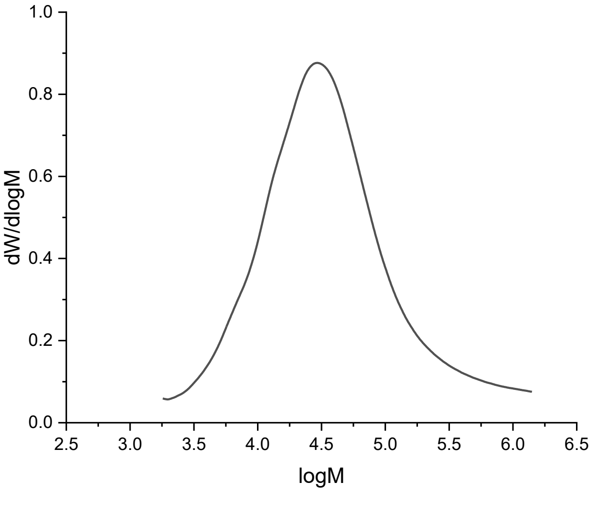

Figure S 100 GPC eluogram of **rCEPU5** in THF.

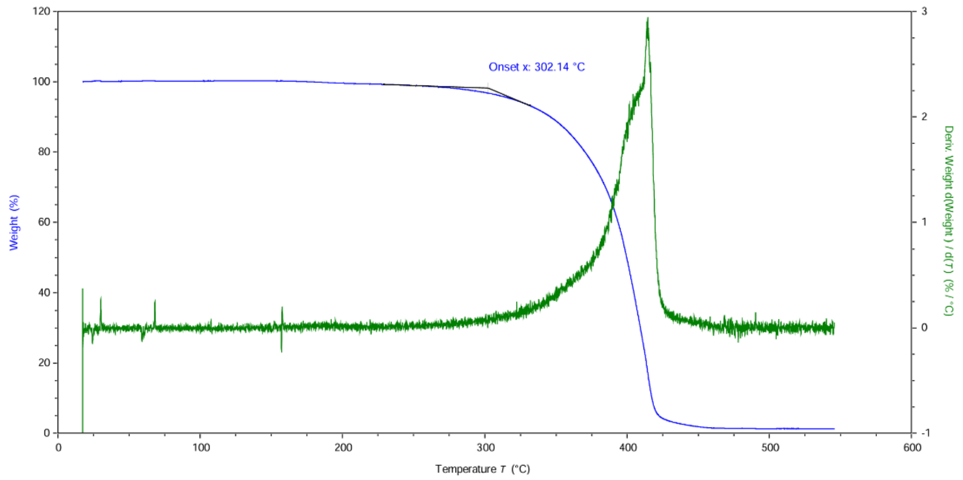

Figure S 101 TGA of **CEPU5** prepolymer at 10 °C min^{-1}.

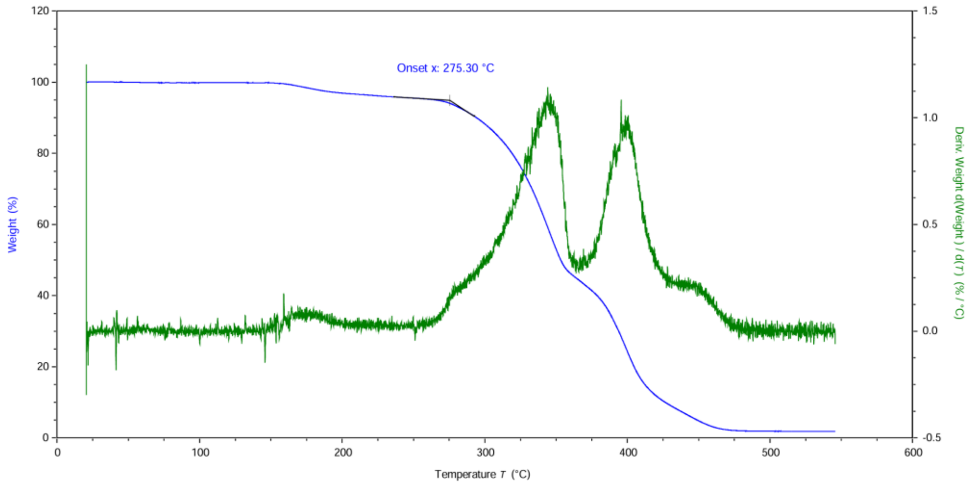

Figure S 102 TGA of **rCEPU5** at 10 °C min^{-1}.

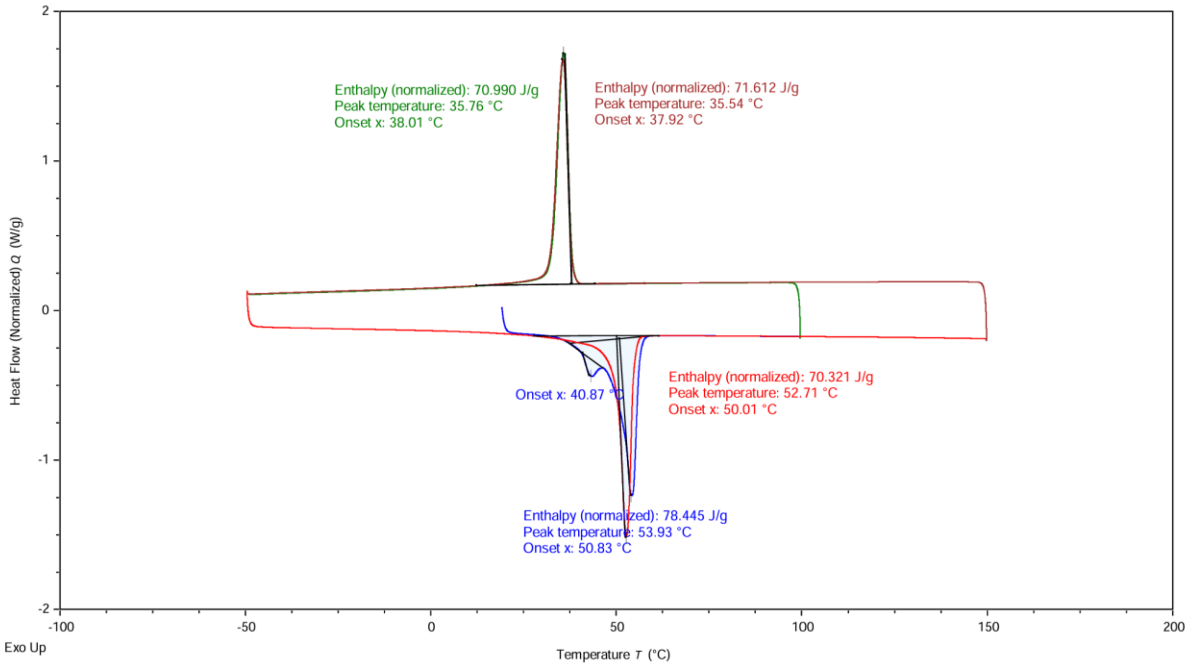

Figure S 103 DSC of **CEPU5** prepolymer at 10 °C min^{-1}, showing the 1st and 2nd heating and cooling cycles.

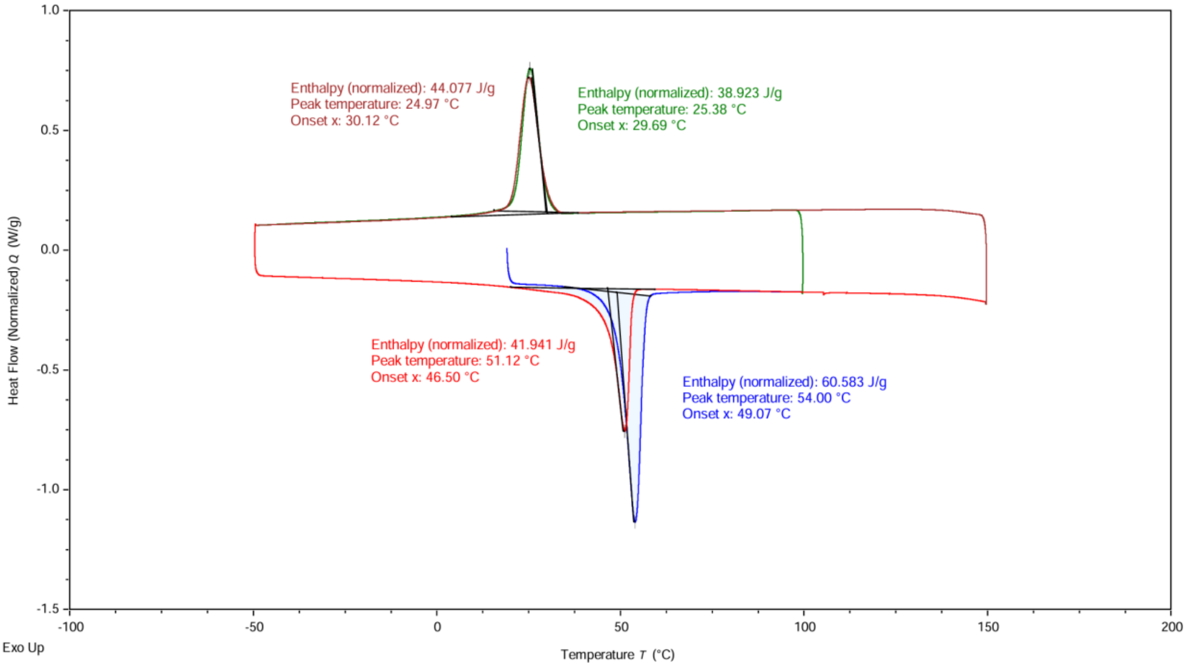

Figure S 104 DSC of **rCEPU5** at 10 °C min^{-1}, showing the 1st and 2nd heating and cooling cycles.

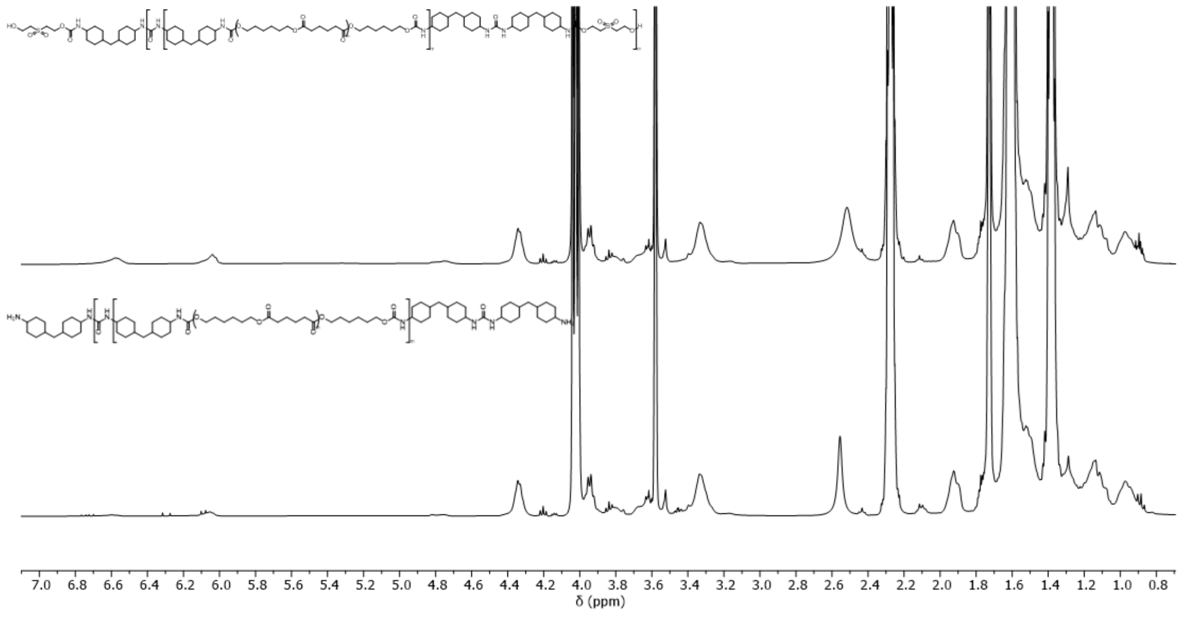

Figure S 105 ^{1}H NMR spectra showing the solution degradation of **rCEPU5** with 40 wt.% NaOD in D_2O, (400 MHz, THF-*d*8). Top spectrum shows the pristine CEPU, bottom spectrum shows the degraded CEPU.

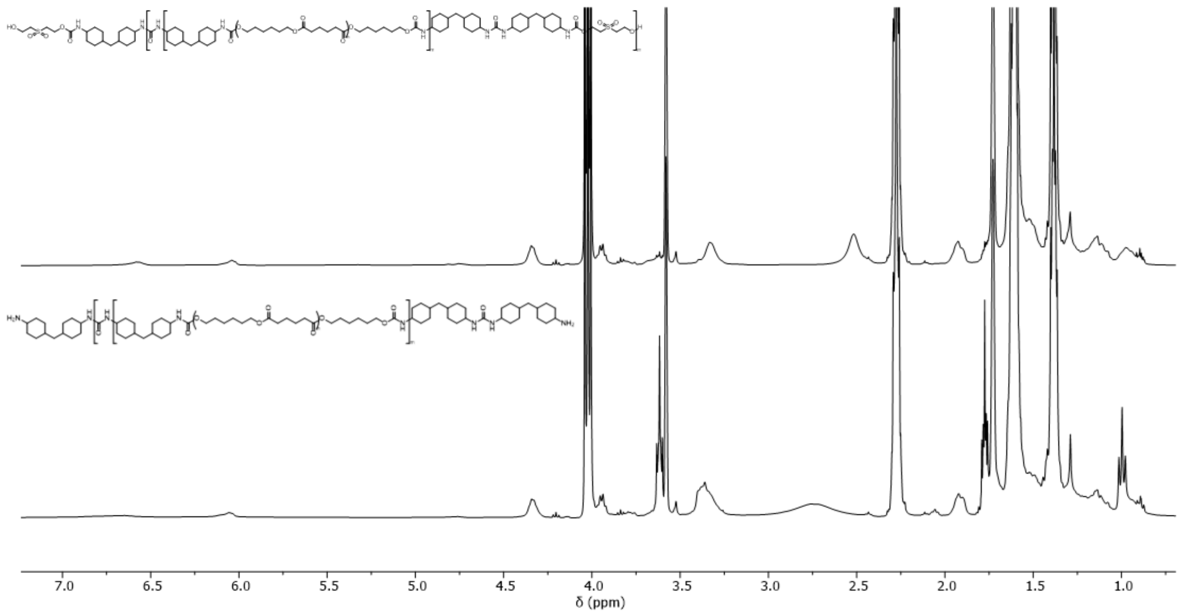

Figure S 106 ^{1}H NMR spectra showing the solution degradation of **rCEPU5** with 1 M TBAF in THF, (400 MHz, THF-*d*8). Top spectrum shows the pristine CEPU, bottom spectrum shows the degraded CEPU.

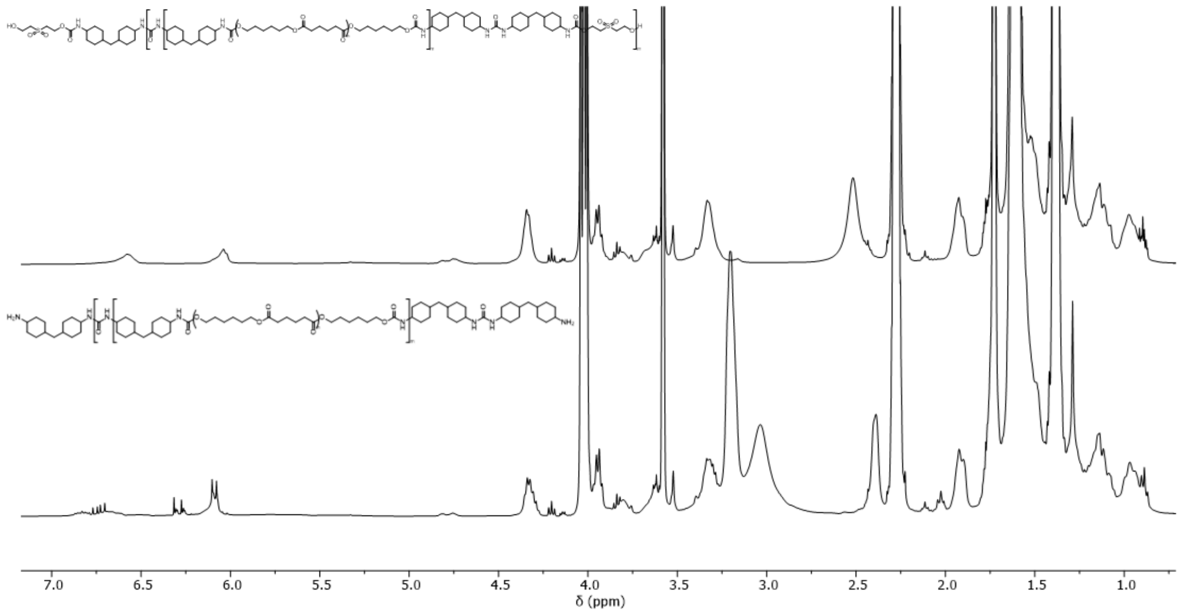

Figure S 107 ^{1}H NMR spectra showing the solution degradation of **rCEPU5** with DBU, (400 MHz, THF-d_8). Top spectrum shows the pristine CEPU, bottom spectrum shows the degraded CEPU.

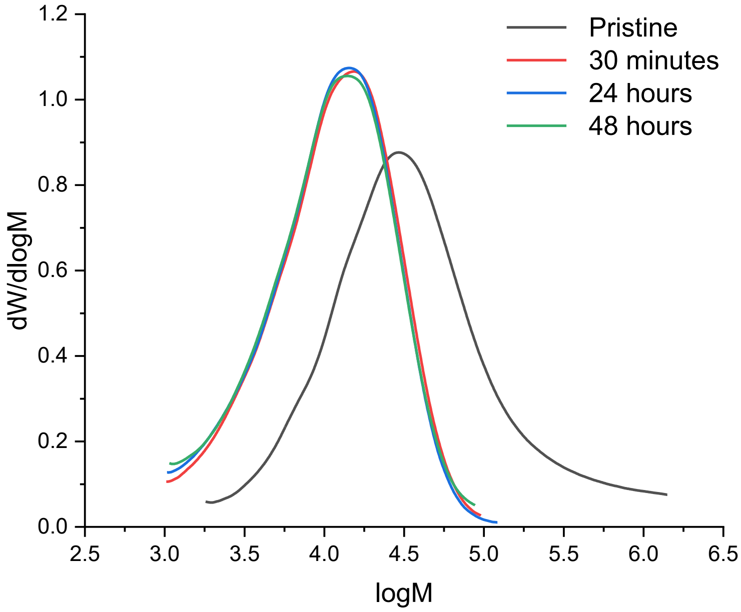

Figure S 108 GPC eluogram of **rCEPU5** in THF as a pristine sample and 30 min, 24 hr, and 48 hr post addition of TBAF.

Table S 7 Shear strength of **CEPU5** and **rCEPU5** on Aluminium, Glass, Wood, high density poly(ethylene) (HDPE), poly(propylene) (PP), Nylon, polyethylene terephthalate (PET), and polyvinyl chloride (PVC). The error shown is the standard deviation between the three repeats of each sample.

Substrate	**CEPU5** (MPa)	**rCEPU5** (MPa)	Difference (%)
Aluminium	0.89 ± 0.05	0.92 ± 0.06	+3
Glass	8.20 ± 0.13	7.41 ± 0.41	-10
Wood	3.78 ± 0.33	3.88 ± 0.11	+3
HDPE	0.16 ± 0.01	0.15 ± 0.00	-4
PP	0.20 ± 0.03	0.21 ± 0.01	+7
Nylon	0.92 ± 0.06	0.93 ± 0.11	0
PET	1.18 ± 0.03	1.34 ± 0.09	+13
PVC	7.35 ± 0.31	6.69 ± 0.27	-9

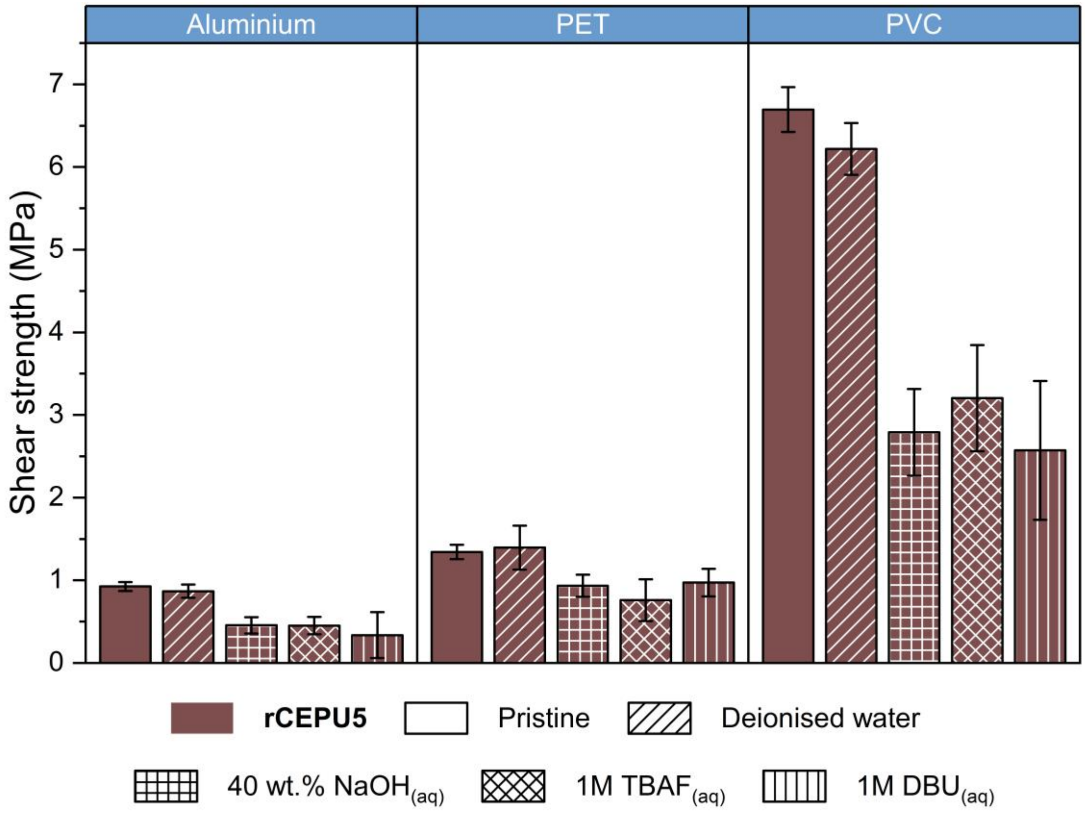

Figure S 109 Shear strength of **rCEPU5** on aluminium, polyethylene terephthalate (PET), and polyvinyl chloride (PVC) as the pristine sample and after exposure to deionised water, 40 wt.% NaOH(aq), 1 M TBAF(aq), and 1 M DBU(aq). The error shown is the standard deviation between the three repeats of each sample.

Table S 8 Shear strength of **rCEPU5** on aluminium, polyethylene terephthalate (PET), and polyvinyl chloride (PVC) as the pristine sample and after exposure to deionised water, 40 wt.% NaOH(aq), 1 M TBAF(aq), and 1 M DBU(aq). Percentages shown are the variation between the pristine and treated samples. The error shown is the standard deviation between the three repeats of each sample.

Substrate	Pristine (MPa)	Deionised water (MPa)	40 wt.% $NaOH_{(aq)}$ (MPa)	1 M $TBAF_{(aq)}$ (MPa)	1 M $DBU_{(aq)}$ (MPa)
Aluminium	0.92 ± 0.06	0.87 ± 0.08<br>-6%	-0.46 ± 0.10<br>-51%	0.45 ± 0.10<br>-51%	0.34 ± 0.28<br>-64%
PET	1.34 ± 0.09	1.40 ± 0.27<br>+4%	0.93 ± 0.13<br>-30%	0.76 ± 0.25<br>-43%	0.97 ± 0.17<br>-28%
PVC	6.69 ± 0.27	6.22 ± 0.31<br>-7%	2.79 ± 0.52<br>-58%	3.20 ± 0.64<br>-52%	2.57 ± 0.84<br>-62%

References

(1) Hyder, M. J.; Godleman, J.; Chippindale, A. M.; Hallett, J. E.; Zinn, T.; Harries, J. L.; Hayes, W. Thermally and Base-Triggered “Debond-on-Demand” Chain-Extended Polyurethane Adhesives. *Macromolecules* **2025**, *58* (1), 681–696. https://doi.org/10.1021/acs.macromol.4c02775.

(2) Oh, Y.; Park, J.; Park, J.-J.; Jeong, S.; Kim, H. Dual Cross-Linked, Polymer Thermosets: Modular Design, Reversible Transformation, and Triggered Debonding. *Chem. Mater.* **2020**, *32* (15), 6384–6391. https://doi.org/10.1021/acs.chemmater.0c01392.

(3) Wu, S.; Cai, C.; Li, F.; Tan, Z.; Dong, S. Supramolecular Adhesive Materials from Natural Acids and Sugars with Tough and Organic Solvent-Resistant Adhesion. *CCS Chemistry* **2021**, *3* (6), 1690–1700. https://doi.org/10.31635/ccschem.020.202000318.

(4) Michal, B. T.; Spencer, E. J.; Rowan, S. J. Stimuli-Responsive Reversible Two-Level Adhesion from a Structurally Dynamic Shape-Memory Polymer. *ACS Appl. Mater. Interfaces* **2016**, *8* (17), 11041–11049. https://doi.org/10.1021/acsami.6b01251.

(5) Sasaki, T.; Hashimoto, S.; Nogami, N.; Sugiyama, Y.; Mori, M.; Naka, Y.; Le, K. V. Dismantlable Thermosetting Adhesives Composed of a Cross-Linkable Poly(Olefin Sulfone) with a Photobase Generator. *ACS Appl. Matter. Interfaces* **2016**, *8* (8), 5580–5585. https://doi.org/10.1021/acsami.5b10110.

(6) Das, S.; Samitsu, S.; Nakamura, Y.; Yamauchi, Y.; Payra, D.; Kato, K.; Naito, M. Thermo-Resettable Cross-Linked Polymers for Reusable/Removable Adhesives. *Polym. Chem.* **2018**, *9* (47), 5559–5565. https://doi.org/10.1039/C8PY01495G.

(7) Damacet, P.; Yarbrough, H. J.; Blelloch, N. D.; Noh, H.-J.; Mirica, K. A. Functional Design of Stimuli-Responsive Poly(Phthalaldehyde)-Based Adhesives: Depolymerization Kinetics and Mechanical Strength Management through Plasticizer Addition. *Polym. Chem.* **2024**, *15* (11), 1112–1122. https://doi.org/10.1039/D3PY01154B.

(8) Jung, S. H.; Choi, G.; Jeong, S.; Park, J.; Yoon, H.; Park, J.-J.; Kim, H. Synthesis of Stimuli-Responsive, Deep Eutectic Solvent-Based Polymer Thermosets for Debondable Adhesives. *ACS Sustainable Chem. Eng.* **2022**, *10* (41), 13816–13824. https://doi.org/10.1021/acssuschemeng.2c04246.

(9) Heinzmann, C.; Coulibaly, S.; Roulin, A.; Fiore, G. L.; Weder, C. Light-Induced Bonding and Debonding with Supramolecular Adhesives. *ACS Appl. Mater. Interfaces* **2014**, *6* (7), 4713–4719. https://doi.org/10.1021/AM405302Z.
